# Data for the subsurface characterization of Pahang River Basin with the application of Transient Electromagnetic geophysical surveys

**DOI:** 10.1016/j.dib.2020.105491

**Published:** 2020-04-23

**Authors:** Hariri Arifin, John Kayode, Khairul Arifin, Zuhar Zahir, Manan Abdullah, Azrin Azmi

**Affiliations:** a Program Geologi, Jabatan Sains Bumi dan Alam Sekitar, Fakulti Sains dan Teknologi, Universiti Kebangsaan Malaysia. 43600 UKM, Bangi, Selangor, Malaysia; b Universiti Teknologi PETRONAS, Institute of Hydrocarbons Recovery, Department of Research and Innovations. Shale Gas Research Group, Persiaran UTP, 32610 Seri Iskandar, Perak Darul Ridzuan, Malaysia; c Universiti Teknologi PETRONAS, Department of Petroleum Geosciences, Persiaran UTP, 32610 Seri Iskandar, Perak Darul Ridzuan, Malaysia; d BeicipFranlabAsia G Tower, Suite 18-16, 199, Jalan Tun Razak, 50450 Kuala Lumpur, Federal Territory of Kuala Lumpur; e Geo Technology Resources Sdn Bhd., 31-1, Jalan Mawar 5B, Taman Mawar, 43900 Sepang, Selangor, Malaysia

**Keywords:** Transient electro-magnetic, massive zones and the weak zones, subsurface structures, Pahang, East coast, Peninsula malaysia

## Abstract

The Transient Electro-Magnetic (TEM) geophysical technique was deployed to map and characterized the subsurface of Pahang River Basin along the East Coast Peninsula Malaysia. The data aimed at differentiating between the massive zones and the weak zones within the region, to also assess and differentiate the subsurface structures and comes up with recommendations for policy decision, formulation and plans on the flooding impact, surface water and groundwater managements, in addition to other environmental related issues ravaging the area. The data presented in this paper, showed the properties of the subsurface rocks underlain the region as beneficial to the Agriculturists; Climatologists; Engineers; Environmentalists; Geoscientists, Hydrologists and Policy formulation officers. The TEM data collection utilized a 100 m x 100 m single loop coil for both the Transmitter (Tx) loop and the Receiver (Rx) loop to produce a total surface area coverage of 10,000 m^2^ per survey line along a single profile. The total area covered in the data extended across an average area of 30 km x 40 km in parts of Maran, Temerloh and Jerantut districts, within the State of Pahang, East Coast, Peninsula Malaysia. The conductivity data recorded varied from -20 mS/m to about 440 mS/m at a maximum depth of about 375 m. On the other hand, the resistivity data recorded varied from 0 Oh-m to about 1000 Oh-m. The information derived from the data are intended for potential abstraction by the Malaysian Groundwater Management Board; the Department of Mineral and Geoscience; Department of Irrigation and Drainage; the Pahang State Water Board, and the Department of Agriculture.

Specifications table

**Table:** 

Subject	Geology, Geophysics and Earth-Surface Processes
Specific subject area	Transient Electromagnetic (TEM) Geophysical Methods, Earth subsurface Processes and Environmental management and policy
Type of data	Excel Image Graph Figure
How data were acquired	ABEM TerraTEM was used to collect the electromagnetic (EM) data, and the Global Positioning System (GPS) data at the location points.
Data format	Raw Analyzed Filtered
Parameters for data collection	To obtain deeper depth penetration, loop size of 100 m x 100 m was selected which produced both high signal to noise ratio as shown in the final model data recorded. The sites covered by the data could be seen to be underlain by relatively low resistivity and high conductive bedrocks with thick alluvium deposit. Application of the EM Algorithms allowed the voltages recorded by the ABEM TerraTEM Equipment to be converted into conductivity and resistivity values using the ArcGIS packages and the location points readings.
Description of data collection	The EM data recorded in the form of conductivity distributions in mS/m, and the corresponding resistivity distributions in Ohm-m, helped to characterized the depths to subsurface structural features of the Pahang River Basin regions as presented in this data article. The high conductive bedrocks with thick alluvium deposit are observed to be dominantly concentrated towards the southwest and the northern part of the area.
Data source location	Institution: Pahang River Basin City/Town/Region: Maran, Temerloh and Jerantut districts in State of Pahang, East Coast Peninsula Malaysia Country: Malaysia Latitude: 391000 - 431000 Northing and longitude: 494000 -to 524000 Easting.
Data accessibility	With the article

## Value of the data

• Transient Electromagnetic (TEM) Method was applied to improve the quality of the data and the depth of investigations whilst prospecting for regional groundwater management with potential for abstraction.
• The data helped to delineates the existence of subsurface structural features thereby providing information on the geology, geometry and the geoelectrical properties of the rocks underlain the region that are beneficial to Agriculturists; Climatologists; Engineers; Environmentalists; Geoscientists, Hydrologists and Policy formulation officers.
• The data helped to identify uncharacteristic subsurface features in relation to the general background and correlated well with the local ground geological conditions for further insights into the climatic issues affecting the hydrological conditions and the people of the region.
• The data is useful for quick assessment; planning; policy formulations, and well-thought-out decisions on the environmental and hydrological challenges as regards flooding and other climate related issues devastating the region.
• The geophysical surveys are uncomplicated, non-expensive, required no galvanic coupling with the ground, thereby makes the adopted preference technique better and faster which could be applied in any part of the world.

## 1 Data description

Subsurface geological conditions have high impacts on the engineering structures established at the earth's surface. Consequently, successful land-use planning of new engineering or urban projects relies on the accurate determination of near-surface structures. The changes of the magnetic field produced across an electrically conductive system create a magnetic induction that also produces a potential difference known as an electromotive force “EMF” across its terminals. This process is called an Electromagnetic (EM) induction [1].

**Fig. 1:**
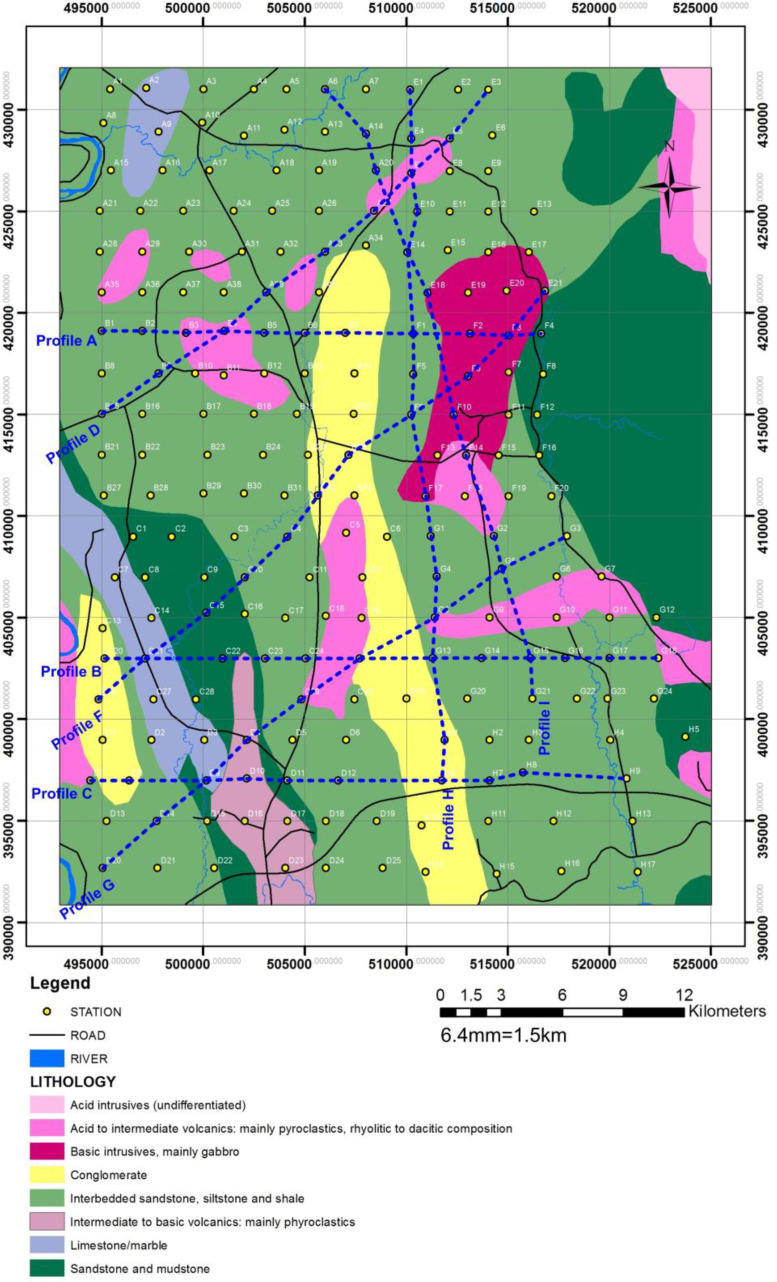
Geological map of the data area, and distributions of the Transient electromagnetic (TEM), survey station points.

**Fig. 2:**
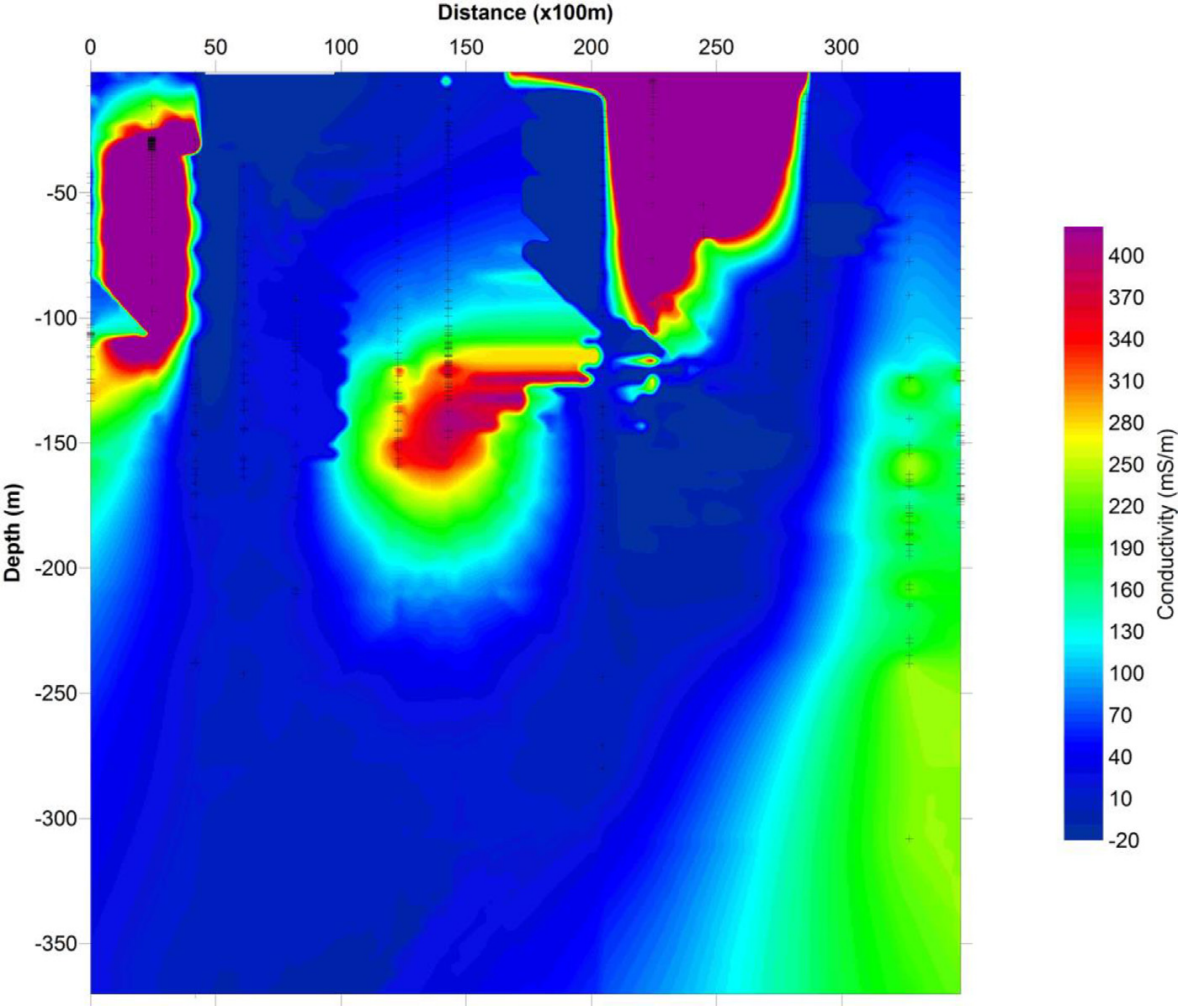
A typical cross section of the subsurface conductivity data distributions along with the survey line.

**Fig. 3:**
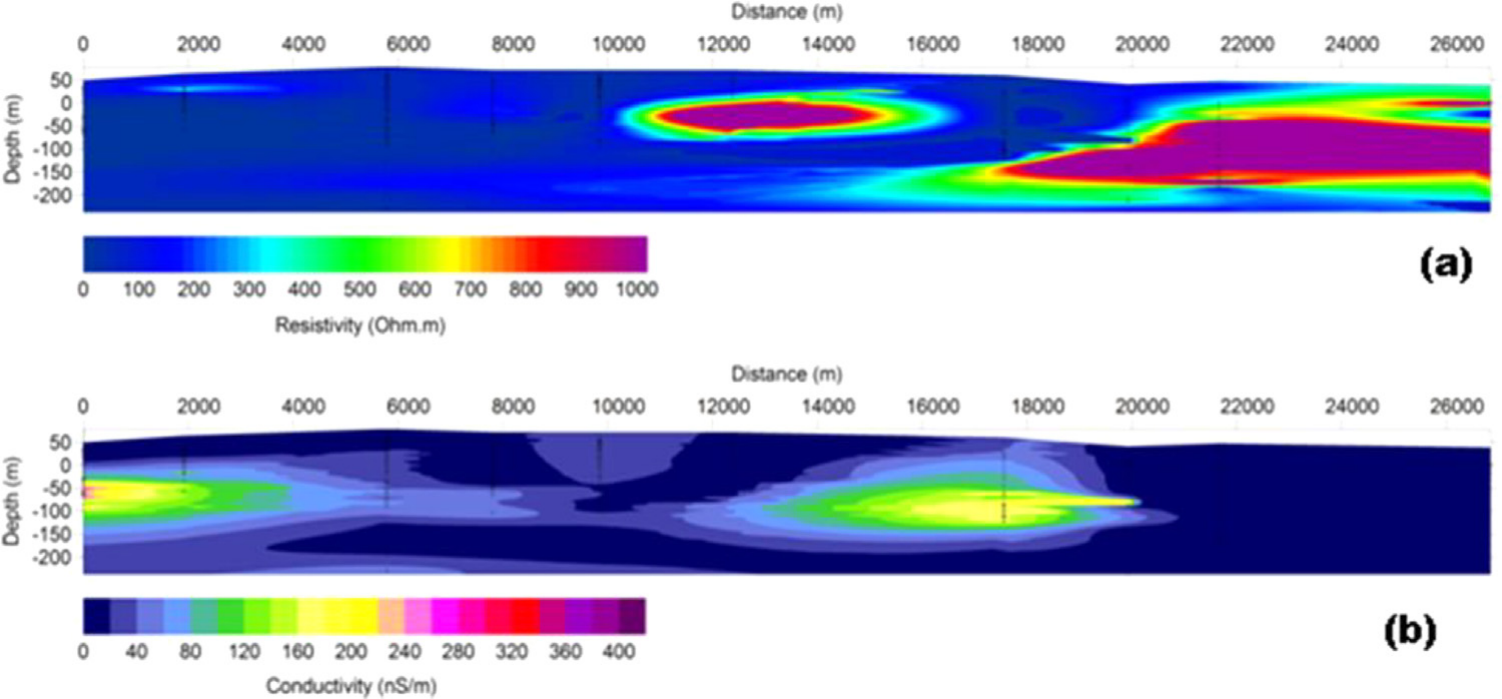
A typical cross section of the subsurface resistivity (a), and its corresponding conductivity (b), data distributions along with the survey line.

The depth penetration was modeled using the cross-sections based on the position of the deepest penetrating point at each observed station along with the survey lines, Fig. 1. Inversion or pseudo sections were generated using the horizontal distance along with the survey lines and the depth of penetration, together with the topographic correction [2], [3]. The target depth of investigation is slightly more than 350 m. The selected loop size of 100 m x 100 m produced both high signal to noise ratio with deeper depth penetration as shown in the final model obtained as the study sites could be seen to be underlain by relatively low resistivity and high conductive rock bodies that possibly represent the bedrock with thick alluvium deposit, as shown in Fig. 2, (conductivity distribution in mS/m), and Fig. 3. (the resistivity in Ohm-m with the corresponding conductivity distribution in mS/m), observed to be dominantly concentrated towards the southwest and the northern part of the area.

The Global Positioning System (GPS) was used to record the coordinates of each station location, as shown in Fig. 1. A total of 200 stations were covered and arranged in grids of approximately 3 km to 5 km within the study area as presented, in the raw data and supplementary data files. The raw data are presented in one dimensional plots of the depths (m) against the subsurface conductivity (mS/m), as a word document file formats for the survey stations A to H. The GPS data in Easting and Northing, the survey positions with their corresponding depths, and conductivity values recorded, are presented in the Excel format for the profiles A to I. Supplementary files of the pre-processed data for spike removal, are presented in the Excel format, and labeled as JENKA A1 to H1. The processed data are presented as 2-D pseudo-sections in JPEG file format. Fig. 7, Fig. 8, Fig. 9, Fig. 10, showed the processed conductivity distributions data recorded with their corresponding resistivity values for the various depths. Hence, all the data are hereby deposited with this article.

The conductivity data recorded in Fig. 2, varied from -20 to about 440 mS/m at a maximum depth of about 375 m. However, the resistivity data recorded in Fig. 3, varied from 0 to 1000 Oh-m at a maximum depth of about 250 m. Massive subsurface sandstone was recorded as indicated.

Along with each profile as presented in the raw and supplementary data files, the 2-D models generated showed different depths of penetration depending on the rate at which the electromagnetic wave propagated into the subsurface stratum that could be demonstrated by the depth of absorption point at each station of measurements. This effect is known as the Skin depth in electromagnetic surveys [4].

## 2 Experimental Design, Materials, and Methods

### Experimental Design

**Fig. 4:**
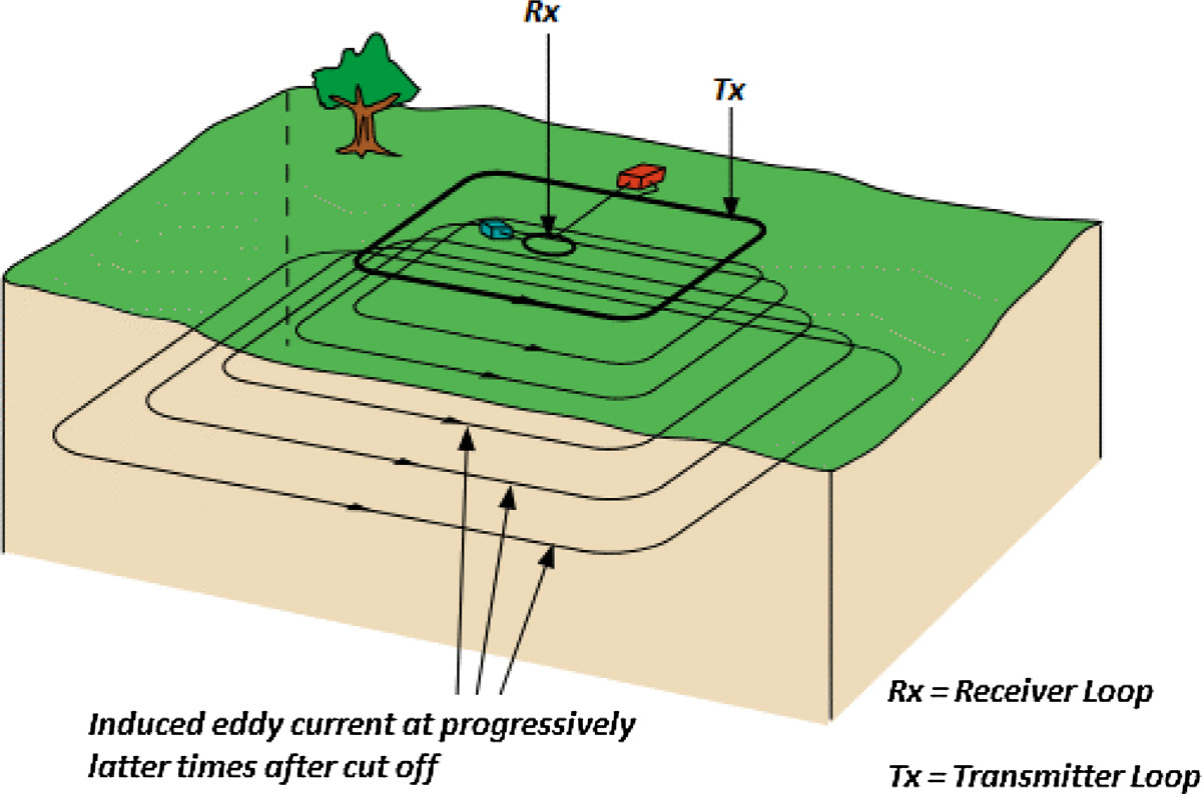
The experimental layout of TEM surveys showing the Transmitter and Receiver Loops.

The parameters used for the EM data collection are 100 m x 100 m single loop coil for both the Tx and the Rx to produce a total surface area coverage of 10,000 m^2^ loop coil per survey. The total area covered in this study stretched across an average area of 1200 Km^2^, (i.e., 30 km x 40 km), Fig. 4. Both the Tx and Rx fitted with a resistor and connected to TerraTEM equipment for the data collection. A 24V, 45 Amp-hour battery was used to power the Equipment. A 3 point average sampling method was adopted for the data collection at each station to ensure data accuracy and consistency as presented in the supplementary files.

**Fig. 5:**
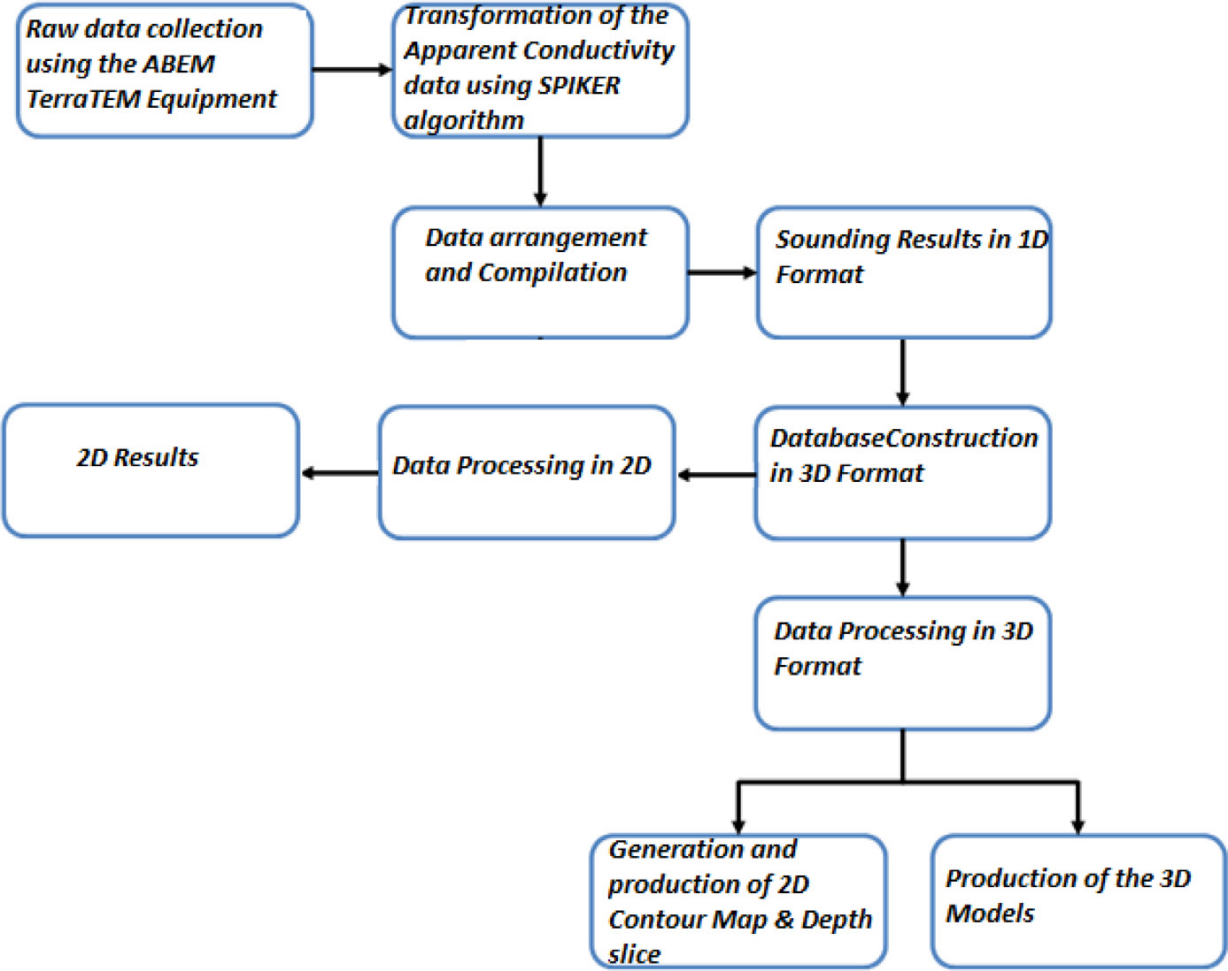
Flow chart showing stages and steps in the data processing and analysis

**Fig. 6:**
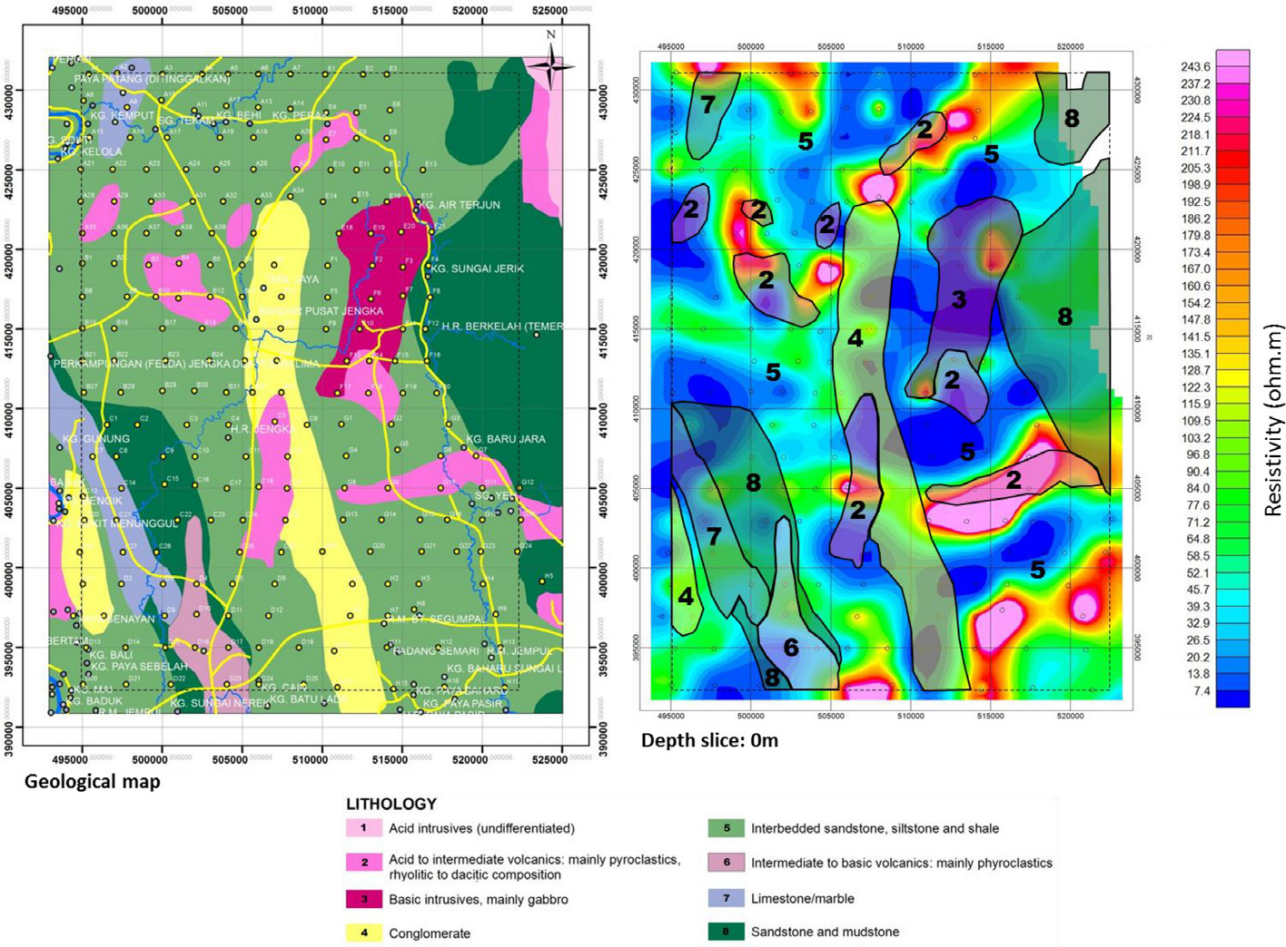
Map of the study area showing integration of the subsurface geology and the TEM anomalies.

**Fig. 7:**
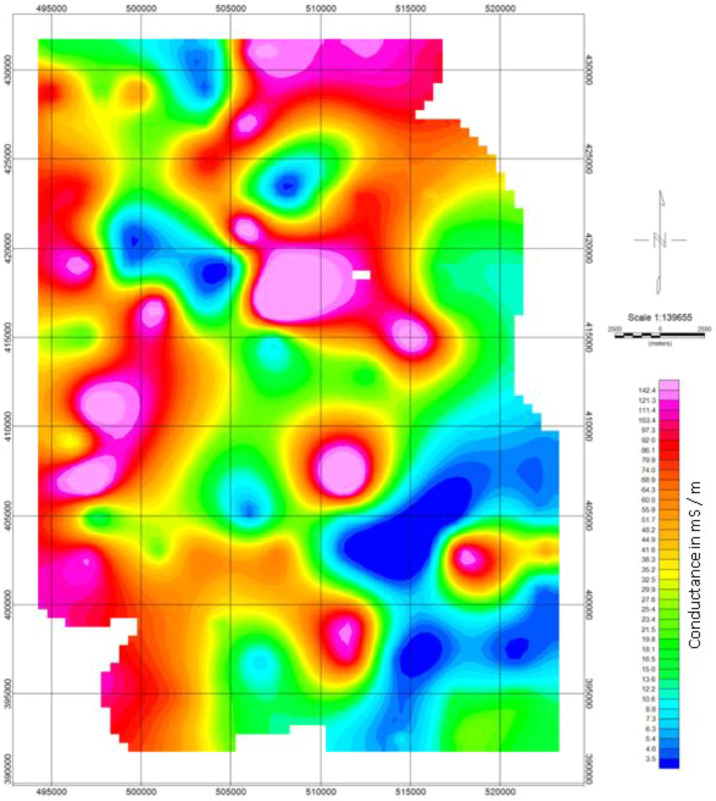
Map of the study area showing the surface conductance distributions in mS/m.

**Fig. 8:**
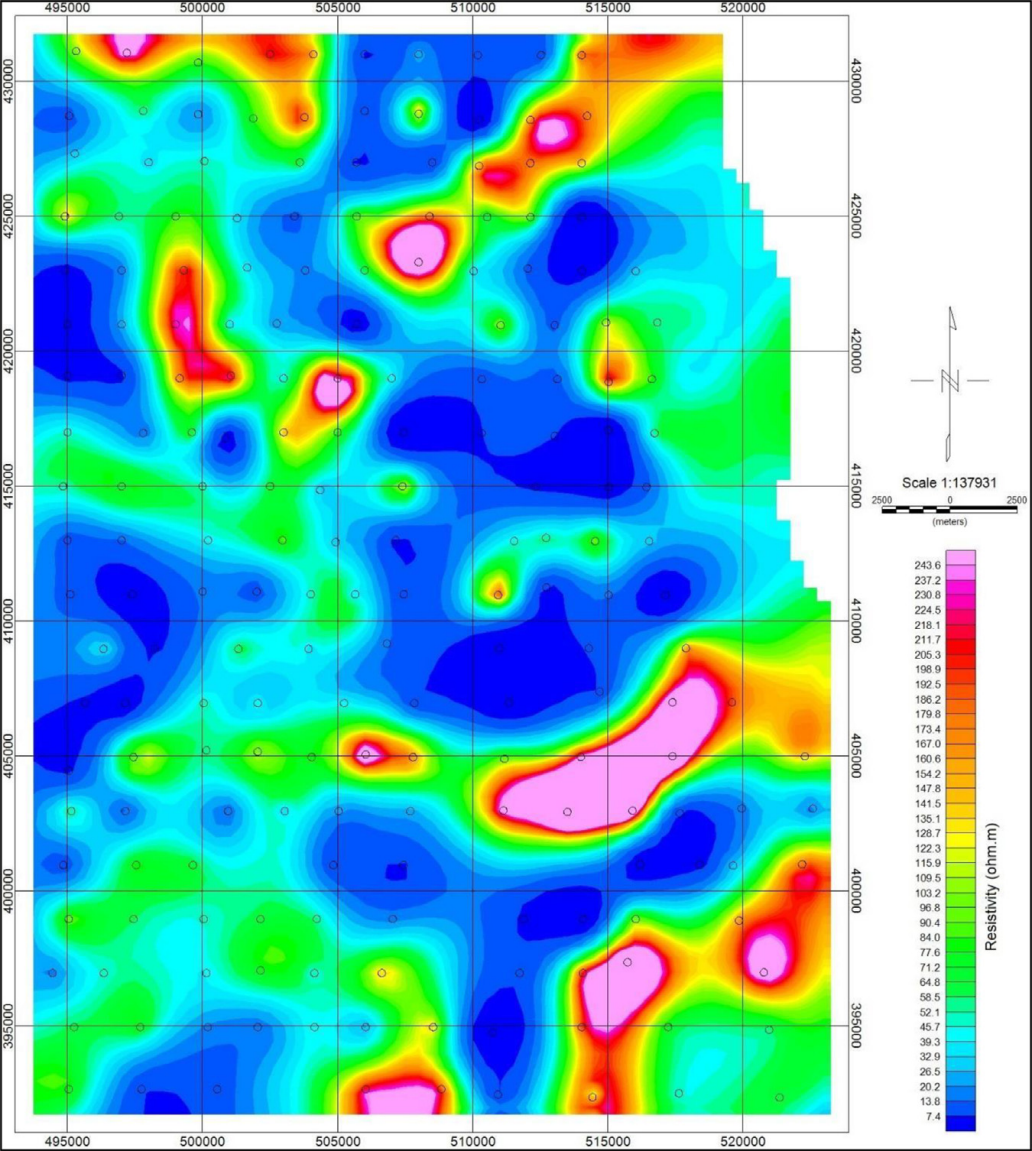
Map of the study area showing the surface resistivity distributions in Ohm-m.

**Fig. 9:**
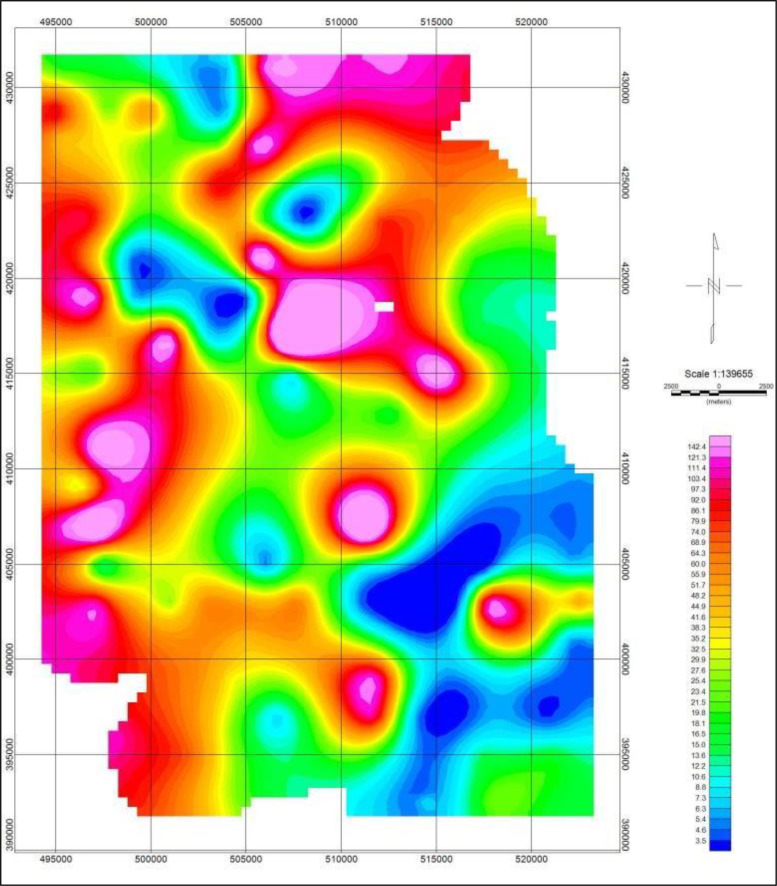
Map of the study area showing the conductance distributions in mS/m at a depth of 50 m.

**Fig. 10:**
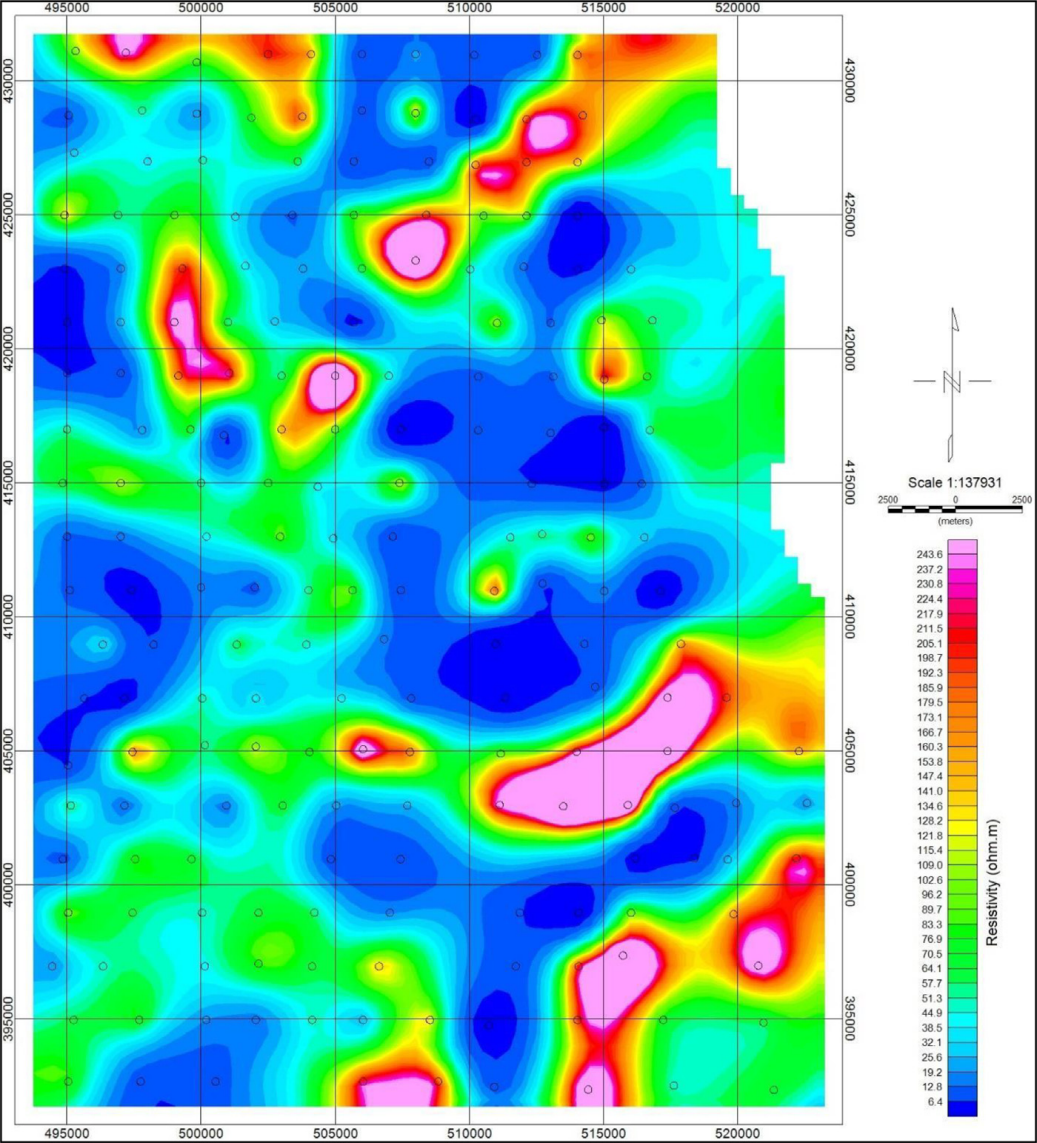
Map of the study area showing the resistivity distributions in Ohm-m at a depth of 50 m.

The presence of subsurface conductive bodies below the receiver loop would be detected and recorded differently from the Tx primary and secondary fields in terms of the direction, intensity, phase, and orientations. The second stage of the data processing, as shown in the flow chat (Fig. 5), involved sorting and compilation of the sounding data in 1-D format. Thereafter, the data was processed to generate the 2-D and 3-D datasets using geostatistical applications that utilized the Kriging interpolation techniques. The Oasis Montaj source parameters from Geosoft Inc. and Surfer software [5], [6], were used to reprocess and produced datasets in the forms of contour maps and pseudo-sections of the subsurface conductivity and resistivity of the stratum as shown in Fig. 6, Fig. 7, Fig. 8, Fig. 9, Fig. 10, with other figures as presented in the supplementary data files. The Kriging interpolation geostatistical techniques [7], was selected for the generations of 2-D and 3-D data maps because of its twofold advantages of given better-estimated values in points or positions where data was not adequately covered or omitted by balancing the data clusters thereby providing efficient overall predictions to produce high-quality data maps as the output.

## 3 Materials and Methods

The transient electromagnetic (TEM) techniques utilize a non-continuous primary field consisting of series of pulses separated by periodic time measurements when current passes through the transmitter (Tx) loop in milliseconds time domain as shown in Fig. 4. The passage of current in the loop set up a primary magnetic around the conducting loop. After the supply is cut off, the presence of the primary magnetic field as a result of the magnetic induction dies off exponentially, which is termed transient. Hence, the magnetic field travels outwardly and downward from the loop with time interval at a velocity, which depends primarily on the conductivity of the subsurface geologic structures underlain the area covered by the loop [8].

The receiver (Rx) loop placed at the centre of the transmitter (Tx) loop, measures the rate of change of the magnetic field per time, which traverses the loop in volts per the transmitted current. The variations between Tx and Rx fields delineate the existence of subsurface conductive structures, thereby providing information on its geometry and the electrical properties. The eddy current produces its own alternating secondary EM fields which travel through the subsurface conductive structures and back to the receiver loop.

The topography of the area is relatively gentle and hilly with surface heights that ranged between about 200 m and 900 m above the mean sea level and flanked northeast of the Picnic Forest area with some areas recorded heights below 200 m around the Jengka Reserve Forest.

The conductivity data distributed within the subsurface stratum as presented in Fig. 7, ranged between 0 and 145 mS/m, while the corresponding resistivity distributions data showed in Fig. 8, varied from 0 to 250 Ohm-m. The other profile data are as presented in the supplementary data files.

Similarly, Fig. 9 and Fig. 10, showed the conductivity distributions data recorded and the corresponding resistivity distributions data at a depth of 50 m, respectively. The respective values recorded varied from 0 to about 145 mS/m for the former, and about 0 to 250 Ohm-m for the later. Details of the depths at 100 m, 150 m, and 200 m are as presented in the supplementary data files.

## Appendix Supplementary materials

Image, application 1

Image, application 2

Image, application 3

Image, application 4

Image, application 5

Image, application 6

Image, application 7

Image, application 8

Image, application 9

Image, application 10

Image, application 11

Image, application 12

Image, application 13

Image, application 14

Image, application 15

Image, application 16

Image, application 17

Image, application 18

Image, application 19

Image, application 20

Image, application 21

Image, application 22

Image, application 23

Image, application 24

Image, application 25

Image, application 26

Image, application 27

Image, application 28

Image, application 29

Image, application 30

Image, application 31

Image, application 32

Image, application 33

Image, application 34

Image, application 35

Image, application 36

Image, application 37

Image, application 38

Image, application 39

Image, application 40

Image, application 41

Image, image 42

Image, image 43

Image, image 44

Image, image 45

Image, image 46

Image, image 47

Image, image 48

Image, image 49
