# Supplementary material for: Data for the subsurface characterization of Pahang River Basin with the application of Transient Electromagnetic geophysical surveys
Source: Data Brief. 2020 Apr 23;30:105491. doi: 10.1016/j.dib.2020.105491 (PMC7191212; doi:10.1016/j.dib.2020.105491)
Supplement: Supplementary file 2 [file mmc2.docx]

| **Station** | **A1** | **Coordinate** | **495312.438°E** |
| --- | --- | --- | --- |
|  |  |  | **431115.281°N** |
|  | | | |

| **Station** | **A2** | **Coordinate** | **497190.656 E** |
| --- | --- | --- | --- |
|  |  |  | **431055.188 N** |
|  | | | |

| **Station** | **A3** | **Coordinate** | **499834.500 E** |
| --- | --- | --- | --- |
|  |  |  | **430691.813 N** |
|  | | | |

| **Station** | **A4** | **Coordinate** | **502499.719 E** |
| --- | --- | --- | --- |
|  |  |  | **430999.156 N** |
|  | | | |

| **Station** | **A5** | **Coordinate** | **504100.438 E** |
| --- | --- | --- | --- |
|  |  |  | **431000.438 N** |
|  | | | |

| **Station** | **A6** | **Coordinate** | **505999.094 E** |
| --- | --- | --- | --- |
|  |  |  | **430999.469 N** |
|  | | | |

| **Station** | **A7** | **Coordinate** | **507999.500 E** |
| --- | --- | --- | --- |
|  |  |  | **430998.406 N** |
|  | | | |

| **Station** | **A8** | **Coordinate** | **495058.844 E** |
| --- | --- | --- | --- |
|  |  |  | **428718.500 N** |
|  | | | |

| **Station** | **A9** | **Coordinate** | **497799.813 E** |
| --- | --- | --- | --- |
|  |  |  | **428900.313 N** |
|  | | | |

| **Station** | **A10** | **Coordinate** | **499837.094 E** |
| --- | --- | --- | --- |
|  |  |  | **428777.375 N** |
|  | | | |

| **Station** | **A11** | **Coordinate** | **501872.500 E** |
| --- | --- | --- | --- |
|  |  |  | **428626.875 N** |
|  | | | |

| **Station** | **A12** | **Coordinate** | **503771.250 E** |
| --- | --- | --- | --- |
|  |  |  | **428668.250 N** |
|  | | | |

| **Station** | **A13** | **Coordinate** | **505999.688 E** |
| --- | --- | --- | --- |
|  |  |  | **428898.969 N** |
|  | | | |

| **Station** | **A14** | **Coordinate** | **508000.031 E** |
| --- | --- | --- | --- |
|  |  |  | **428798.375 N** |
|  | | | |

| **Station** | **A15** | **Coordinate** | **495267.594 E** |
| --- | --- | --- | --- |
|  |  |  | **427325.188 N** |
|  | | | |

| **Station** | **A16** | **Coordinate** | **498000.438 E** |
| --- | --- | --- | --- |
|  |  |  | **427000.313 N** |
|  | | | |

| **Station** | **A17** | **Coordinate** | **500063.938 E** |
| --- | --- | --- | --- |
|  |  |  | **427045.031 N** |
|  | | | |

| **Station** | **A18** | **Coordinate** | **503600.375 E** |
| --- | --- | --- | --- |
|  |  |  | **426999.156 N** |
|  | | | |

| **Station** | **A19** | **Coordinate** | **505698.969 E** |
| --- | --- | --- | --- |
|  |  |  | **426999.719 N** |
|  | | | |

| **Station** | **A20** | **Coordinate** | **508498.938 E** |
| --- | --- | --- | --- |
|  |  |  | **426999.313 N** |
|  | | | |

| **Station** | **A21** | **Coordinate** | **494899.344 E** |
| --- | --- | --- | --- |
|  |  |  | **424998.625 N** |
|  | | | |

| **Station** | **A22** | **Coordinate** | **496899.875 E** |
| --- | --- | --- | --- |
|  |  |  | **424999.156 N** |
|  | | | |

| **Station** | **A23** | **Coordinate** | **499000.375 E** |
| --- | --- | --- | --- |
|  |  |  | **424999.594 N** |
|  | | | |

| **Station** | **A24** | **Coordinate** | **501276.563 E** |
| --- | --- | --- | --- |
|  |  |  | **424931.594 N** |
|  | | | |

| **Station** | **A25** | **Coordinate** | **503399.344 E** |
| --- | --- | --- | --- |
|  |  |  | **425000.281 N** |
|  | | | |

| **Station** | **A26** | **Coordinate** | **505699.719 E** |
| --- | --- | --- | --- |
|  |  |  | **424998.688 N** |
|  | | | |

| **Station** | **A27** | **Coordinate** | **508399.813 E** |
| --- | --- | --- | --- |
|  |  |  | **425000.281 N** |
|  | | | |

| **Station** | **A28** | **Coordinate** | **494899.875 E** |
| --- | --- | --- | --- |
|  |  |  | **422999.438 N** |
|  | | | |

| **Station** | **A29** | **Coordinate** | **497000.460 E** |
| --- | --- | --- | --- |
|  |  |  | **422999.813 N** |
|  | | | |

| **Station** | **A30** | **Coordinate** | **499298.938 E** |
| --- | --- | --- | --- |
|  |  |  | **422999.938 N** |
|  | | | |

| **Station** | **A31** | **Coordinate** | **501649.469 E** |
| --- | --- | --- | --- |
|  |  |  | **423099.531 N** |
|  | | | |

| **Station** | **A32** | **Coordinate** | **503799.813 E** |
| --- | --- | --- | --- |
|  |  |  | **422998.656 N** |
|  | | | |

| **Station** | **A33** | **Coordinate** | **506000.281 E** |
| --- | --- | --- | --- |
|  |  |  | **422999.063 N** |
|  | | | |

| **Station** | **A34** | **Coordinate** | **507999.469 E** |
| --- | --- | --- | --- |
|  |  |  | **423300.156 N** |
|  | | | |

| **Station** | **A35** | **Coordinate** | **495000.344 E** |
| --- | --- | --- | --- |
|  |  |  | **421000.125 N** |
|  | | | |

| **Station** | **A36** | **Coordinate** | **496999.125 E** |
| --- | --- | --- | --- |
|  |  |  | **420998.813 N** |
|  | | | |

| **Station** | **A37** | **Coordinate** | **498999.750 E** |
| --- | --- | --- | --- |
|  |  |  | **420999.375 N** |
|  | | | |

| **Station** | **A38** | **Coordinate** | **501000.375 E** |
| --- | --- | --- | --- |
|  |  |  | **421000.000 N** |
|  | | | |

| **Station** | **A39** | **Coordinate** | **502745.656 E** |
| --- | --- | --- | --- |
|  |  |  | **421030.531 N** |
|  | | | |

| **Station** | **A40** | **Coordinate** | **505699.375 E** |
| --- | --- | --- | --- |
|  |  |  | **420998.500 N** |
|  | | | |
