# Supplementary material for: Data for the subsurface characterization of Pahang River Basin with the application of Transient Electromagnetic geophysical surveys
Source: Data Brief. 2020 Apr 23;30:105491. doi: 10.1016/j.dib.2020.105491 (PMC7191212; doi:10.1016/j.dib.2020.105491)
Supplement: Supplementary file 3 [file mmc3.docx]

| **Station** | **B1** | **Coordinate** | 494999.188 E |
| --- | --- | --- | --- |
|  |  |  | 419100.438 N |
|  | | | |

| **Station** | **B2** | **Coordinate** | 496999.844 E |
| --- | --- | --- | --- |
|  |  |  | 419099.156 N |
|  | | | |

| **Station** | **B3** | **Coordinate** | 499150.281 E |
| --- | --- | --- | --- |
|  |  |  | 418999.969 N |
|  | | | |

| **Station** | **B4** | **Coordinate** | 501049.313 E |
| --- | --- | --- | --- |
|  |  |  | 419100.250 N |
|  | | | |

| **Station** | **B5** | **Coordinate** | 502999.875 E |
| --- | --- | --- | --- |
|  |  |  | 418999.656 N |
|  | | | |

| **Station** | **B6** | **Coordinate** | 505000.531 E |
| --- | --- | --- | --- |
|  |  |  | 419000.344 N |
|  | | | |

| **Station** | **B7** | **Coordinate** | 506999.344 E |
| --- | --- | --- | --- |
|  |  |  | 418999.250 N |
|  | | | |

| **Station** | **B8** | **Coordinate** | 494999.563 E |
| --- | --- | --- | --- |
|  |  |  | 416999.938 N |
|  | | | |

| **Station** | **B9** | **Coordinate** | 497799.781 E |
| --- | --- | --- | --- |
|  |  |  | 416973.438 N |
|  | | | |

| **Station** | **B10** | **Coordinate** | 499598.781 E |
| --- | --- | --- | --- |
|  |  |  | 417000.125 N |
|  | | | |

| **Station** | **B11** | **Coordinate** | 500851.438 E |
| --- | --- | --- | --- |
|  |  |  | 416780.781 N |
|  | | | |

| **Station** | **B12** | **Coordinate** | 503000.563 E |
| --- | --- | --- | --- |
|  |  |  | 416998.625 N |
|  | | | |

| **Station** | **B13** | **Coordinate** | 504999.460 E |
| --- | --- | --- | --- |
|  |  |  | 416999.344 N |
|  | | | |

| **Station** | **B14** | **Coordinate** | 507449.875 E |
| --- | --- | --- | --- |
|  |  |  | 416999.406 N |
|  | | | |

| **Station** | **B15** | **Coordinate** | 494829.813 E |
| --- | --- | --- | --- |
|  |  |  | 414999.188 N |
|  | | | |

| **Station** | **B16** | **Coordinate** | 496999.000 E |
| --- | --- | --- | --- |
|  |  |  | 414999.469 N |
|  | | | |

| **Station** | **B17** | **Coordinate** | 499999.188 E |
| --- | --- | --- | --- |
|  |  |  | 415000.344 N |
|  | | | |

| **Station** | **B18** | **Coordinate** | 502499.688 E |
| --- | --- | --- | --- |
|  |  |  | 415000.219 N |
|  | | | |

| **Station** | **B19** | **Coordinate** | 504352.156 E |
| --- | --- | --- | --- |
|  |  |  | 414859.250 N |
|  | | | |

| **Station** | **B20** | **Coordinate** | 507398.844 E |
| --- | --- | --- | --- |
|  |  |  | 415000.313 N |
|  | | | |

| **Station** | **B21** | **Coordinate** | 494998.750 E |
| --- | --- | --- | --- |
|  |  |  | 412999.750 N |
|  | | | |

| **Station** | **B22** | **Coordinate** | 496999.563 E |
| --- | --- | --- | --- |
|  |  |  | 412998.438 N |
|  | | | |

| **Station** | **B23** | **Coordinate** | 500199.719 E |
| --- | --- | --- | --- |
|  |  |  | 412999.031 N |
|  | | | |

| **Station** | **B24** | **Coordinate** | 502950.125 E |
| --- | --- | --- | --- |
|  |  |  | 413000.375 N |
|  | | | |

| **Station** | **B25** | **Coordinate** | 504924.96 E |
| --- | --- | --- | --- |
|  |  |  | 412929.25 N |
|  | | | |

| **Station** | **B26** | **Coordinate** | 507149.75 E |
| --- | --- | --- | --- |
|  |  |  | 412999.66 N |
|  | | | |

| **Station** | **B27** | **Coordinate** | 495099.25 E |
| --- | --- | --- | --- |
|  |  |  | 410998.594 N |
|  | | | |

| **Station** | **B28** | **Coordinate** | 497399.938 E |
| --- | --- | --- | --- |
|  |  |  | 411000.500 N |
|  | | | |

| **Station** | **B29** | **Coordinate** | 499998.750 E |
| --- | --- | --- | --- |
|  |  |  | 411099.656 N |
|  | | | |

| **Station** | **B30** | **Coordinate** | 501999.594 E |
| --- | --- | --- | --- |
|  |  |  | 411100.281 N |
|  | | | |

| **Station** | **B31** | **Coordinate** | 504000.281 E |
| --- | --- | --- | --- |
|  |  |  | 410999.625 N |
|  | | | |

| **Station** | **B32** | **Coordinate** | 505649.438 E |
| --- | --- | --- | --- |
|  |  |  | 410999.031 N |
|  | | | |

| **Station** | **B33** | **Coordinate** | 507450.375 E |
| --- | --- | --- | --- |
|  |  |  | 411000.063 N |
|  | | | |
