# Supplementary material for: Data for the subsurface characterization of Pahang River Basin with the application of Transient Electromagnetic geophysical surveys
Source: Data Brief. 2020 Apr 23;30:105491. doi: 10.1016/j.dib.2020.105491 (PMC7191212; doi:10.1016/j.dib.2020.105491)
Supplement: Supplementary file 4 [file mmc4.docx]

| **Station** | **C1** | **Coordinate** | **496326.906 E** |
| --- | --- | --- | --- |
|  |  |  | **408975.375 N** |
|  | | | |

| **Station** | **C2** | **Coordinate** | **498226.000 E** |
| --- | --- | --- | --- |
|  |  |  | **408974.250 N** |
|  | | | |

| **Station** | **C3** | **Coordinate** | **501326.344 E** |
| --- | --- | --- | --- |
|  |  |  | **408975.000 N** |
|  | | | |

| **Station** | **C4** | **Coordinate** | **503925.063 E** |
| --- | --- | --- | --- |
|  |  |  | **408974.781 N** |
|  | | | |

| **Station** | **C5** | **Coordinate** | **506825.813 E** |
| --- | --- | --- | --- |
|  |  |  | **409175.000 N** |
|  | | | |

| **Station** | **C6** | **Coordinate** |  |
| --- | --- | --- | --- |
|  |  |  |  |
|  | | | |

| **Station** | **C7** | **Coordinate** | **495638.906 E** |
| --- | --- | --- | --- |
|  |  |  | **406969.906 N** |
|  | | | |

| **Station** | **C8** | **Coordinate** | **497140.063 E** |
| --- | --- | --- | --- |
|  |  |  | **406969.406 N** |
|  | | | |

| **Station** | **C9** | **Coordinate** | **500038.688 E** |
| --- | --- | --- | --- |
|  |  |  | **406968.594 N** |
|  | | | |

| **Station** | **C10** | **Coordinate** | **502039.625 E** |
| --- | --- | --- | --- |
|  |  |  | **406969.219 N** |
|  | | | |

| **Station** | **C11** | **Coordinate** | **505238.125 E** |
| --- | --- | --- | --- |
|  |  |  | **406969.969 N** |
|  | | | |

| **Station** | **C12** | **Coordinate** | **507838.750 E** |
| --- | --- | --- | --- |
|  |  |  | **406969.844 N** |
|  | | | |

| **Station** | **C13** | **Coordinate** | **495038.960 E** |
| --- | --- | --- | --- |
|  |  |  | **404468.688 N** |
|  | | | |

| **Station** | **14** | **Coordinate** | **497438.656 E** |
| --- | --- | --- | --- |
|  |  |  | **404969.781 N** |
|  | | | |

| **Station** | **C15** | **Coordinate** | **500139.656 E** |
| --- | --- | --- | --- |
|  |  |  | **405219.875 N** |
|  | | | |

| **Station** | **C16** | **Coordinate** | **502038.750 E** |
| --- | --- | --- | --- |
|  |  |  | **405169.063 N** |
|  | | | |

| **Station** | **C17** | **Coordinate** | **504039.460 E** |
| --- | --- | --- | --- |
|  |  |  | **404968.906 N** |
|  | | | |

| **Station** | **C18** | **Coordinate** | **506038.656 E** |
| --- | --- | --- | --- |
|  |  |  | **405069.125 N** |
|  | | | |

| **Station** | **C19** | **Coordinate** | **507787.719 E** |
| --- | --- | --- | --- |
|  |  |  | **404968.906 N** |
|  | | | |

| **Station** | **C20** | **Coordinate** | **495140.188 E** |
| --- | --- | --- | --- |
|  |  |  | **402968.688 N** |
|  | | | |

| **Station** | **C21** | **Coordinate** | **497139.344 E** |
| --- | --- | --- | --- |
|  |  |  | **402969.219 N** |
|  | | | |

| **Station** | **C22** | **Coordinate** | **500939.594 E** |
| --- | --- | --- | --- |
|  |  |  | **402968.875 N** |
|  | | | |

| **Station** | **C23** | **Coordinate** | **503038.688 E** |
| --- | --- | --- | --- |
|  |  |  | **402969.406 N** |
|  | | | |

| **Station** | **C24** | **Coordinate** | **505037.844 E** |
| --- | --- | --- | --- |
|  |  |  | **402970.094 N** |
|  | | | |

| **Station** | **C25** | **Coordinate** | **507688.563 E** |
| --- | --- | --- | --- |
|  |  |  | **402969.875 N** |
|  | | | |

| **Station** | **C26** | **Coordinate** | **494839.010 E** |
| --- | --- | --- | --- |
|  |  |  | **400970.000 N** |
|  | | | |

| **Station** | **C27** | **Coordinate** | **497539.750 E** |
| --- | --- | --- | --- |
|  |  |  | **400969.438 N** |
|  | | | |

| **Station** | **C28** | **Coordinate** | **499638.960 E** |
| --- | --- | --- | --- |
|  |  |  | **400969.875 N** |
|  | | | |

| **Station** | **C29** | **Coordinate** | **504838.656 E** |
| --- | --- | --- | --- |
|  |  |  | **400969.375 N** |
|  | | | |

| **Station** | **C30** | **Coordinate** | **507437.594 E** |
| --- | --- | --- | --- |
|  |  |  | **400969.250 N** |
|  | | | |
