# Supplementary material for: Data for the subsurface characterization of Pahang River Basin with the application of Transient Electromagnetic geophysical surveys
Source: Data Brief. 2020 Apr 23;30:105491. doi: 10.1016/j.dib.2020.105491 (PMC7191212; doi:10.1016/j.dib.2020.105491)
Supplement: Supplementary file 5 [file mmc5.docx]

| **Station** | **D1** | **Coordinate** | **495039.469 E** |
| --- | --- | --- | --- |
|  |  |  | **398970.531 N** |
|  | | | |

| **Station** | **D2** | **Coordinate** | **497440.375 E** |
| --- | --- | --- | --- |
|  |  |  | **398970.438 N** |
|  | | | |

| **Station** | **D3** | **Coordinate** | **500039.375 E** |
| --- | --- | --- | --- |
|  |  |  | **398970.094 N** |
|  | | | |

| **Station** | **D4** | **Coordinate** | **502138.563 E** |
| --- | --- | --- | --- |
|  |  |  | **398970.594 N** |
|  | | | |

| **Station** | **D5** | **Coordinate** | **504224.813 E** |
| --- | --- | --- | --- |
|  |  |  | **398974.813 N** |
|  | | | |

| **Station** | **D7** | **Coordinate** | **494440.219 E** |
| --- | --- | --- | --- |
|  |  |  | **396970.469 N** |
|  | | | |

| **Station** | **D8** | **Coordinate** | **496339.531 E** |
| --- | --- | --- | --- |
|  |  |  | **396969.313 N** |
|  | | | |

| **Station** | **D9** | **Coordinate** | **500140.000 E** |
| --- | --- | --- | --- |
|  |  |  | **396970.781 N** |
|  | | | |

| **Station** | **D10** | **Coordinate** | **502139.460 E** |
| --- | --- | --- | --- |
|  |  |  | **397069.063 N** |
|  | | | |

| **Station** | **D11** | **Coordinate** | **504138.500 E** |
| --- | --- | --- | --- |
|  |  |  | **396970.250 N** |
|  | | | |

| **Station** | **D12** | **Coordinate** | **506637.594 E** |
| --- | --- | --- | --- |
|  |  |  | **396970.250 N** |
|  | | | |

| **Station** | **D13** | **Coordinate** | **495240.469 E** |
| --- | --- | --- | --- |
|  |  |  | **394970.031 N** |
|  | | | |

| **Station** | **D14** | **Coordinate** | **497689.625 E** |
| --- | --- | --- | --- |
|  |  |  | **394969.875 N** |
|  | | | |

| **Station** | **D15** | **Coordinate** | **500188.750 E** |
| --- | --- | --- | --- |
|  |  |  | **394969.688 N** |
|  | | | |

| **Station** | **D16** | **Coordinate** | **502038.125 E** |
| --- | --- | --- | --- |
|  |  |  | **394970.563 N** |
|  | | | |

| **Station** | **D17** | **Coordinate** | **504139.219 E** |
| --- | --- | --- | --- |
|  |  |  | **394969.250 N** |
|  | | | |

| **Station** | **D18** | **Coordinate** | **506038.563 E** |
| --- | --- | --- | --- |
|  |  |  | **394970.125 N** |
|  | | | |

| **Station** | **D19** | **Coordinate** | **508537.688 E** |
| --- | --- | --- | --- |
|  |  |  | **394970.156 N** |
|  | | | |

| **Station** | **D20** | **Coordinate** | **495040.594 E** |
| --- | --- | --- | --- |
|  |  |  | **392670.844 N** |
|  | | | |

| **Station** | **D21** | **Coordinate** | **497739.719 E** |
| --- | --- | --- | --- |
|  |  |  | **392670.281 N** |
|  | | | |

| **Station** | **D22** | **Coordinate** | **500538.813 E** |
| --- | --- | --- | --- |
|  |  |  | **392669.656 N** |
|  | | | |

| **Station** | **D23** | **Coordinate** |  |
| --- | --- | --- | --- |
|  |  |  |  |
|  | | | |

| **Station** | **D24** | **Coordinate** | **506038.875 E** |
| --- | --- | --- | --- |
|  |  |  | **392670.625 N** |
|  | | | |

| **Station** | **D25** | **Coordinate** | **508837.969 E** |
| --- | --- | --- | --- |
|  |  |  | **392670.219 N** |
|  | | | |
