# Supplementary material for: Data for the subsurface characterization of Pahang River Basin with the application of Transient Electromagnetic geophysical surveys
Source: Data Brief. 2020 Apr 23;30:105491. doi: 10.1016/j.dib.2020.105491 (PMC7191212; doi:10.1016/j.dib.2020.105491)
Supplement: Supplementary file 6 [file mmc6.docx]

| **Station** | **E1** | **Coordinate** | **510188.656 E** |
| --- | --- | --- | --- |
|  |  |  | **430967.625 N** |
|  | | | |

| **Station** | **E2** | **Coordinate** | **512536.980 E** |
| --- | --- | --- | --- |
|  |  |  | **430967.990 N** |
|  | | | |

| **Station** | **E3** | **Coordinate** | **514037.781 E** |
| --- | --- | --- | --- |
|  |  |  | **430967.750 N** |
|  | | | |

| **Station** | **E4** | **Coordinate** | **510237.031 E** |
| --- | --- | --- | --- |
|  |  |  | **428568.531 N** |
|  | | | |

| **Station** | **E5** | **Coordinate** | **512137.594 E** |
| --- | --- | --- | --- |
|  |  |  | **428567.688 N** |
|  | | | |

| **Station** | **E6** | **Coordinate** | **514236.375 E** |
| --- | --- | --- | --- |
|  |  |  | **428717.688 N** |
|  | | | |

| **Station** | **E7** | **Coordinate** | **510238.313 E** |
| --- | --- | --- | --- |
|  |  |  | **426867.844 N** |
|  | | | |

| **Station** | **E8** | **Coordinate** | **512137.188 E** |
| --- | --- | --- | --- |
|  |  |  | **426968.344 N** |
|  | | | |

| **Station** | **E9** | **Coordinate** | **514037.781 E** |
| --- | --- | --- | --- |
|  |  |  | **426967.500 N** |
|  | | | |

| **Station** | **E10** | **Coordinate** | **510537.281 E** |
| --- | --- | --- | --- |
|  |  |  | **424967.719 N** |
|  | | | |

| **Station** | **E11** | **Coordinate** | **512138.094 N** |
| --- | --- | --- | --- |
|  |  |  | **424967.313 N** |
|  | | | |

| **Station** | **E12** | **Coordinate** | **514036.844 E** |
| --- | --- | --- | --- |
|  |  |  | **424968.344 N** |
|  | | | |

| **Station** | **E14** | **Coordinate** | **510038.438 E** |
| --- | --- | --- | --- |
|  |  |  | **422967.406 N** |
|  | | | |

| **Station** | **E15** | **Coordinate** | **512037.313 E** |
| --- | --- | --- | --- |
|  |  |  | **423067.750 N** |
|  | | | |

| **Station** | **E16** | **Coordinate** | **514037.781 E** |
| --- | --- | --- | --- |
|  |  |  | **422967.313 N** |
|  | | | |

| **Station** | **E17** | **Coordinate** | **516036.531 E** |
| --- | --- | --- | --- |
|  |  |  | **422968.219 N** |
|  | | | |

| **Station** | **E18** | **Coordinate** | **511036.813 E** |
| --- | --- | --- | --- |
|  |  |  | **420968.656 N** |
|  | | | |

| **Station** | **E19** | **Coordinate** | **513037.469 E** |
| --- | --- | --- | --- |
|  |  |  | **420967.656 N** |
|  | | | |

| **Station** | **E20** | **Coordinate** | **514936.438 E** |
| --- | --- | --- | --- |
|  |  |  | **421068.219 N** |
|  | | | |

| **Station** | **E21** | **Coordinate** | **516837.125 E** |
| --- | --- | --- | --- |
|  |  |  | **421067.469 N** |
|  | | | |
