# Supplementary material for: Data for the subsurface characterization of Pahang River Basin with the application of Transient Electromagnetic geophysical surveys
Source: Data Brief. 2020 Apr 23;30:105491. doi: 10.1016/j.dib.2020.105491 (PMC7191212; doi:10.1016/j.dib.2020.105491)
Supplement: Supplementary file 7 [file mmc7.docx]

| **Station** | **F1** | **Coordinate** | **510338.094 E** |
| --- | --- | --- | --- |
|  |  |  | **418968.625 N** |
|  | | | |

| **Station** | **F2** | **Coordinate** | **513138.313 E** |
| --- | --- | --- | --- |
|  |  |  | **418968.344 N** |
|  | | | |

| **Station** | **F3** | **Coordinate** | **515037.031 E** |
| --- | --- | --- | --- |
|  |  |  | **418868.063 N** |
|  | | | |

| **Station** | **F4** | **Coordinate** | **516636.250 E** |
| --- | --- | --- | --- |
|  |  |  | **418969.063 N** |
|  | | | |

| **Station** | **F5** | **Coordinate** | **510337.094 E** |
| --- | --- | --- | --- |
|  |  |  | **416967.625 N** |
|  | | | |

| **Station** | **F6** | **Coordinate** | **513037.250 E** |
| --- | --- | --- | --- |
|  |  |  | **416867.969 N** |
|  | | | |

| **Station** | **F7** | **Coordinate** | **515036.41 E** |
| --- | --- | --- | --- |
|  |  |  | **417067.88 N** |
|  | | | |

| **Station** | **F8** | **Coordinate** | **516737.156 E** |
| --- | --- | --- | --- |
|  |  |  | **416967.875 N** |
|  | | | |

| **Station** | **F10** | **Coordinate** | **512336.844 E** |
| --- | --- | --- | --- |
|  |  |  | **414969.280 N** |
|  | | | |

| **Station** | **F11** | **Coordinate** | **515037.219 E** |
| --- | --- | --- | --- |
|  |  |  | **414969.188 N** |
|  | | | |

| **Station** | **F12** | **Coordinate** | **516436.438 E** |
| --- | --- | --- | --- |
|  |  |  | **414969.125 N** |
|  | | | |

| **Station** | **F13** | **Coordinate** | **511538.125 E** |
| --- | --- | --- | --- |
|  |  |  | **412969.406 N** |
|  | | | |

| **Station** | **F14** | **Coordinate** | **512717.313 E** |
| --- | --- | --- | --- |
|  |  |  | **413087.500 N** |
|  | | | |

| **Station** | **F15** | **Coordinate** | **514536.563 E** |
| --- | --- | --- | --- |
|  |  |  | **412968.875 N** |
|  | | | |

| **Station** | **F16** | **Coordinate** | **516537.375 E** |
| --- | --- | --- | --- |
|  |  |  | **412967.969 N** |
|  | | | |

| **Station** | **F17** | **Coordinate** | **510937.47 E** |
| --- | --- | --- | --- |
|  |  |  | **410969.25 N** |
|  | | | |

| **Station** | **F18** | **Coordinate** | **512733.25 E** |
| --- | --- | --- | --- |
|  |  |  | **411250.438 N** |
|  | | | |

| **Station** | **F19** | **Coordinate** | **515037.2 E** |
| --- | --- | --- | --- |
|  |  |  | **410969 N** |
|  | | | |

| **Station** | **F20** | **Coordinate** | **517136.19 E** |
| --- | --- | --- | --- |
|  |  |  | **410969.81 N** |
|  | | | |
