# Supplementary material for: Data for the subsurface characterization of Pahang River Basin with the application of Transient Electromagnetic geophysical surveys
Source: Data Brief. 2020 Apr 23;30:105491. doi: 10.1016/j.dib.2020.105491 (PMC7191212; doi:10.1016/j.dib.2020.105491)
Supplement: Supplementary file 8 [file mmc8.docx]

| **Station** | **G1** | **Coordinate** | 510988.313 E |
| --- | --- | --- | --- |
|  |  |  | 408993.938 N |
|  | | | |

| **Station** | **G2** | **Coordinate** | 514299.688 E |
| --- | --- | --- | --- |
|  |  |  | 409000.344 N |
|  | | | |

| **Station** | **G3** | **Coordinate** | 517899.781 E |
| --- | --- | --- | --- |
|  |  |  | 408999.094 N |
|  | | | |

| **Station** | **G4** | **Coordinate** | 511348.250 E |
| --- | --- | --- | --- |
|  |  |  | 406988.750 N |
|  | | | |

| **Station** | **G5** | **Coordinate** | 514699.1 E |
| --- | --- | --- | --- |
|  |  |  | 407398.6 N |
|  | | | |

| **Station** | **G6** | **Coordinate** | 517399.2 E |
| --- | --- | --- | --- |
|  |  |  | 406998.8 N |
|  | | | |

| **Station** | **G7** | **Coordinate** | 519600.000 E |
| --- | --- | --- | --- |
|  |  |  | 406999.469 N |
|  | | | |

| **Station** | **G8** | **Coordinate** | 511176.844 E |
| --- | --- | --- | --- |
|  |  |  | 404905.063 N |
|  | | | |

| **Station** | **G9** | **Coordinate** | 514005.313 E |
| --- | --- | --- | --- |
|  |  |  | 404976.625 N |
|  | | | |

| **Station** | **G10** | **Coordinate** | 517400.125 E |
| --- | --- | --- | --- |
|  |  |  | 404999.594 N |
|  | | | |

| **Station** | **G12** | **Coordinate** | 522299.813 E |
| --- | --- | --- | --- |
|  |  |  | 404998.563 N |
|  | | | |

| **Station** | **G13** | **Coordinate** | 511135.250 E |
| --- | --- | --- | --- |
|  |  |  | 402992.563 N |
|  | | | |

| **Station** | **G14** | **Coordinate** | 513510.094 E |
| --- | --- | --- | --- |
|  |  |  | 402939.469 N |
|  | | | |

| **Station** | **G15** | **Coordinate** | 515918.460 E |
| --- | --- | --- | --- |
|  |  |  | 402989.531 N |
|  | | | |

| **Station** | **G16** | **Coordinate** | 517674.960 E |
| --- | --- | --- | --- |
|  |  |  | 402885.813 N |
|  | | | |

| **Station** | **G17** | **Coordinate** | 519959.375 E |
| --- | --- | --- | --- |
|  |  |  | 403065.125 N |
|  | | | |

| **Station** | **G18** | **Coordinate** | 522595.281 E |
| --- | --- | --- | --- |
|  |  |  | 403061.656 N |
|  | | | |

| **Station** | **G21** | **Coordinate** | 516198.9 E |
| --- | --- | --- | --- |
|  |  |  | 400999.2 N |
|  | | | |

| **Station** | **G22** | **Coordinate** | 518399.8 E |
| --- | --- | --- | --- |
|  |  |  | 400999.9 N |
|  | | | |

| **Station** | **G23** | **Coordinate** | 519641.875 E |
| --- | --- | --- | --- |
|  |  |  | 400954.000 N |
|  | | | |

| **Station** | **G24** | **Coordinate** | 522200.156 E |
| --- | --- | --- | --- |
|  |  |  | 401000.344 N |
|  | | | |
