# Supplementary material for: Data for the subsurface characterization of Pahang River Basin with the application of Transient Electromagnetic geophysical surveys
Source: Data Brief. 2020 Apr 23;30:105491. doi: 10.1016/j.dib.2020.105491 (PMC7191212; doi:10.1016/j.dib.2020.105491)
Supplement: Supplementary file 9 [file mmc9.docx]

| **Station** | **H1** | **Coordinate** | 511888.500 E |
| --- | --- | --- | --- |
|  |  |  | 398969.188 N |
|  | | | |

| **Station** | **H2** | **Coordinate** | 514087.7 E |
| --- | --- | --- | --- |
|  |  |  | 398969.8 N |
|  | | | |

| **Station** | **H3** | **Coordinate** | 516036.9 E |
| --- | --- | --- | --- |
|  |  |  | 398968.9 N |
|  | | | |

| **Station** | **H4** | **Coordinate** | 519861.281 E |
| --- | --- | --- | --- |
|  |  |  | 398915.844 N |
|  | | | |

| **Station** | **H6** | **Coordinate** | 511737.563 E |
| --- | --- | --- | --- |
|  |  |  | 396970.250 N |
|  | | | |

| **Station** | **H7** | **Coordinate** | 514086.719 E |
| --- | --- | --- | --- |
|  |  |  | 396970.625 N |
|  | | | |

| **Station** | **H8** | **Coordinate** | 515736.656 E |
| --- | --- | --- | --- |
|  |  |  | 397370.031 N |
|  | | | |

| **Station** | **H9** | **Coordinate** | 520784.281 E |
| --- | --- | --- | --- |
|  |  |  | 396994.688 N |
|  | | | |

| **Station** | **H10** | **Coordinate** | 510738.500 E |
| --- | --- | --- | --- |
|  |  |  | 394769.844 N |
|  | | | |

| **Station** | **H11** | **Coordinate** | 514037.625 E |
| --- | --- | --- | --- |
|  |  |  | 394969.688 N |
|  | | | |

| **Station** | **H12** | **Coordinate** | 517236.531 E |
| --- | --- | --- | --- |
|  |  |  | 394970.813 N |
|  | | | |

| **Station** | **H13** | **Coordinate** | 520981.375 E |
| --- | --- | --- | --- |
|  |  |  | 394869.969 N |
|  | | | |

| **Station** | **H14** | **Coordinate** | 510937.000 E |
| --- | --- | --- | --- |
|  |  |  | 392470.063 N |
|  | | | |

| **Station** | **H15** | **Coordinate** | 514437.594 E |
| --- | --- | --- | --- |
|  |  |  | 392371.156 N |
|  | | | |

| **Station** | **H16** | **Coordinate** | 517636.750 E |
| --- | --- | --- | --- |
|  |  |  | 392519.688 N |
|  | | | |

| **Station** | **H17** | **Coordinate** | 521364.969 E |
| --- | --- | --- | --- |
|  |  |  | 392359.906 N |
|  | | | |
