# Supplementary material for: Data for the subsurface characterization of Pahang River Basin with the application of Transient Electromagnetic geophysical surveys
Source: Data Brief. 2020 Apr 23;30:105491. doi: 10.1016/j.dib.2020.105491 (PMC7191212; doi:10.1016/j.dib.2020.105491)
Supplement: Supplementary file 18 [file mmc18.docx]

| **Station** | **A1** | **Coordinate** |  |
| --- | --- | --- | --- |
|  |  |  |  |
| **Sounding Curve** | | | |
| **Average Decay**  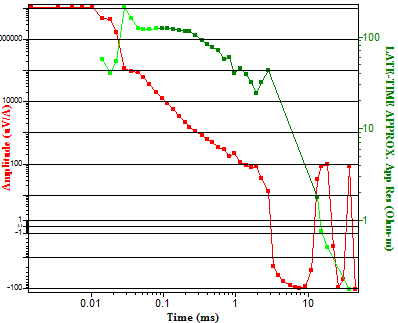 | | | |
| **First Decay**  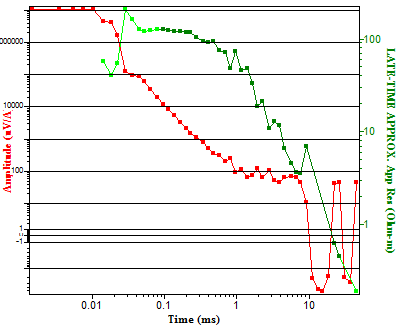 | | | |

| **Station** | **A2** | **Coordinate** |  |
| --- | --- | --- | --- |
|  |  |  |  |
| **Sounding Curve** | | | |
| **Average Decay**  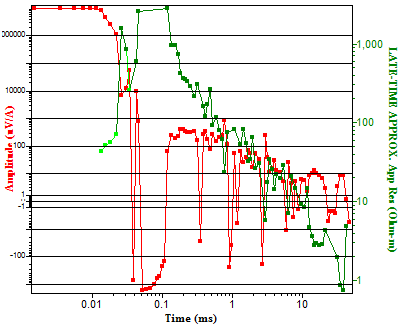 | | | |
| **First Decay**  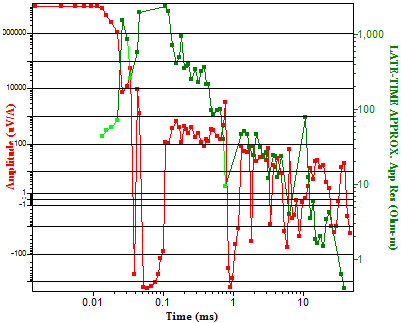 | | | |

| **Station** | **A3** | **Coordinate** |  |
| --- | --- | --- | --- |
|  |  |  |  |
| **Sounding Curve** | | | |
| **Average Decay**  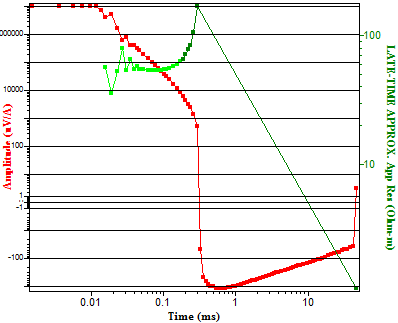 | | | |
| **First Decay**  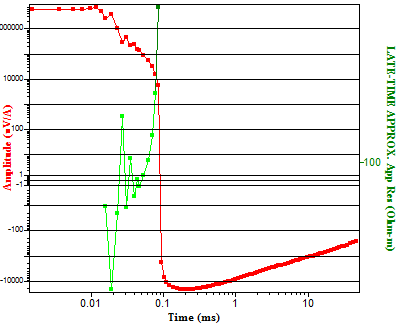 | | | |

| **Station** | **A4** | **Coordinate** |  |
| --- | --- | --- | --- |
|  |  |  |  |
| **Sounding Curve** | | | |
| **Average Decay**  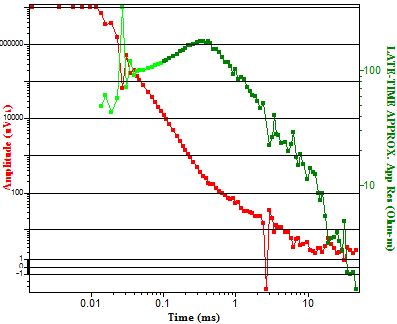 | | | |
| **First Decay**  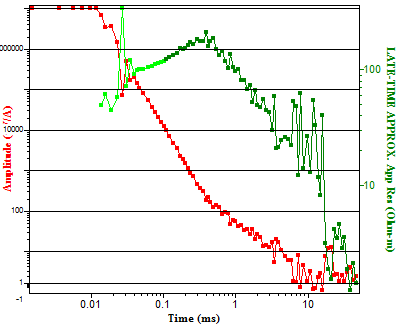 | | | |

| **Station** | **A5** | **Coordinate** |  |
| --- | --- | --- | --- |
|  |  |  |  |
| **Sounding Curve** | | | |
| **Average Decay**  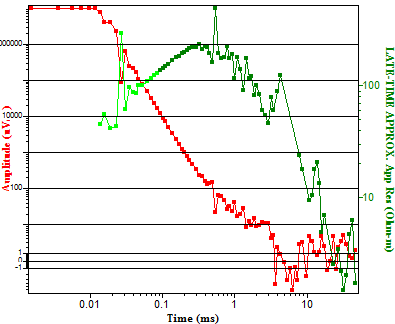 | | | |
| **First Decay**  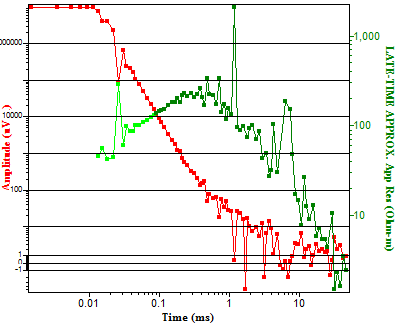 | | | |

| **Station** | **A6** | **Coordinate** |  |
| --- | --- | --- | --- |
|  |  |  |  |
| **Sounding Curve** | | | |
| **Average Decay**  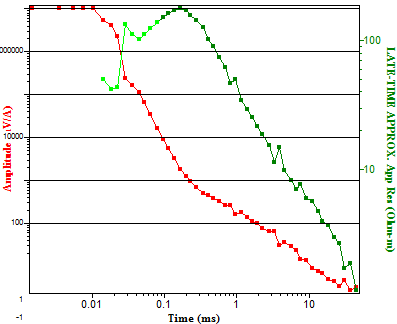 | | | |
| **First Decay**  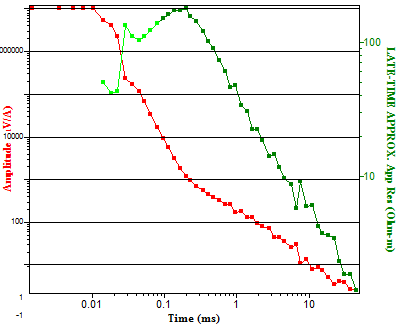 | | | |

| **Station** | **A7** | **Coordinate** |  |
| --- | --- | --- | --- |
|  |  |  |  |
| **Sounding Curve** | | | |
| **Average Decay**  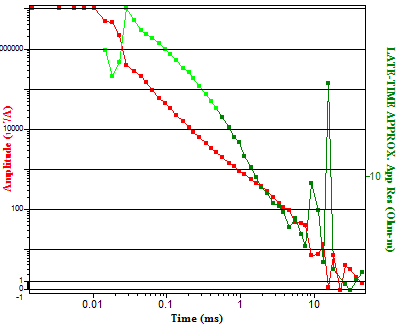 | | | |
| **First Decay**  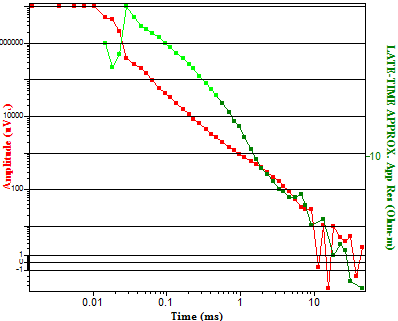 | | | |

| **Station** | **A8** | **Coordinate** |  |
| --- | --- | --- | --- |
|  |  |  |  |
| **Sounding Curve** | | | |
| **Average Decay**  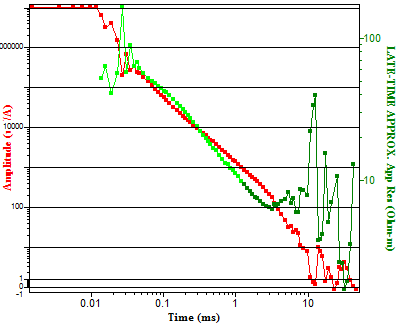 | | | |
| **First Decay**  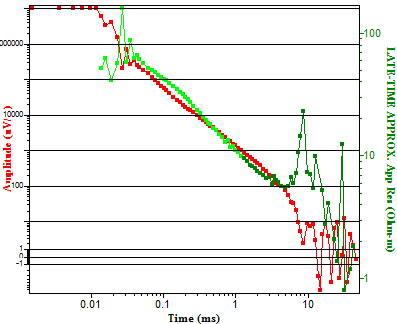 | | | |

| **Station** | **A9** | **Coordinate** |  |
| --- | --- | --- | --- |
|  |  |  |  |
| **Sounding Curve** | | | |
| **Average Decay**  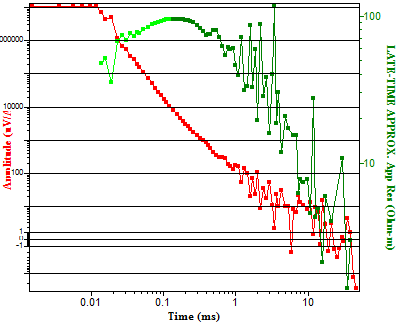 | | | |
| **First Decay**  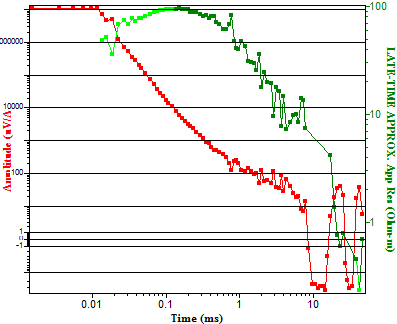 | | | |

| **Station** | **A10** | **Coordinate** |  |
| --- | --- | --- | --- |
|  |  |  |  |
| **Sounding Curve** | | | |
| **Average Decay**  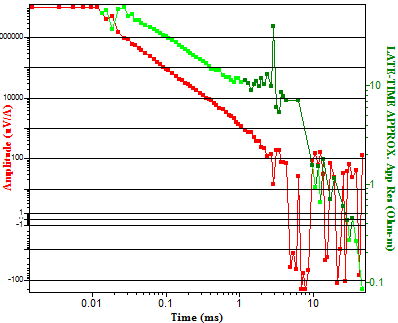 | | | |
| **First Decay**  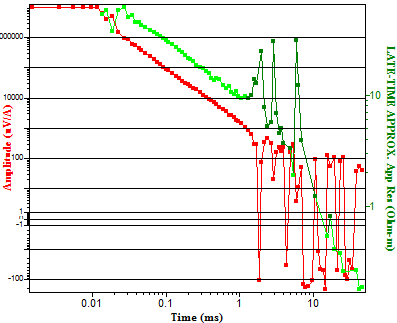 | | | |

| **Station** | **A11** | **Coordinate** |  |
| --- | --- | --- | --- |
|  |  |  |  |
| **Sounding Curve** | | | |
| **Average Decay**  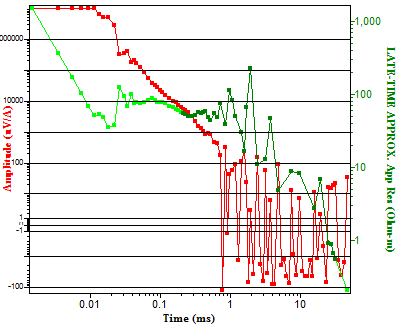 | | | |
| **First Decay**  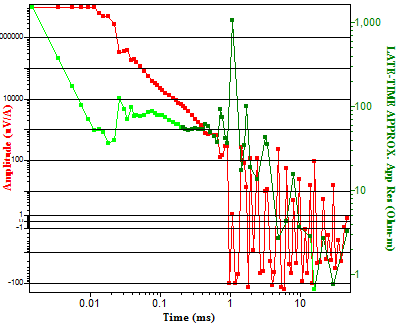 | | | |

| **Station** | **A12** | **Coordinate** |  |
| --- | --- | --- | --- |
|  |  |  |  |
| **Sounding Curve** | | | |
| **Average Decay**  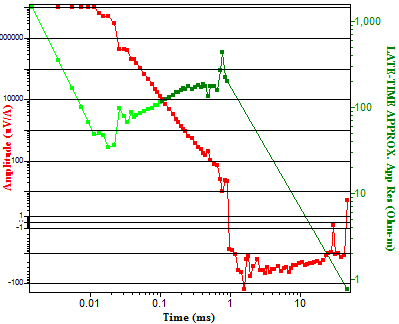 | | | |
| **First Decay**  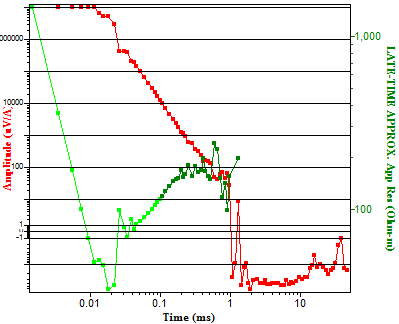 | | | |

| **Station** | **A13** | **Coordinate** |  |
| --- | --- | --- | --- |
|  |  |  |  |
| **Sounding Curve** | | | |
| **Average Decay**  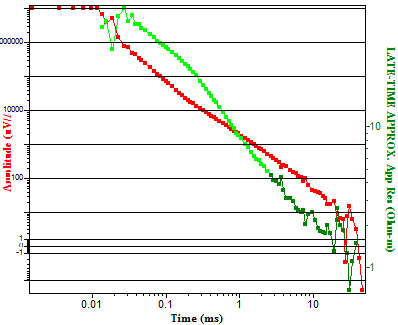 | | | |
| **First Decay**  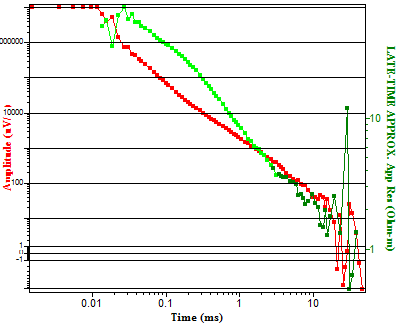 | | | |

| **Station** | **A14** | **Coordinate** |  |
| --- | --- | --- | --- |
|  |  |  |  |
| **Sounding Curve** | | | |
| **Average Decay**  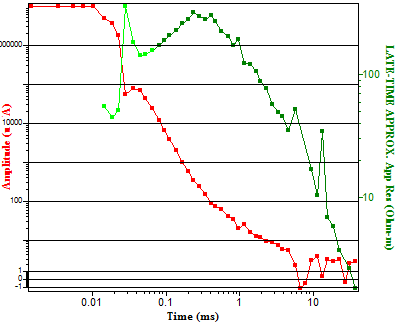 | | | |
| **First Decay**  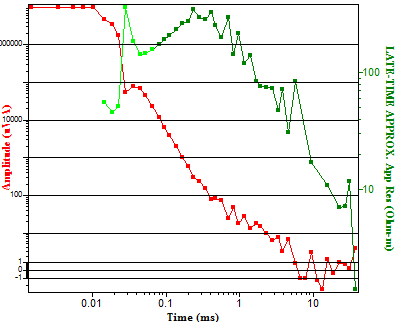 | | | |

| **Station** | **A15** | **Coordinate** |  |
| --- | --- | --- | --- |
|  |  |  |  |
| **Sounding Curve** | | | |
| **Average Decay**  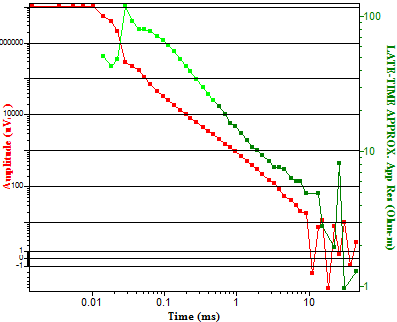 | | | |
| **First Decay**  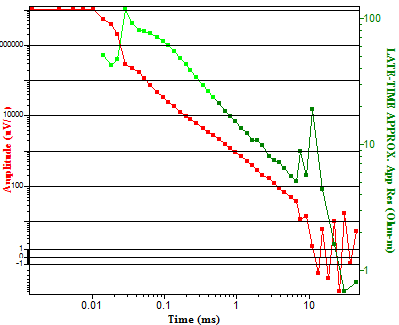 | | | |

| **Station** | **A16** | **Coordinate** |  |
| --- | --- | --- | --- |
|  |  |  |  |
| **Sounding Curve** | | | |
| **Average Decay**  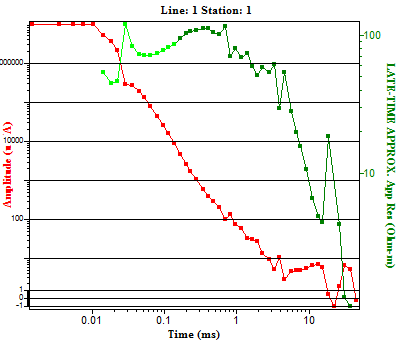 | | | |
| **First Decay**  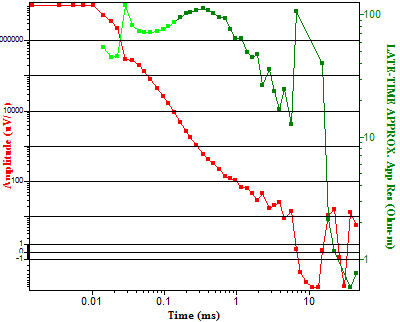 | | | |

| **Station** | **A17** | **Coordinate** |  |
| --- | --- | --- | --- |
|  |  |  |  |
| **Sounding Curve** | | | |
| **Average Decay**  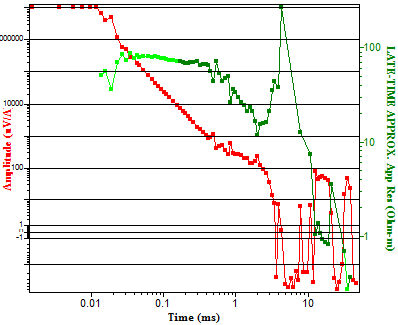 | | | |
| **First Decay**  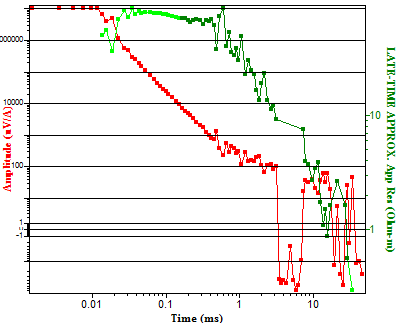 | | | |

| **Station** | **A18** | **Coordinate** |  |
| --- | --- | --- | --- |
|  |  |  |  |
| **Sounding Curve** | | | |
| **Average Decay**  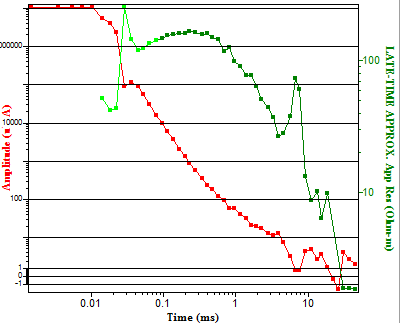 | | | |
| **First Decay**  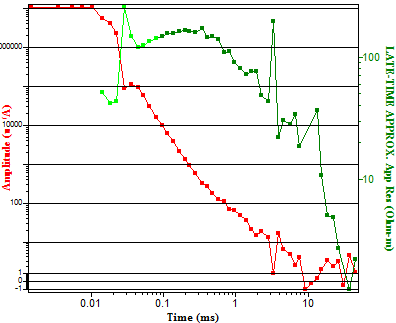 | | | |

| **Station** | **A19** | **Coordinate** |  |
| --- | --- | --- | --- |
|  |  |  |  |
| **Sounding Curve** | | | |
| **Average Decay**  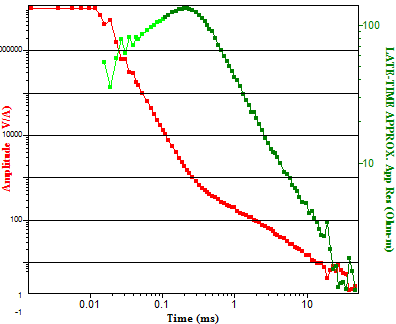 | | | |
| **First Decay**  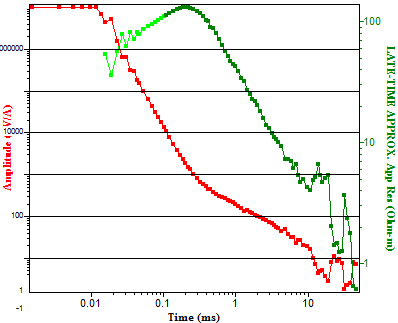 | | | |

| **Station** | **A20** | **Coordinate** |  |
| --- | --- | --- | --- |
|  |  |  |  |
| **Sounding Curve** | | | |
| **Average Decay**  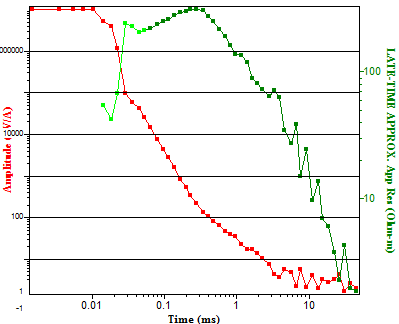 | | | |
| **First Decay**  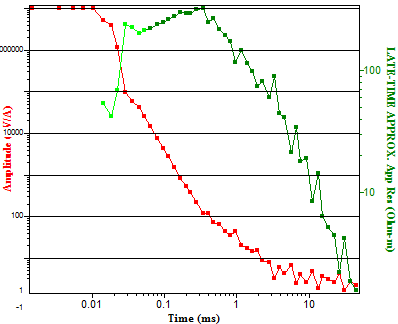 | | | |

| **Station** | **A21** | **Coordinate** |  |
| --- | --- | --- | --- |
|  |  |  |  |
| **Sounding Curve** | | | |
| **Average Decay**  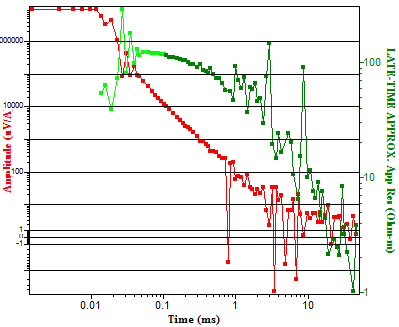 | | | |
| **First Decay**  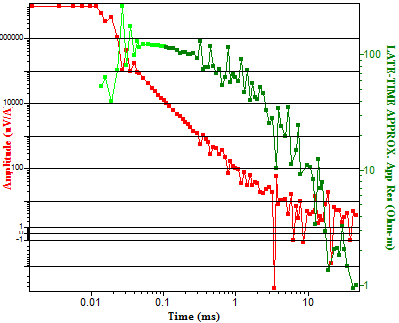 | | | |

| **Station** | **A22** | **Coordinate** |  |
| --- | --- | --- | --- |
|  |  |  |  |
| **Sounding Curve** | | | |
| **Average Decay**  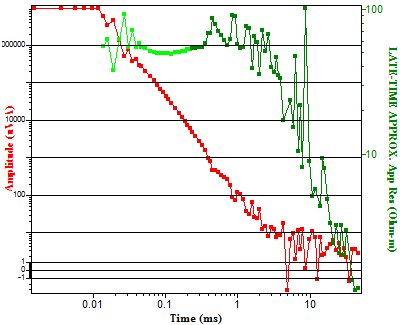 | | | |
| **First Decay**  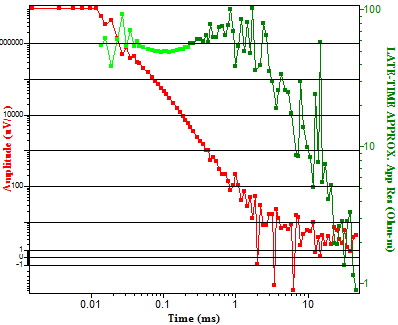 | | | |

| **Station** | **A23** | **Coordinate** |  |
| --- | --- | --- | --- |
|  |  |  |  |
| **Sounding Curve** | | | |
| **Average Decay**  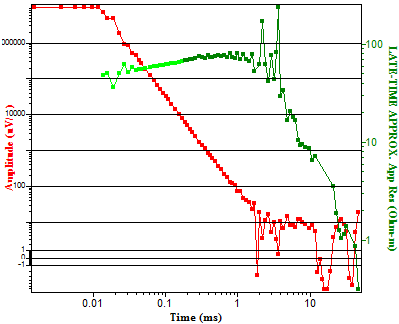 | | | |
| **First Decay**  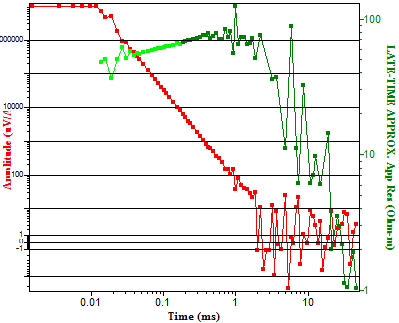 | | | |

| **Station** | **A24** | **Coordinate** |  |
| --- | --- | --- | --- |
|  |  |  |  |
| **Sounding Curve** | | | |
| **Average Decay**  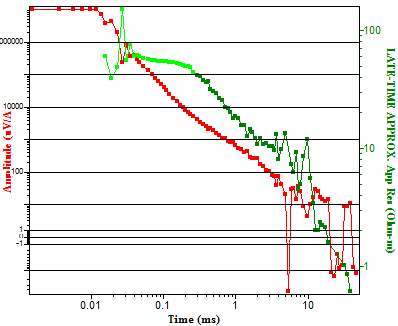 | | | |
| **First Decay**  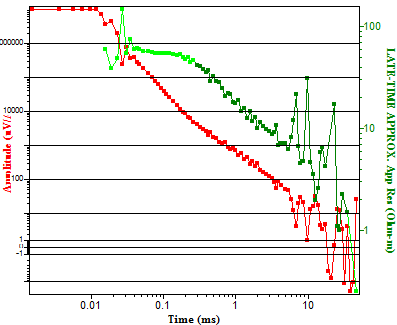 | | | |

| **Station** | **A25** | **Coordinate** |  |
| --- | --- | --- | --- |
|  |  |  |  |
| **Sounding Curve** | | | |
| **Average Decay**  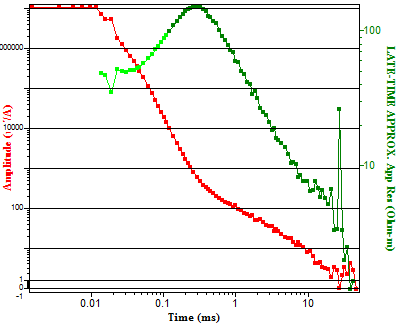 | | | |
| **First Decay**  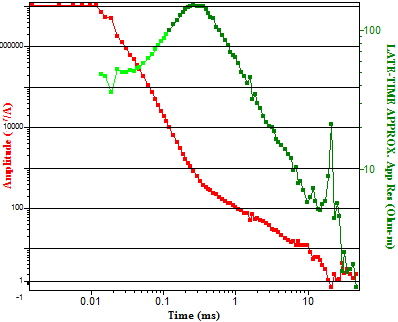 | | | |

| **Station** | **A26** | **Coordinate** |  |
| --- | --- | --- | --- |
|  |  |  |  |
| **Sounding Curve** | | | |
| **Average Decay**  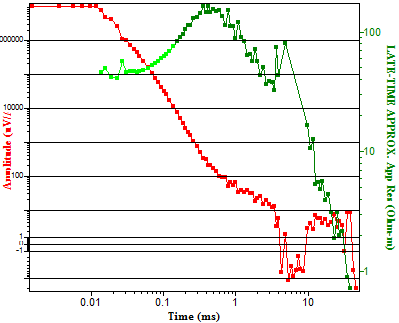 | | | |
| **First Decay**  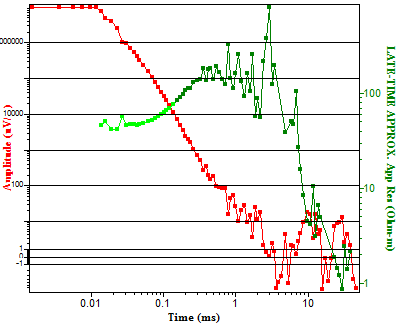 | | | |

| **Station** | **A27** | **Coordinate** |  |
| --- | --- | --- | --- |
|  |  |  |  |
| **Sounding Curve** | | | |
| **Average Decay**  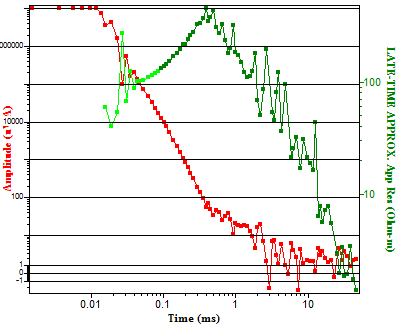 | | | |
| **First Decay**  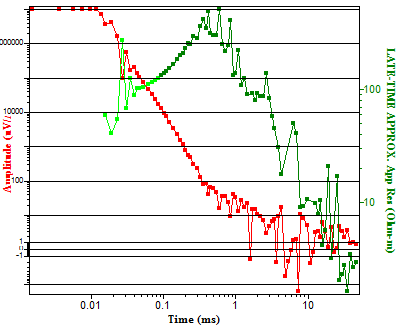 | | | |

| **Station** | **A28** | **Coordinate** |  |
| --- | --- | --- | --- |
|  |  |  |  |
| **Sounding Curve** | | | |
| **Average Decay**  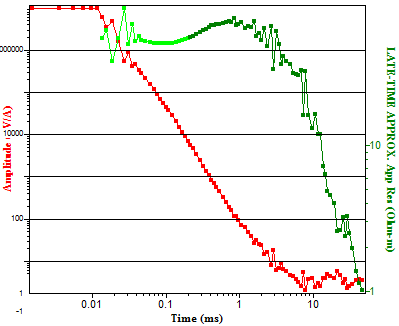 | | | |
| **First Decay**  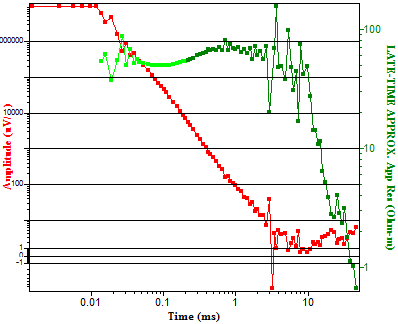 | | | |

| **Station** | **A29** | **Coordinate** |  |
| --- | --- | --- | --- |
|  |  |  |  |
| **Sounding Curve** | | | |
| **Average Decay**  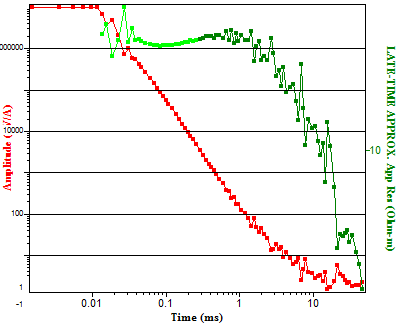 | | | |
| **First Decay**  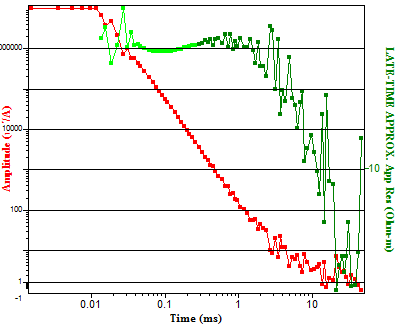 | | | |

| **Station** | **A30** | **Coordinate** |  |
| --- | --- | --- | --- |
|  |  |  |  |
| **Sounding Curve** | | | |
| **Average Decay**  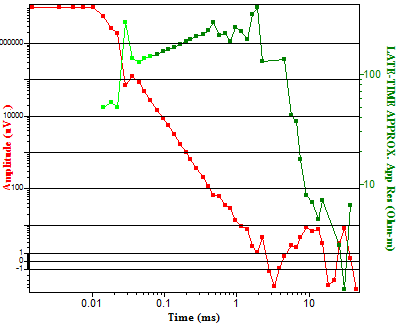 | | | |
| **First Decay**  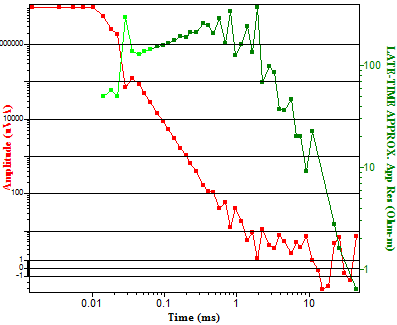 | | | |

| **Station** | **A31** | **Coordinate** |  |
| --- | --- | --- | --- |
|  |  |  |  |
| **Sounding Curve** | | | |
| **Average Decay**  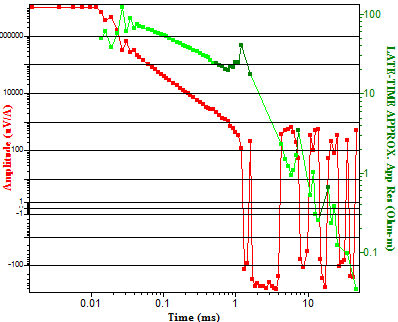 | | | |
| **First Decay**  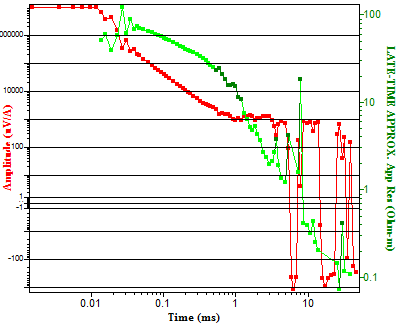 | | | |

| **Station** | **A32** | **Coordinate** |  |
| --- | --- | --- | --- |
|  |  |  |  |
| **Sounding Curve** | | | |
| **Average Decay**  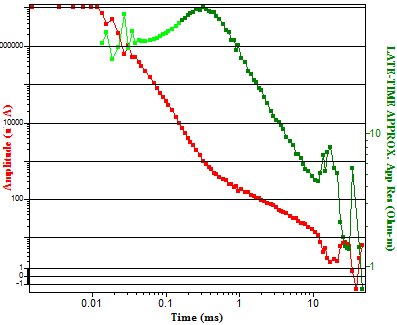 | | | |
| **First Decay**  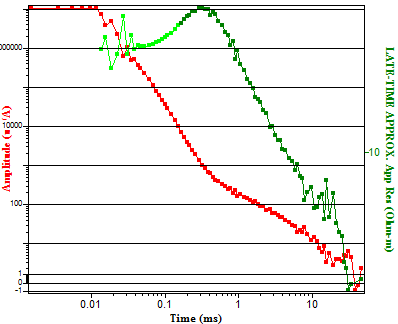 | | | |

| **Station** | **A33** | **Coordinate** |  |
| --- | --- | --- | --- |
|  |  |  |  |
| **Sounding Curve** | | | |
| **Average Decay**  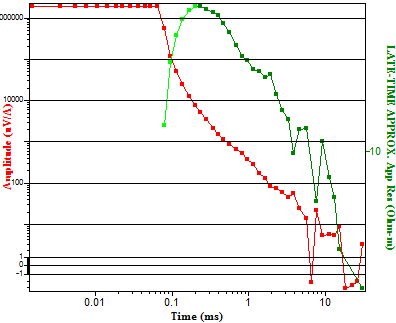 | | | |
| **First Decay**  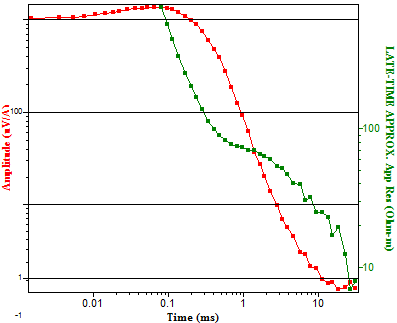 | | | |

| **Station** | **A34** | **Coordinate** |  |
| --- | --- | --- | --- |
|  |  |  |  |
| **Sounding Curve** | | | |
| **First Decay**  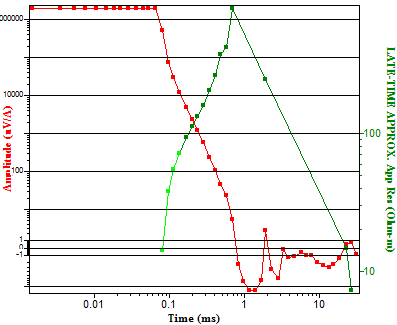 | | | |
|  | | | |

| **Station** | **A35** | **Coordinate** |  |
| --- | --- | --- | --- |
|  |  |  |  |
| **Sounding Curve** | | | |
| **Average Decay**  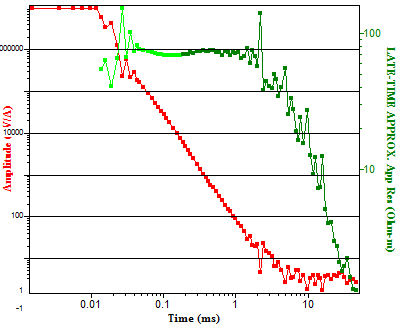 | | | |
| **First Decay**  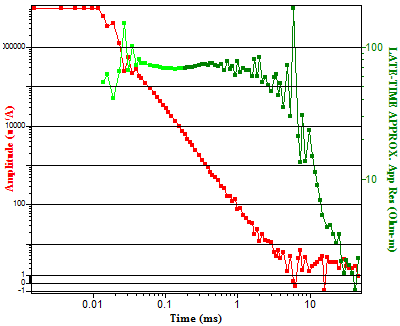 | | | |

| **Station** | **A36** | **Coordinate** |  |
| --- | --- | --- | --- |
|  |  |  |  |
| **Sounding Curve** | | | |
| **Average Decay**  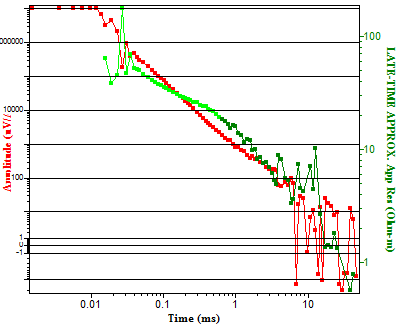 | | | |
| **First Decay**  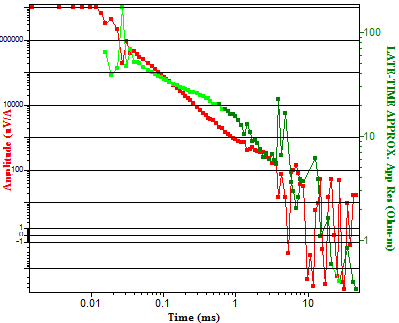 | | | |

| **Station** | **A37** | **Coordinate** |  |
| --- | --- | --- | --- |
|  |  |  |  |
| **Sounding Curve** | | | |
| **Average Decay**  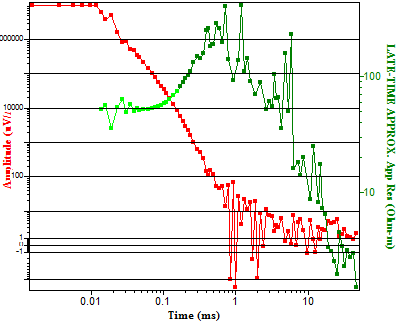 | | | |
| **First Decay**  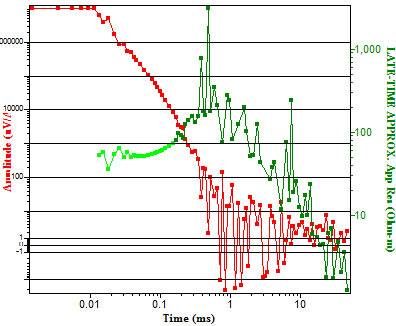 | | | |

| **Station** | **A38** | **Coordinate** |  |
| --- | --- | --- | --- |
|  |  |  |  |
| **Sounding Curve** | | | |
| **Average Decay**  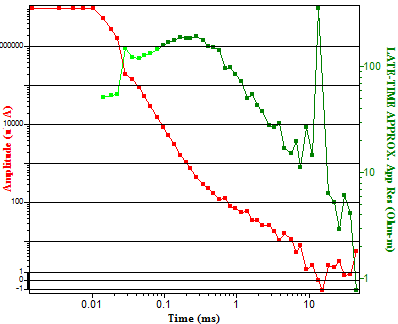 | | | |
| **First Decay**  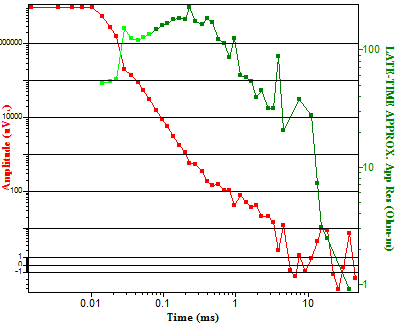 | | | |

| **Station** | **A39** | **Coordinate** |  |
| --- | --- | --- | --- |
|  |  |  |  |
| **Sounding Curve** | | | |
| **Average Decay**  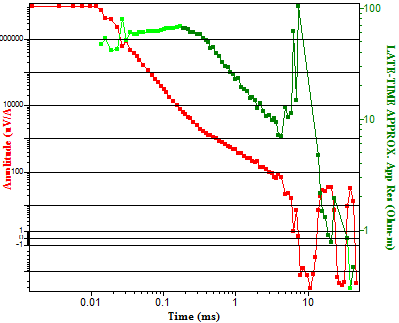 | | | |
| **First Decay**  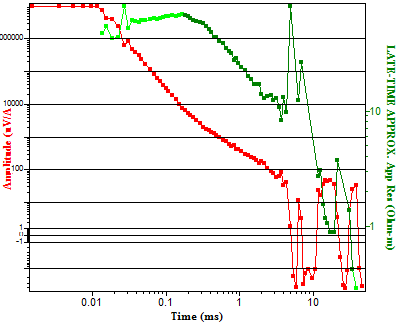 | | | |

| **Station** | **A40** | **Coordinate** |  |
| --- | --- | --- | --- |
|  |  |  |  |
| **Sounding Curve** | | | |
| **Average Decay**  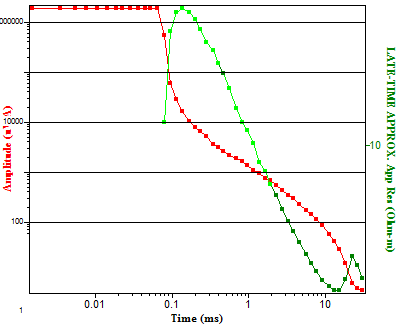 | | | |
| **First Decay**  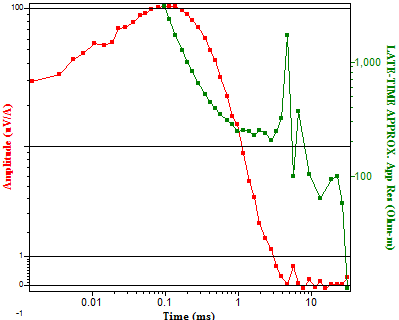 | | | |
