# Supplementary material for: Data for the subsurface characterization of Pahang River Basin with the application of Transient Electromagnetic geophysical surveys
Source: Data Brief. 2020 Apr 23;30:105491. doi: 10.1016/j.dib.2020.105491 (PMC7191212; doi:10.1016/j.dib.2020.105491)
Supplement: Supplementary file 19 [file mmc19.docx]

| **Station** | **B1** | **Coordinate** |  |
| --- | --- | --- | --- |
|  |  |  |  |
| **Sounding Curve** | | | |
| **Average Decay**  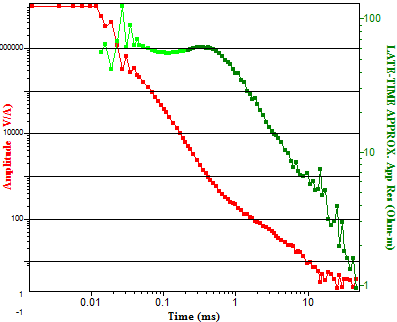 | | | |
| **First Decay**  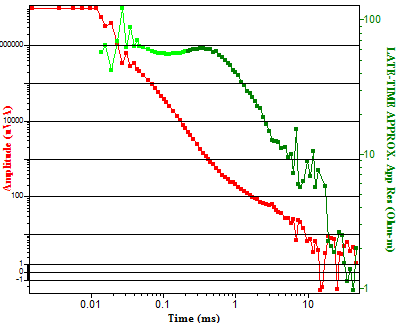 | | | |

| **Station** | **B2** | **Coordinate** |  |
| --- | --- | --- | --- |
|  |  |  |  |
| **Sounding Curve** | | | |
| **Average Decay**  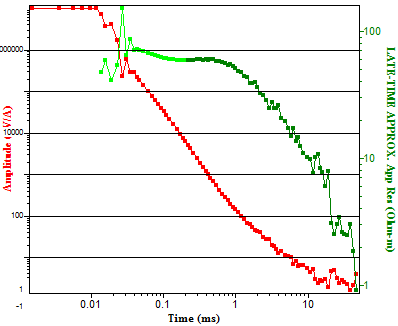 | | | |
| **First Decay**  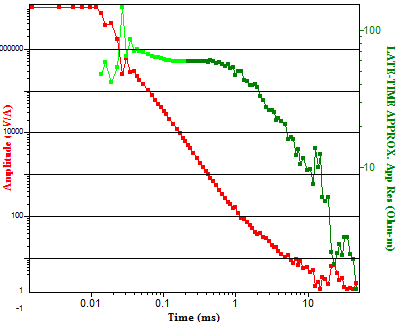 | | | |

| **Station** | **B3** | **Coordinate** |  |
| --- | --- | --- | --- |
|  |  |  |  |
| **Sounding Curve** | | | |
| **Average Decay**  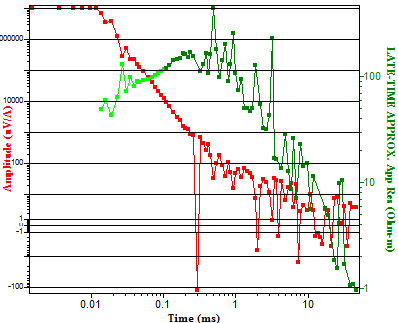 | | | |
| **First Decay**  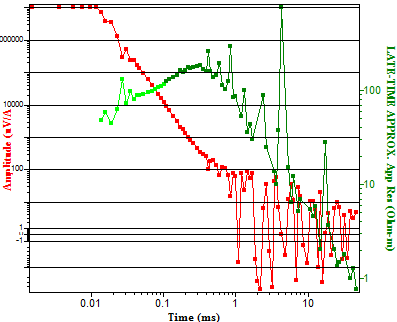 | | | |

| **Station** | **B4** | **Coordinate** |  |
| --- | --- | --- | --- |
|  |  |  |  |
| **Sounding Curve** | | | |
| **Average Decay**  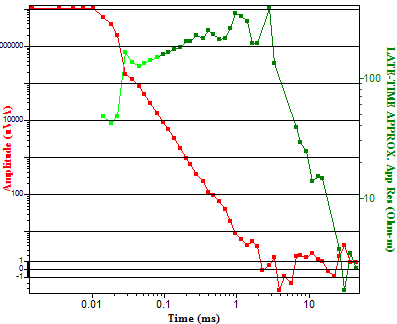 | | | |
| **First Decay**  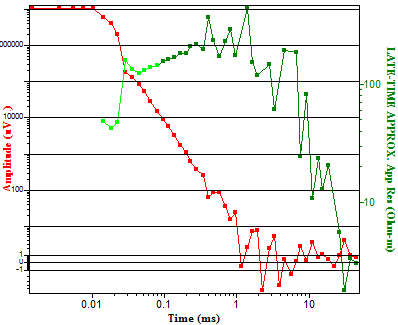 | | | |

| **Station** | **B5** | **Coordinate** |  |
| --- | --- | --- | --- |
|  |  |  |  |
| **Sounding Curve** | | | |
| **Average Decay**  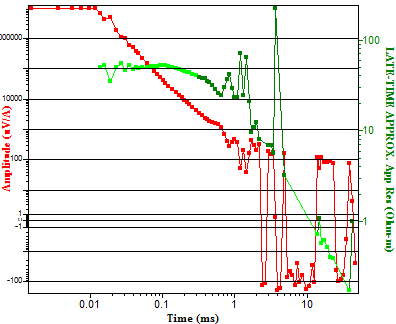 | | | |
| **First Decay**  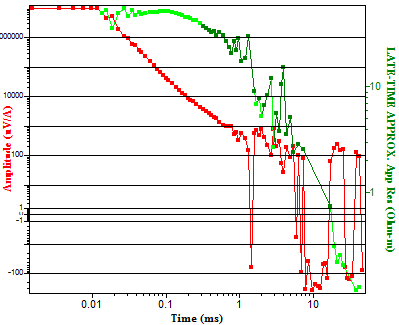 | | | |

| **Station** | **B6** | **Coordinate** |  |
| --- | --- | --- | --- |
|  |  |  |  |
| **Sounding Curve** | | | |
| **Average Decay**  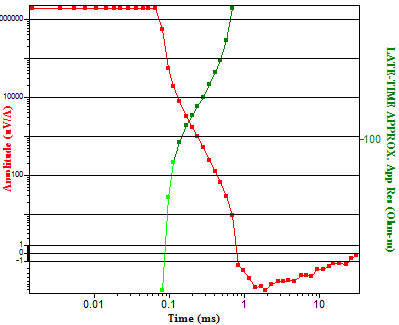 | | | |
| **First Decay**  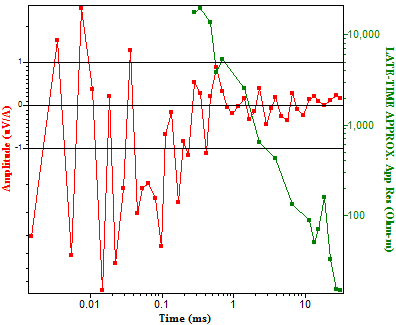 | | | |

| **Station** | **B7** | **Coordinate** |  |
| --- | --- | --- | --- |
|  |  |  |  |
| **Sounding Curve** | | | |
| **Average Decay**  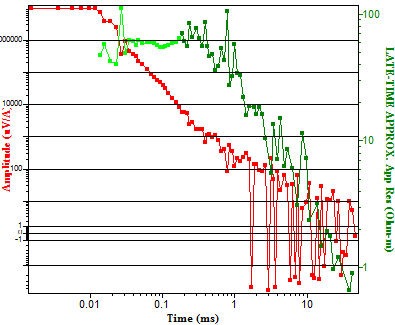 | | | |
| **First Decay**  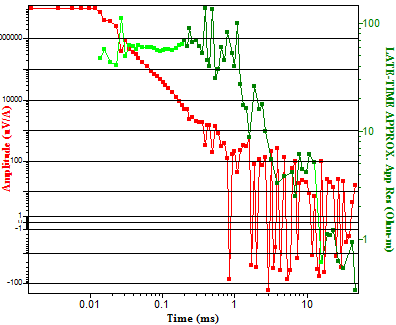 | | | |

| **Station** | **B8** | **Coordinate** |  |
| --- | --- | --- | --- |
|  |  |  |  |
| **Sounding Curve** | | | |
| **Average Decay**  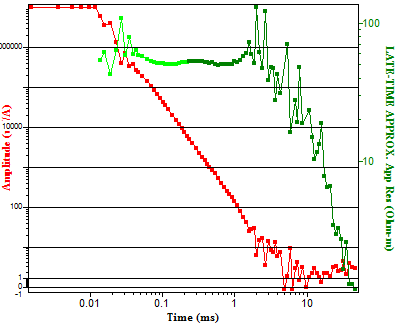 | | | |
| **First Decay**  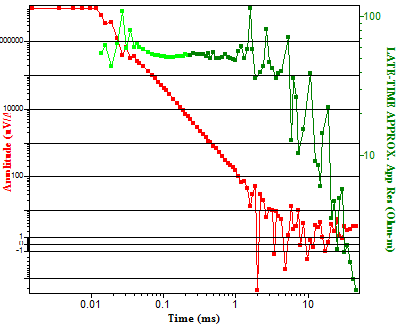 | | | |

| **Station** | **B9** | **Coordinate** |  |
| --- | --- | --- | --- |
|  |  |  |  |
| **Sounding Curve** | | | |
| **Average Decay**  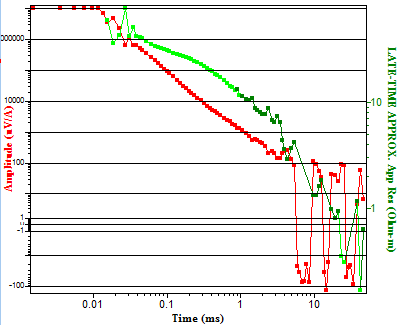 | | | |
| **First Decay**  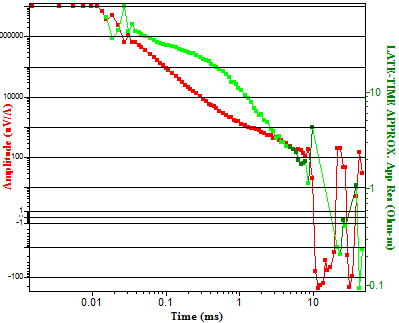 | | | |

| **Station** | **B10** | **Coordinate** |  |
| --- | --- | --- | --- |
|  |  |  |  |
| **Sounding Curve** | | | |
| **Average Decay**  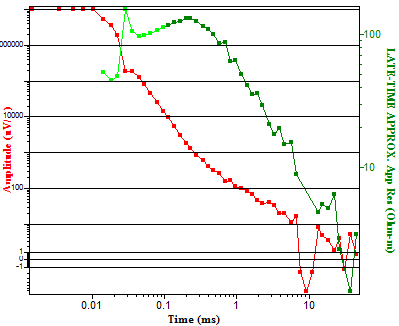 | | | |
| **First Decay**  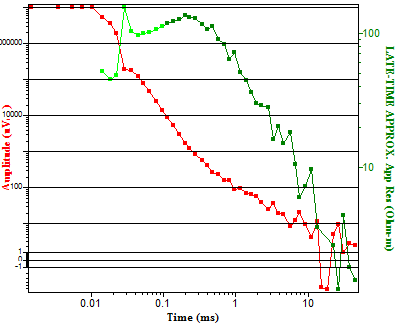 | | | |

| **Station** | **B11** | **Coordinate** |  |
| --- | --- | --- | --- |
|  |  |  |  |
| **Sounding Curve** | | | |
| **Average Decay**  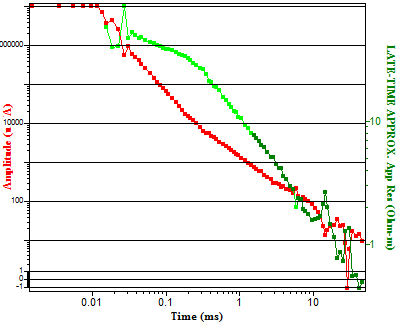 | | | |
| **First Decay**  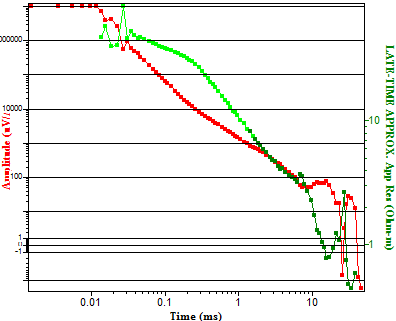 | | | |

| **Station** | **B12** | **Coordinate** |  |
| --- | --- | --- | --- |
|  |  |  |  |
| **Sounding Curve** | | | |
| **Average Decay**  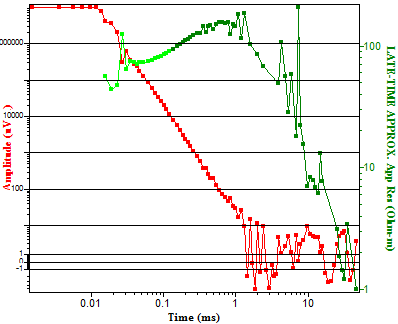 | | | |
| **First Decay**  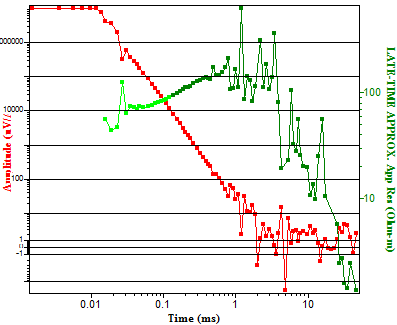 | | | |

| **Station** | **B13** | **Coordinate** |  |
| --- | --- | --- | --- |
|  |  |  |  |
| **Sounding Curve** | | | |
| **Average Decay**  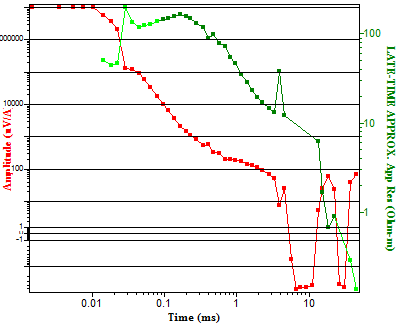 | | | |
| **First Decay**  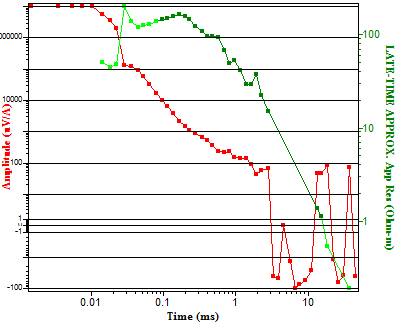 | | | |

| **Station** | **B14** | **Coordinate** |  |
| --- | --- | --- | --- |
|  |  |  |  |
| **Sounding Curve** | | | |
| **Average Decay**  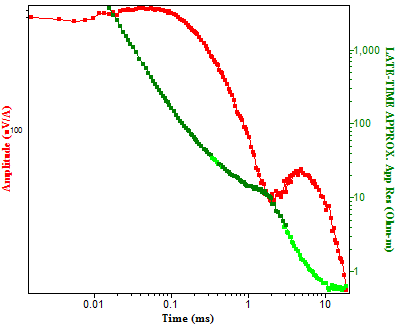 | | | |
| **First Decay**  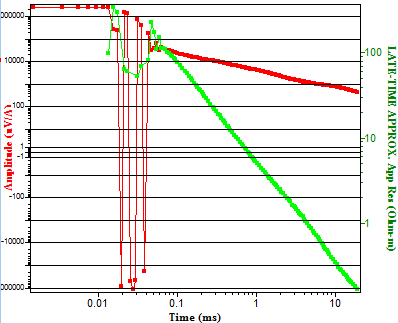 | | | |

| **Station** | **B15** | **Coordinate** |  |
| --- | --- | --- | --- |
|  |  |  |  |
| **Sounding Curve** | | | |
| **Average Decay**  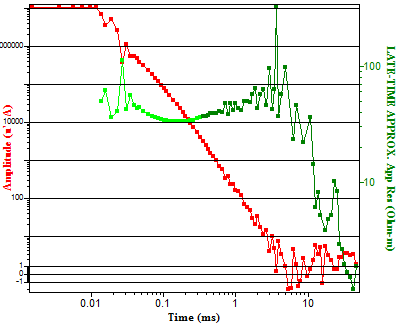 | | | |
| **First Decay**  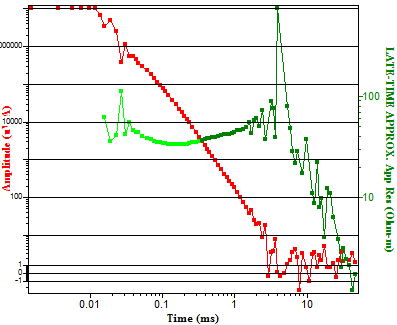 | | | |

| **Station** | **B16** | **Coordinate** |  |
| --- | --- | --- | --- |
|  |  |  |  |
| **Sounding Curve** | | | |
| **Average Decay**  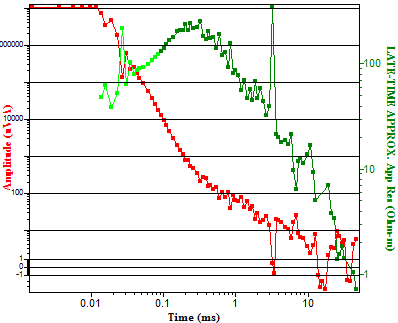 | | | |
| **First Decay**  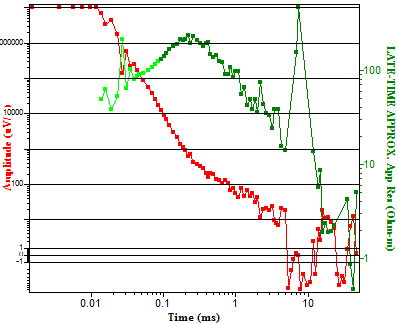 | | | |

| **Station** | **B17** | **Coordinate** |  |
| --- | --- | --- | --- |
|  |  |  |  |
| **Sounding Curve** | | | |
| **Average Decay**  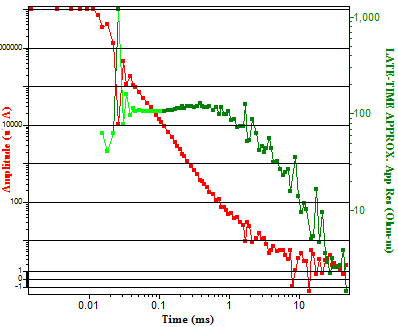 | | | |
| **First Decay**  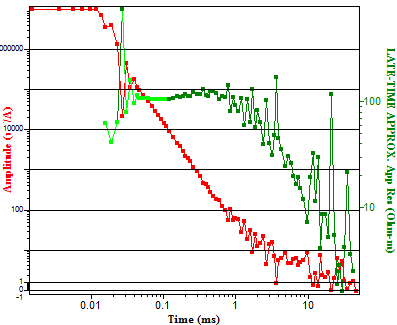 | | | |

| **Station** | **B19** | **Coordinate** |  |
| --- | --- | --- | --- |
|  |  |  |  |
| **Sounding Curve** | | | |
| **Average Decay**  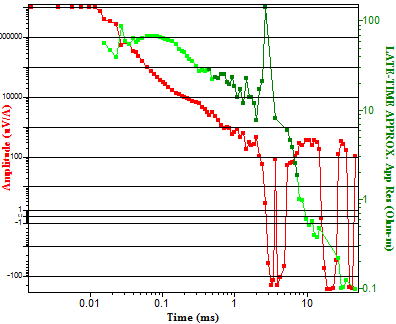 | | | |
| **First Decay**  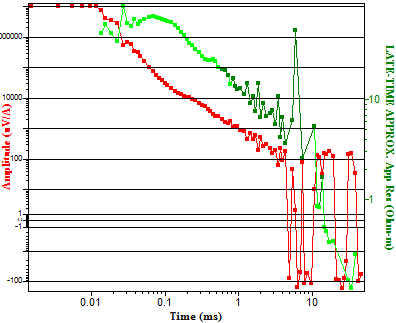 | | | |

| **Station** | **B20** | **Coordinate** |  |
| --- | --- | --- | --- |
|  |  |  |  |
| **Sounding Curve** | | | |
| **First Decay**  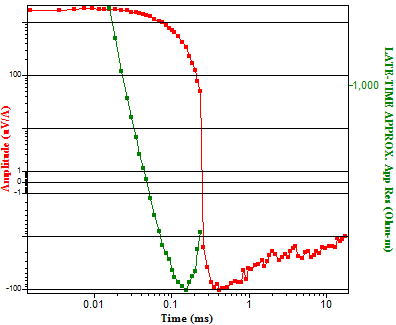 | | | |
|  | | | |

| **Station** | **B21** | **Coordinate** |  |
| --- | --- | --- | --- |
|  |  |  |  |
| **Sounding Curve** | | | |
| **Average Decay**  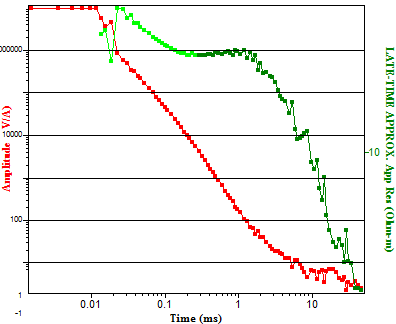 | | | |
| **First Decay**  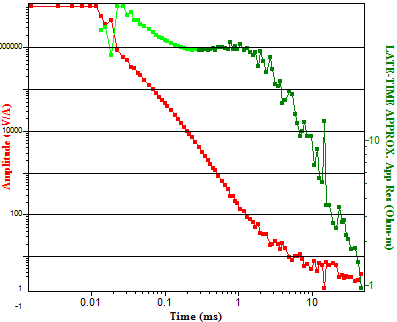 | | | |

| **Station** | **B22** | **Coordinate** |  |
| --- | --- | --- | --- |
|  |  |  |  |
| **Sounding Curve** | | | |
| **Average Decay**  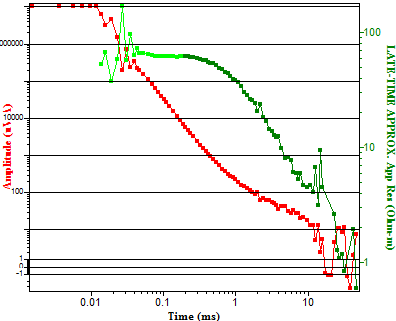 | | | |
| **First Decay**  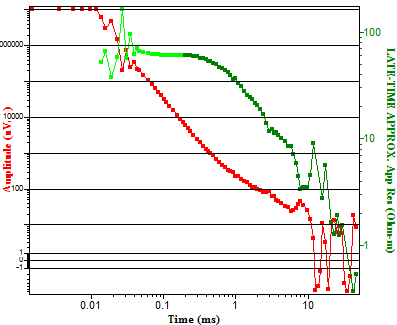 | | | |

| **Station** | **B23** | **Coordinate** |  |
| --- | --- | --- | --- |
|  |  |  |  |
| **Sounding Curve** | | | |
| **Average Decay**  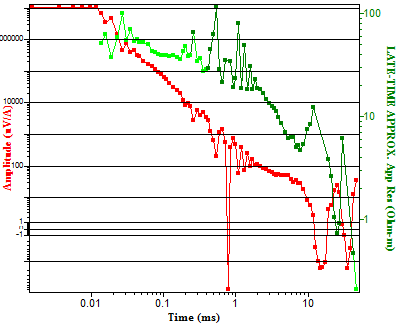 | | | |
| **First Decay**  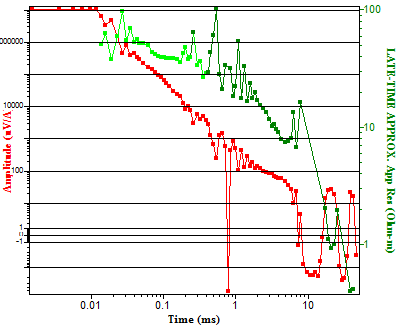 | | | |

| **Station** | **B24** | **Coordinate** |  |
| --- | --- | --- | --- |
|  |  |  |  |
| **Sounding Curve** | | | |
| **Average Decay**  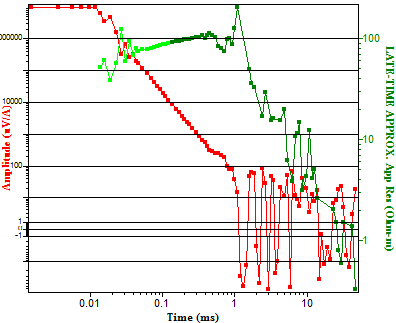 | | | |
| **First Decay**  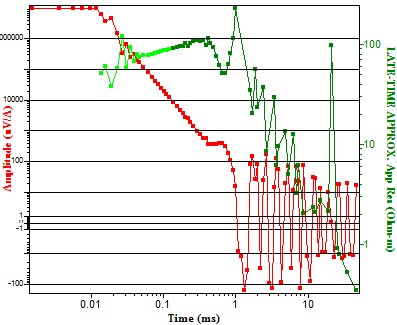 | | | |

| **Station** | **B25** | **Coordinate** |  |
| --- | --- | --- | --- |
|  |  |  |  |
| **Sounding Curve** | | | |
| **Average Decay**  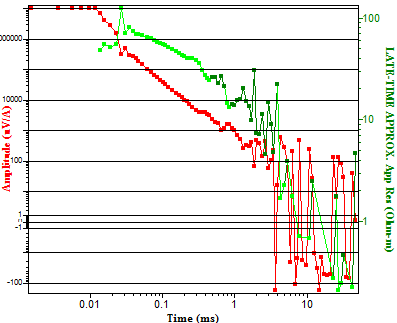 | | | |
| **First Decay**  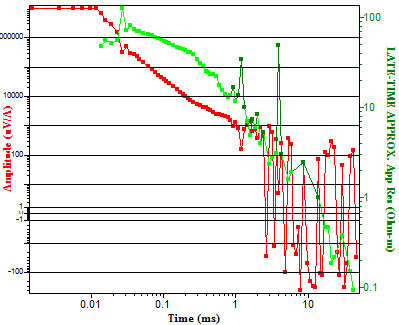 | | | |

| **Station** | **B26** | **Coordinate** |  |
| --- | --- | --- | --- |
|  |  |  |  |
| **Sounding Curve** | | | |
| **Average Decay**  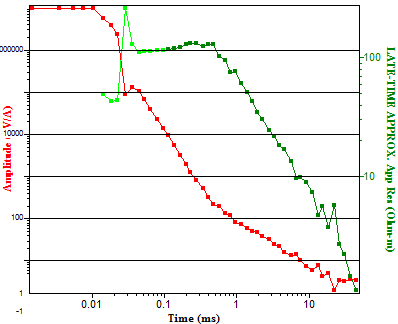 | | | |
| **First Decay**  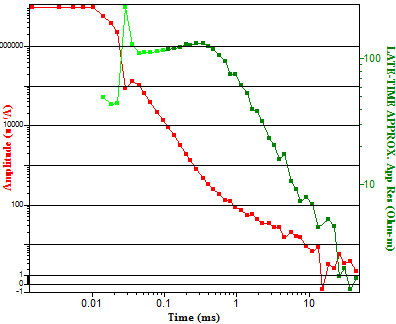 | | | |

| **Station** | **B27** | **Coordinate** |  |
| --- | --- | --- | --- |
|  |  |  |  |
| **Sounding Curve** | | | |
| **Average Decay**  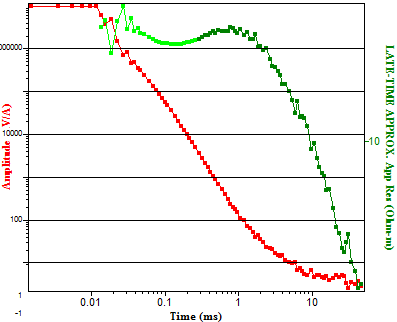 | | | |
| **First Decay**  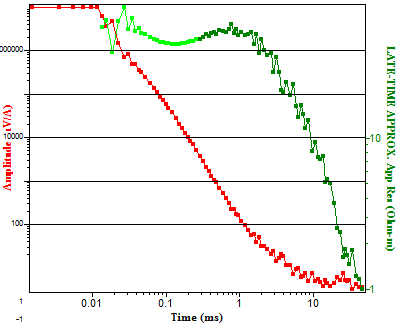 | | | |

| **Station** | **B28** | **Coordinate** |  |
| --- | --- | --- | --- |
|  |  |  |  |
| **Sounding Curve** | | | |
| **Average Decay**  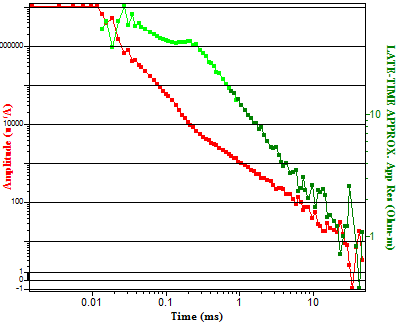 | | | |
| **First Decay**  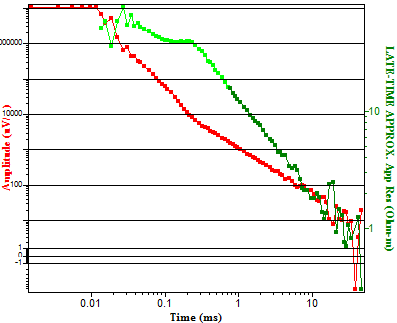 | | | |

| **Station** | **B29** | **Coordinate** |  |
| --- | --- | --- | --- |
|  |  |  |  |
| **Sounding Curve** | | | |
| **Average Decay**  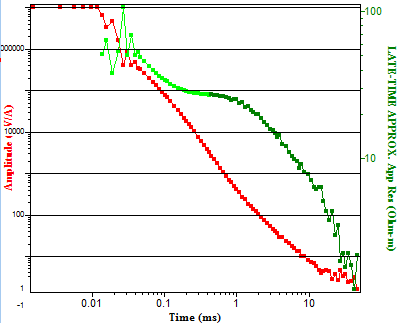 | | | |
| **First Decay**  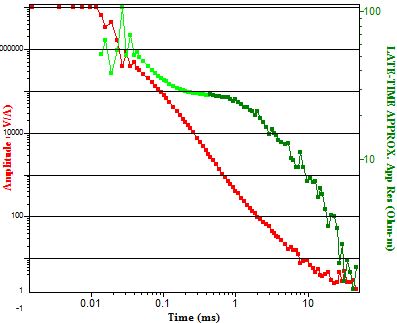 | | | |

| **Station** | **B30** | **Coordinate** |  |
| --- | --- | --- | --- |
|  |  |  |  |
| **Sounding Curve** | | | |
| **Average Decay**  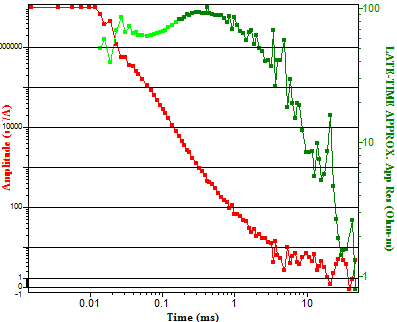 | | | |
| **First Decay**  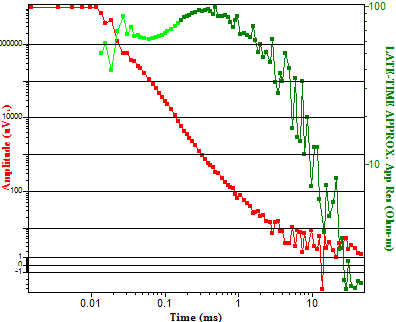 | | | |

| **Station** | **B31** | **Coordinate** |  |
| --- | --- | --- | --- |
|  |  |  |  |
| **Sounding Curve** | | | |
| **Average Decay**  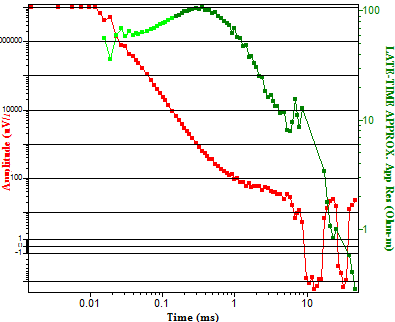 | | | |
| **First Decay**  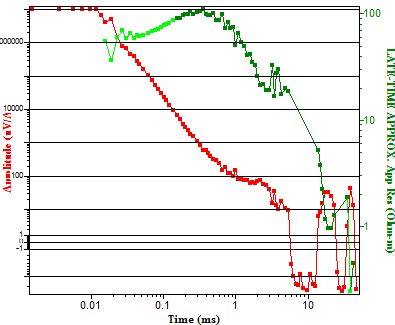 | | | |

| **Station** | **B32** | **Coordinate** |  |
| --- | --- | --- | --- |
|  |  |  |  |
| **Sounding Curve** | | | |
| **Average Decay**  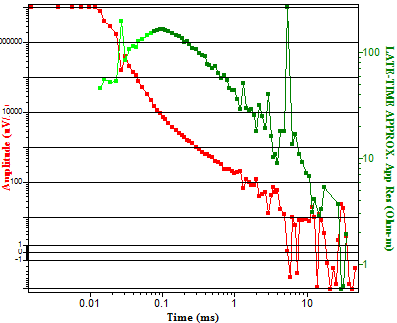 | | | |
| **First Decay**  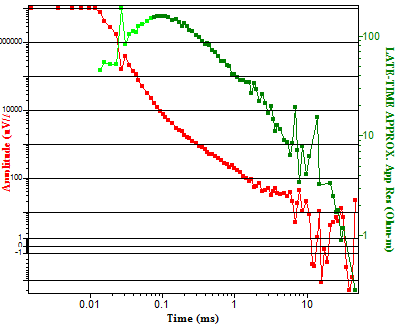 | | | |

| **Station** | **B33** | **Coordinate** |  |
| --- | --- | --- | --- |
|  |  |  |  |
| **Sounding Curve** | | | |
| **Average Decay**  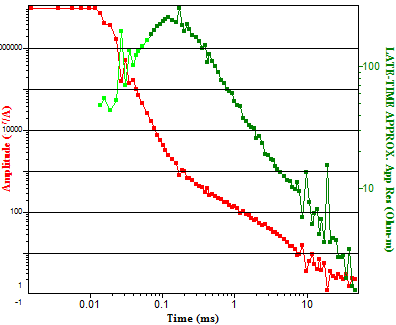 | | | |
| **First Decay**  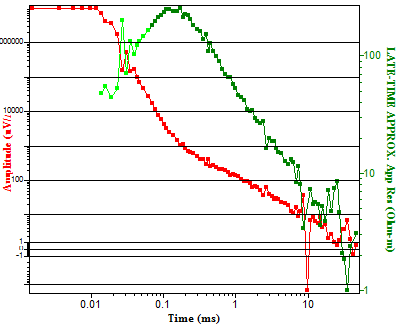 | | | |
