# Supplementary material for: Data for the subsurface characterization of Pahang River Basin with the application of Transient Electromagnetic geophysical surveys
Source: Data Brief. 2020 Apr 23;30:105491. doi: 10.1016/j.dib.2020.105491 (PMC7191212; doi:10.1016/j.dib.2020.105491)
Supplement: Supplementary file 20 [file mmc20.docx]

| **Station** | **C1** | **Coordinate** |  |
| --- | --- | --- | --- |
|  |  |  |  |
| **Sounding Curve** | | | |
| **Average Decay**  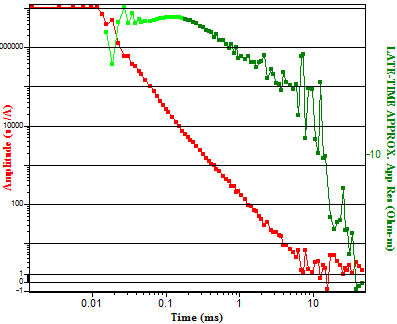 | | | |
| **First Decay**  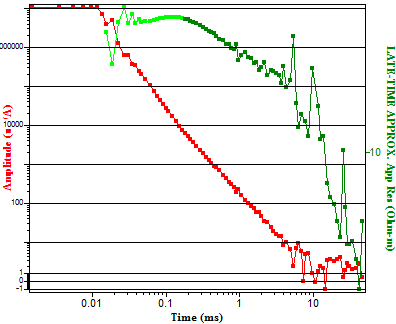 | | | |

| **Station** | **C2** | **Coordinate** |  |
| --- | --- | --- | --- |
|  |  |  |  |
| **Sounding Curve** | | | |
| **Average Decay**  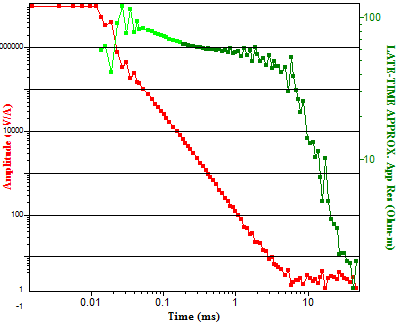 | | | |
| **First Decay**  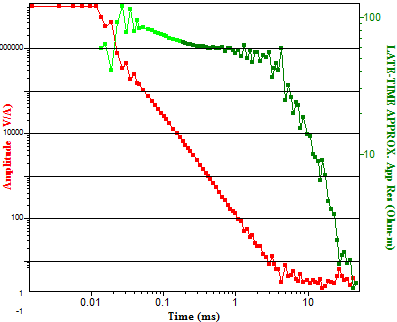 | | | |

| **Station** | **C3** | **Coordinate** |  |
| --- | --- | --- | --- |
|  |  |  |  |
| **Sounding Curve** | | | |
| **Average Decay**  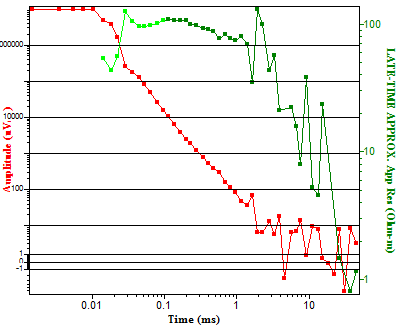 | | | |
| **First Decay**  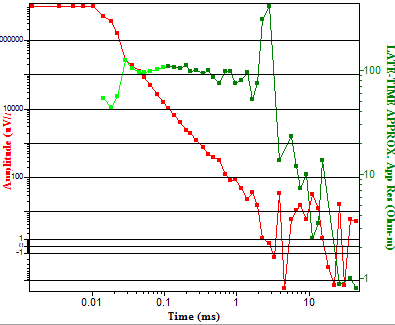 | | | |

| **Station** | **C4** | **Coordinate** |  |
| --- | --- | --- | --- |
|  |  |  |  |
| **Sounding Curve** | | | |
| **Average Decay**  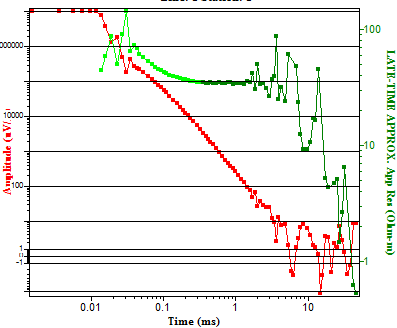 | | | |
| **First Decay**  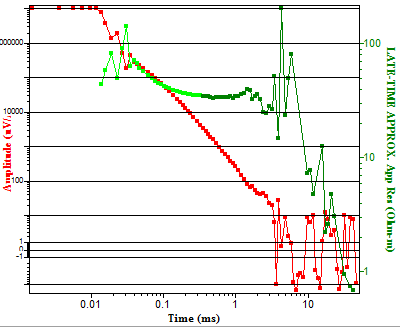 | | | |

| **Station** | **C5** | **Coordinate** |  |
| --- | --- | --- | --- |
|  |  |  |  |
| **Sounding Curve** | | | |
| **Average Decay**  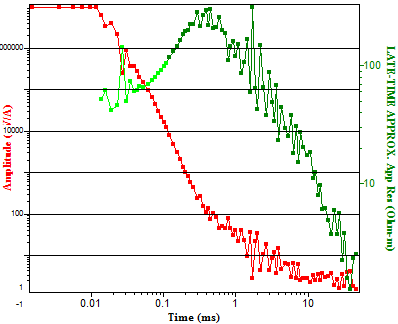 | | | |
| **First Decay**  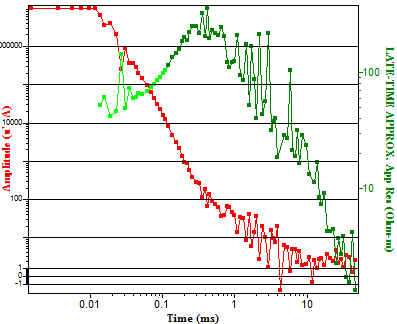 | | | |

| **Station** | **C7** | **Coordinate** |  |
| --- | --- | --- | --- |
|  |  |  |  |
| **Sounding Curve** | | | |
| **Average Decay**  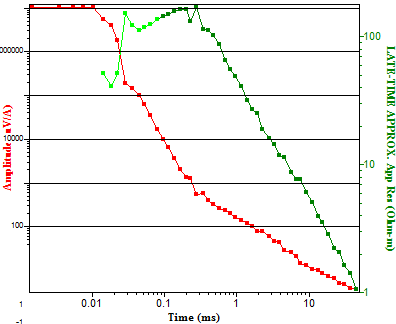 | | | |
| **First Decay**  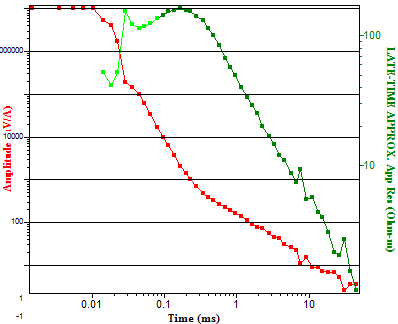 | | | |

| **Station** | **C8** | **Coordinate** |  |
| --- | --- | --- | --- |
|  |  |  |  |
| **Sounding Curve** | | | |
| **Average Decay**  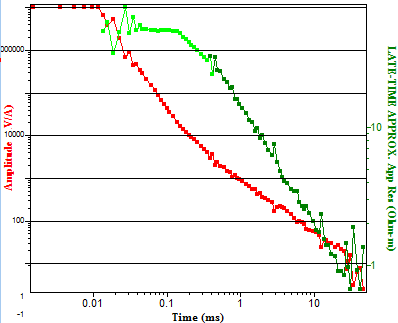 | | | |
| **First Decay**  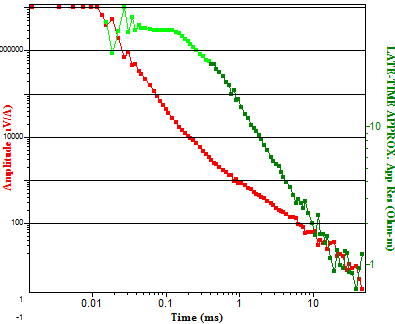 | | | |

| **Station** | **C9** | **Coordinate** |  |
| --- | --- | --- | --- |
|  |  |  |  |
| **Sounding Curve** | | | |
| **Average Decay**  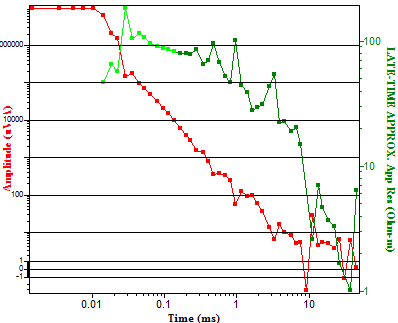 | | | |
| **First Decay**  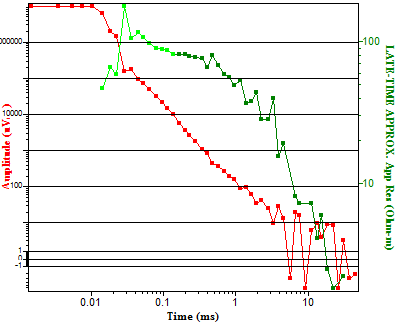 | | | |

| **Station** | **C10** | **Coordinate** |  |
| --- | --- | --- | --- |
|  |  |  |  |
| **Sounding Curve** | | | |
| **Average Decay**  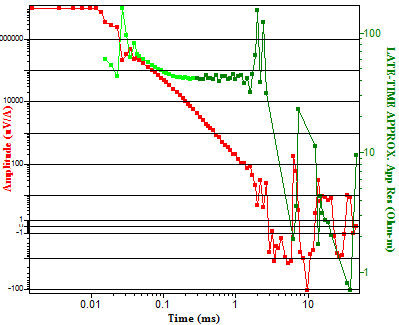 | | | |
| **First Decay**  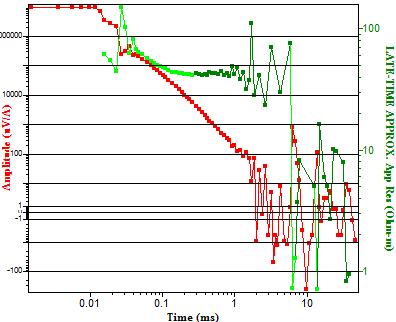 | | | |

| **Station** | **C11** | **Coordinate** |  |
| --- | --- | --- | --- |
|  |  |  |  |
| **Sounding Curve** | | | |
| **Average Decay**  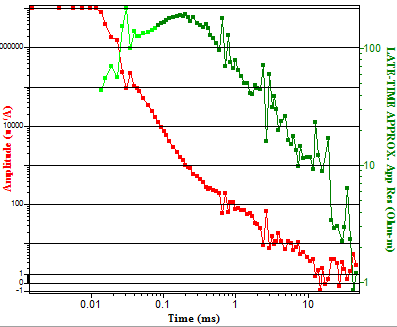 | | | |
| **First Decay**  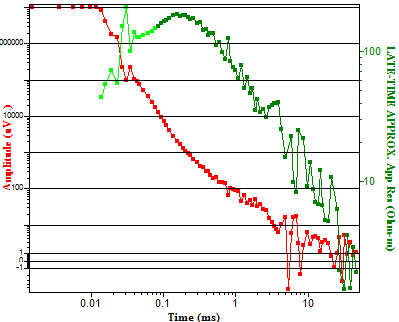 | | | |

| **Station** | **C12** | **Coordinate** |  |
| --- | --- | --- | --- |
|  |  |  |  |
| **Sounding Curve** | | | |
| **Average Decay**  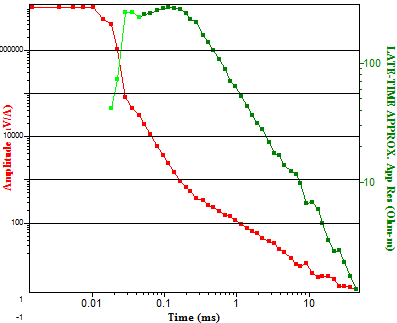 | | | |
| **First Decay**  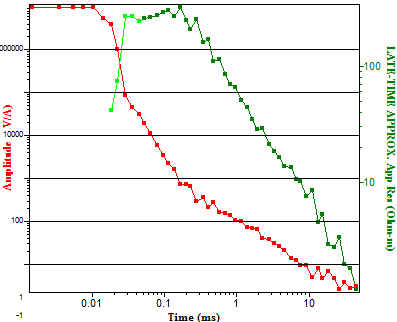 | | | |

| **Station** | **C13** | **Coordinate** |  |
| --- | --- | --- | --- |
|  |  |  |  |
| **Sounding Curve** | | | |
| **Average Decay**  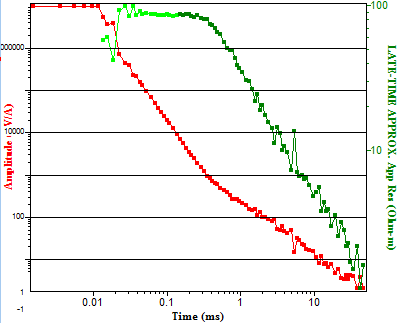 | | | |
| **First Decay**  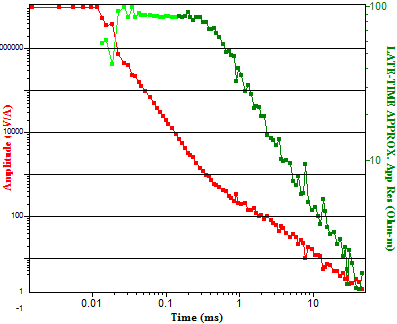 | | | |

| **Station** | **C14** | **Coordinate** |  |
| --- | --- | --- | --- |
|  |  |  |  |
| **Sounding Curve** | | | |
| **Average Decay**  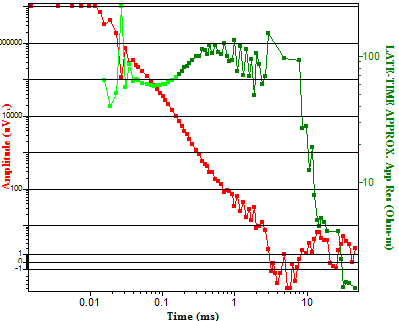 | | | |
| **First Decay**  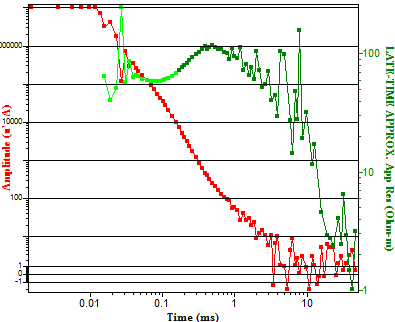 | | | |

| **Station** | **C15** | **Coordinate** |  |
| --- | --- | --- | --- |
|  |  |  |  |
| **Sounding Curve** | | | |
| **Average Decay**  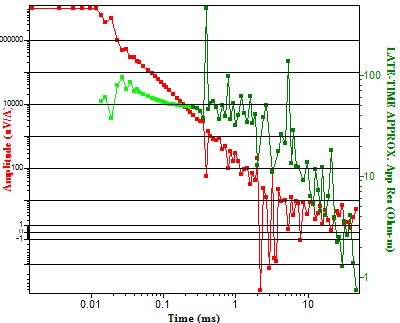 | | | |
| **First Decay**  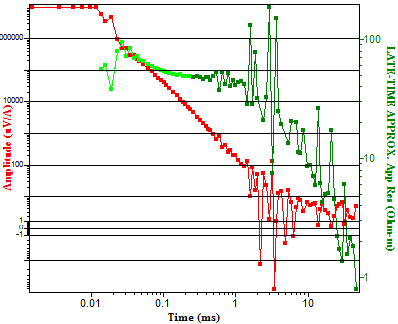 | | | |

| **Station** | **C16** | **Coordinate** |  |
| --- | --- | --- | --- |
|  |  |  |  |
| **Sounding Curve** | | | |
| **Average Decay**  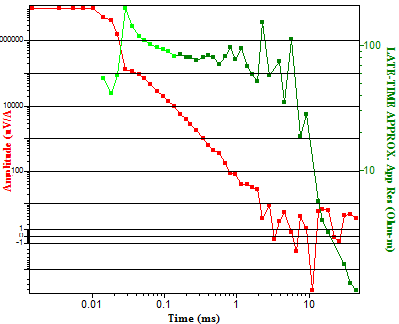 | | | |
| **First Decay**  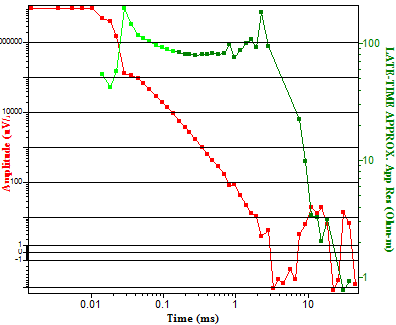 | | | |

| **Station** | **C17** | **Coordinate** |  |
| --- | --- | --- | --- |
|  |  |  |  |
| **Sounding Curve** | | | |
| **Average Decay**  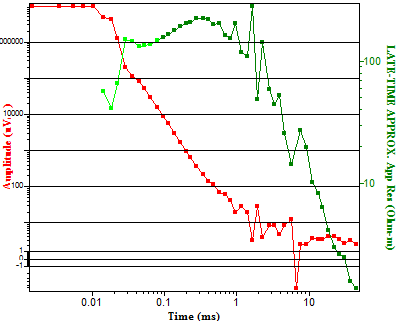 | | | |
| **First Decay**  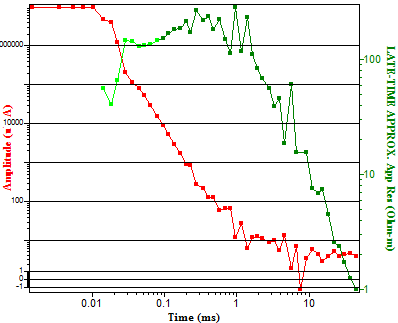 | | | |

| **Station** | **C18** | **Coordinate** |  |
| --- | --- | --- | --- |
|  |  |  |  |
| **Sounding Curve** | | | |
| **Average Decay**  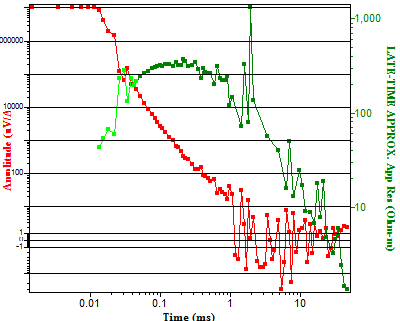 | | | |
| **First Decay**  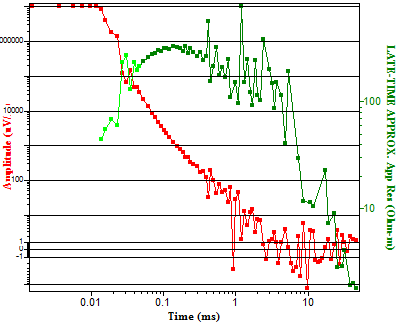 | | | |

| **Station** | **C19** | **Coordinate** |  |
| --- | --- | --- | --- |
|  |  |  |  |
| **Sounding Curve** | | | |
| **Average Decay**  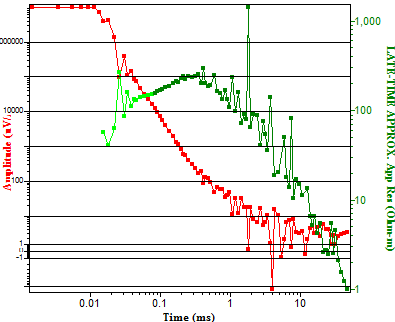 | | | |
| **First Decay**  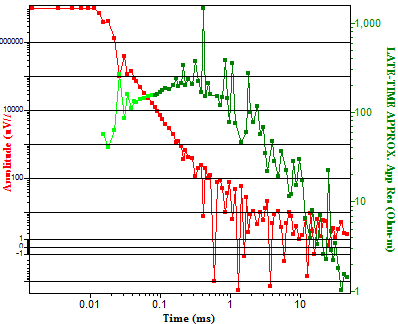 | | | |

| **Station** | **C20** | **Coordinate** |  |
| --- | --- | --- | --- |
|  |  |  |  |
| **Sounding Curve** | | | |
| **Average Decay**  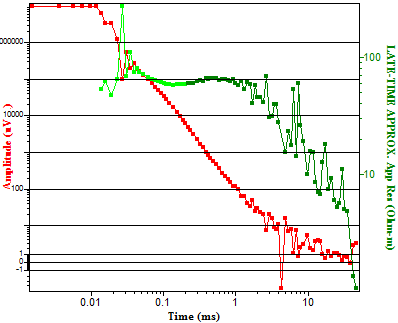 | | | |
| **First Decay**  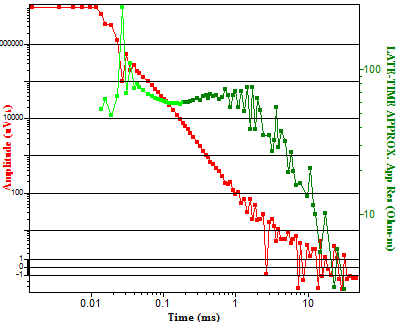 | | | |

| **Station** | **C21** | **Coordinate** |  |
| --- | --- | --- | --- |
|  |  |  |  |
| **Sounding Curve** | | | |
| **Average Decay**  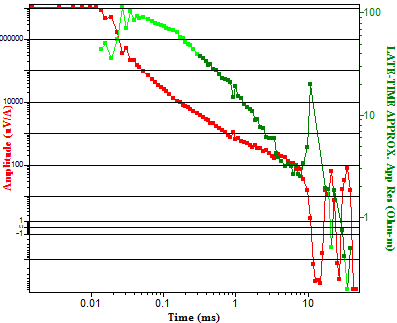 | | | |
| **First Decay**  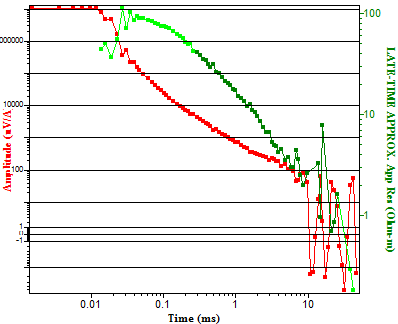 | | | |

| **Station** | **C22** | **Coordinate** |  |
| --- | --- | --- | --- |
|  |  |  |  |
| **Sounding Curve** | | | |
| **Average Decay**  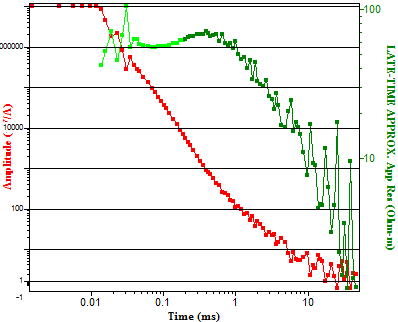 | | | |
| **First Decay**  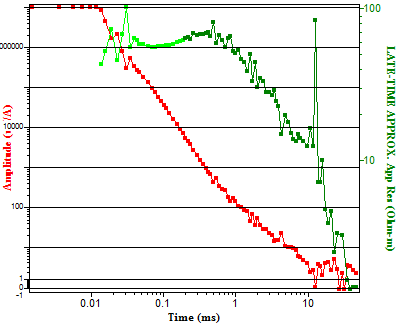 | | | |

| **Station** | **C23** | **Coordinate** |  |
| --- | --- | --- | --- |
|  |  |  |  |
| **Sounding Curve** | | | |
| **Average Decay**  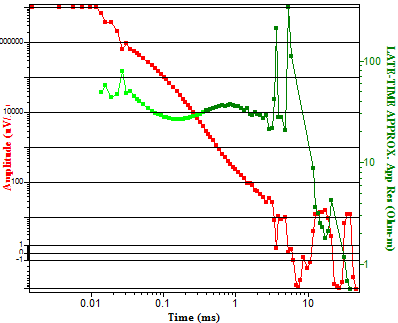 | | | |
| **First Decay**  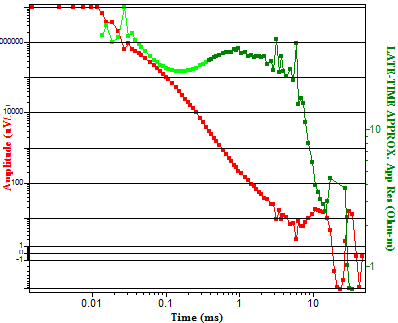 | | | |

| **Station** | **C24** | **Coordinate** |  |
| --- | --- | --- | --- |
|  |  |  |  |
| **Sounding Curve** | | | |
| **Average Decay**  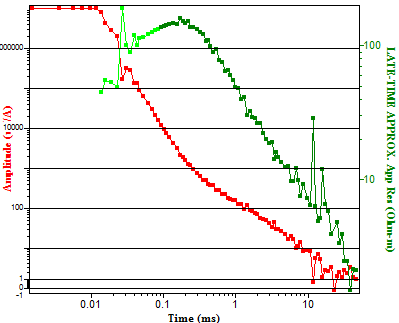 | | | |
| **First Decay**  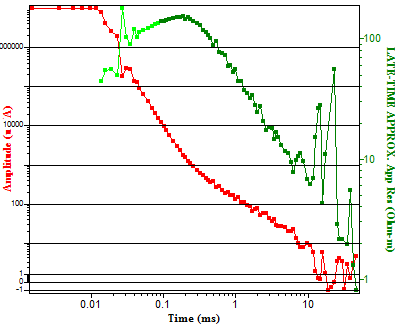 | | | |

| **Station** | **C25** | **Coordinate** |  |
| --- | --- | --- | --- |
|  |  |  |  |
| **Sounding Curve** | | | |
| **Average Decay**  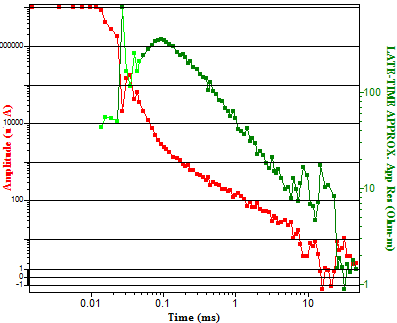 | | | |
| **First Decay**  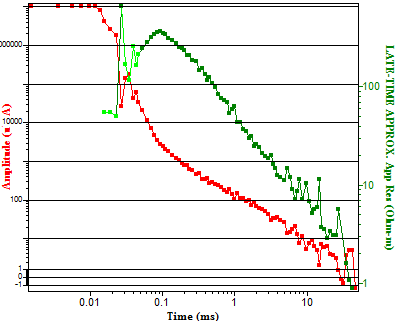 | | | |

| **Station** | **C26** | **Coordinate** |  |
| --- | --- | --- | --- |
|  |  |  |  |
| **Sounding Curve** | | | |
| **Average Decay**  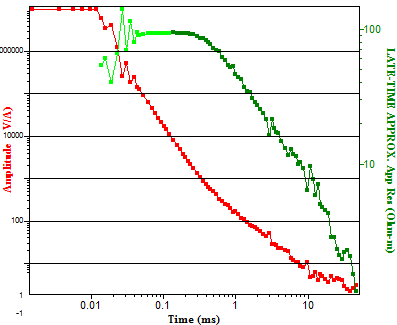 | | | |
| **First Decay**  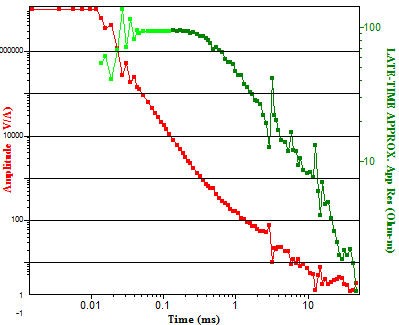 | | | |

| **Station** | **C27** | **Coordinate** |  |
| --- | --- | --- | --- |
|  |  |  |  |
| **Sounding Curve** | | | |
| **Average Decay**  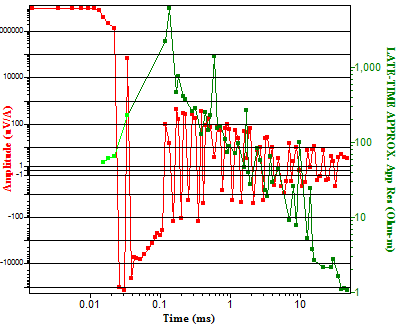 | | | |
| **First Decay**  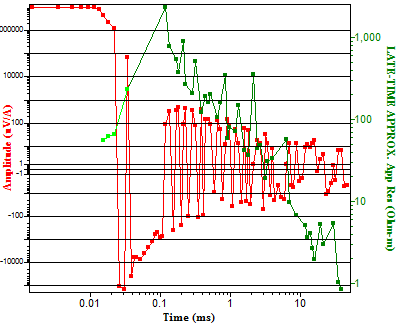 | | | |

| **Station** | **C28** | **Coordinate** |  |
| --- | --- | --- | --- |
|  |  |  |  |
| **Sounding Curve** | | | |
| **Average Decay**  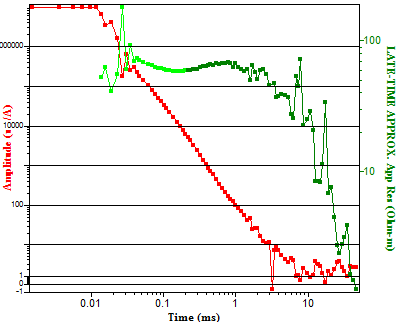 | | | |
| **First Decay**  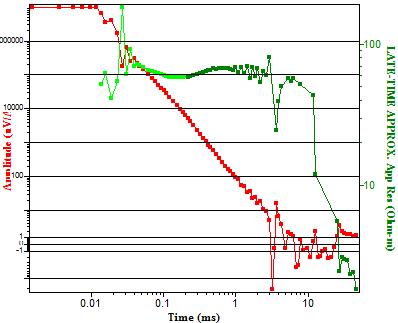 | | | |

| **Station** | **C29** | **Coordinate** |  |
| --- | --- | --- | --- |
|  |  |  |  |
| **Sounding Curve** | | | |
| **Average Decay**  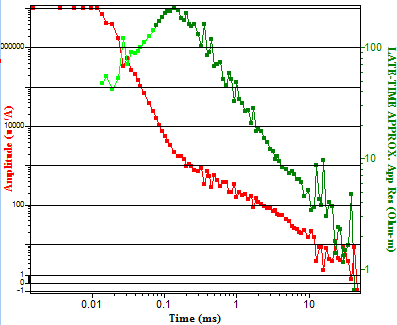 | | | |
| **First Decay**  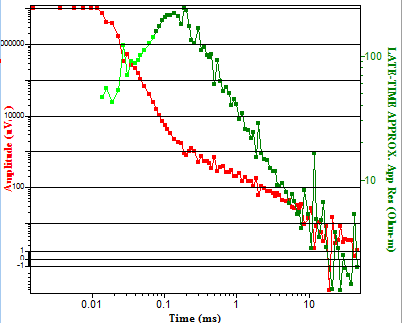 | | | |

| **Station** | **C30** | **Coordinate** |  |
| --- | --- | --- | --- |
|  |  |  |  |
| **Sounding Curve** | | | |
| **Average Decay**  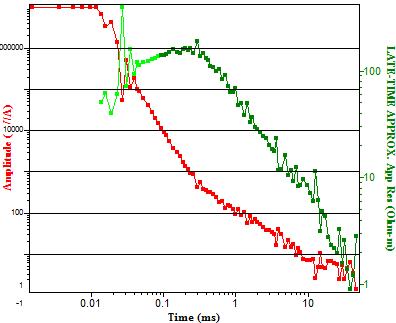 | | | |
| **First Decay**  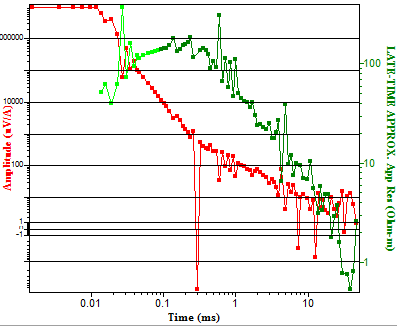 | | | |
