# Supplementary material for: Data for the subsurface characterization of Pahang River Basin with the application of Transient Electromagnetic geophysical surveys
Source: Data Brief. 2020 Apr 23;30:105491. doi: 10.1016/j.dib.2020.105491 (PMC7191212; doi:10.1016/j.dib.2020.105491)
Supplement: Supplementary file 22 [file mmc22.docx]

| **Station** | **E1** | **Coordinate** |  |
| --- | --- | --- | --- |
|  |  |  |  |
| **Sounding Curve** | | | |
| **Average Decay**  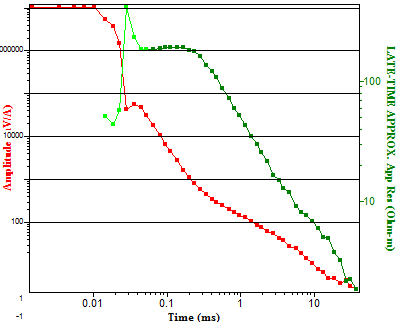 | | | |
| **First Decay**  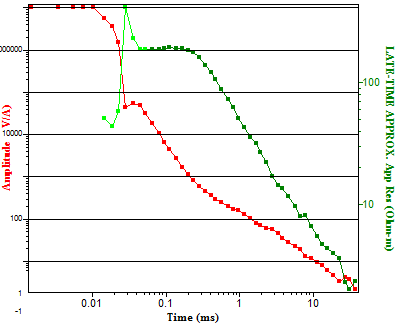 | | | |

| **Station** | **E2** | **Coordinate** |  |
| --- | --- | --- | --- |
|  |  |  |  |
| **Sounding Curve** | | | |
| **Average Decay**  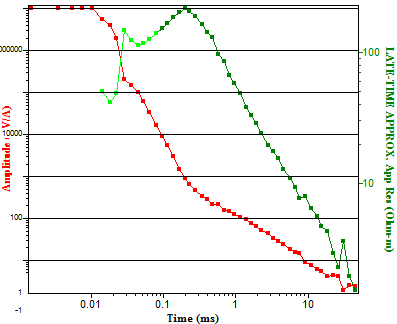 | | | |
| **First Decay**  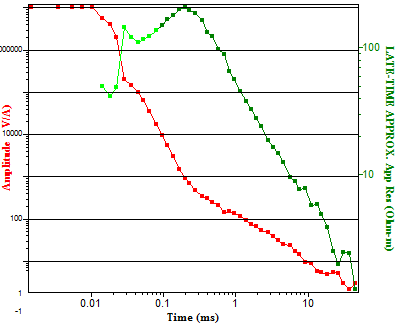 | | | |

| **Station** | **E3** | **Coordinate** |  |
| --- | --- | --- | --- |
|  |  |  |  |
| **Sounding Curve** | | | |
| **Average Decay**  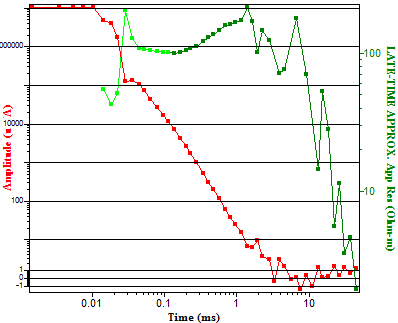 | | | |
| **First Decay**  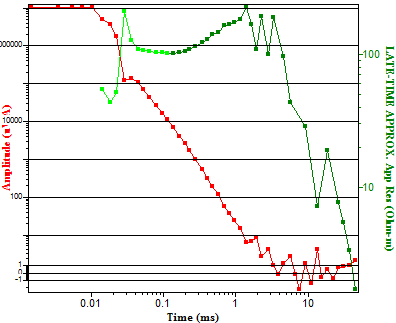 | | | |

| **Station** | **E4** | **Coordinate** |  |
| --- | --- | --- | --- |
|  |  |  |  |
| **Sounding Curve** | | | |
| **Average Decay**  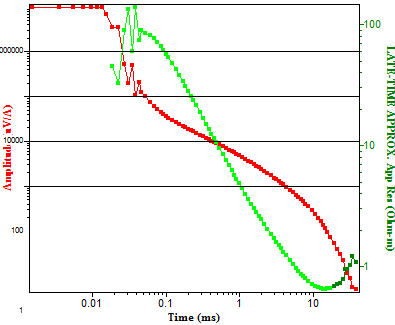 | | | |
| **First Decay**  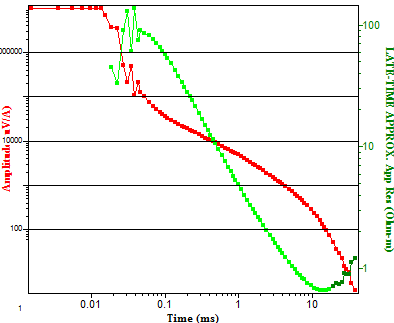 | | | |

| **Station** | **E5** | **Coordinate** |  |
| --- | --- | --- | --- |
|  |  |  |  |
| **Sounding Curve** | | | |
| **Average Decay**  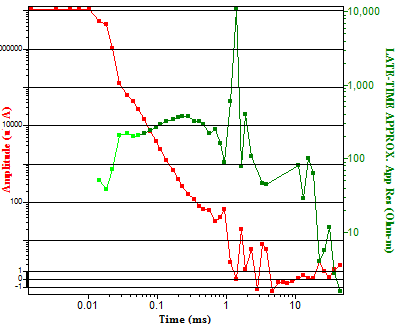 | | | |
| **First Decay**  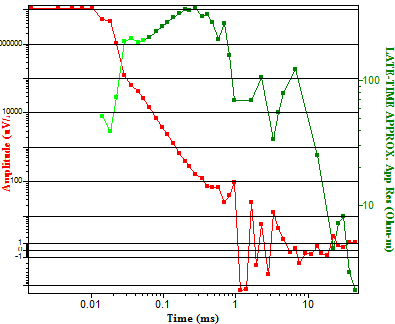 | | | |

| **Station** | **E6** | **Coordinate** |  |
| --- | --- | --- | --- |
|  |  |  |  |
| **Sounding Curve** | | | |
| **Average Decay**  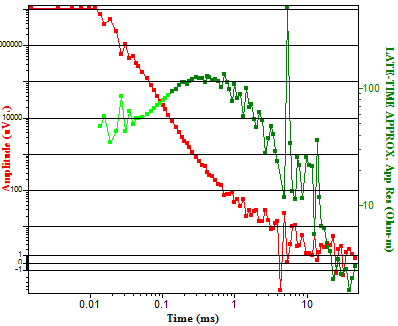 | | | |
| **First Decay**  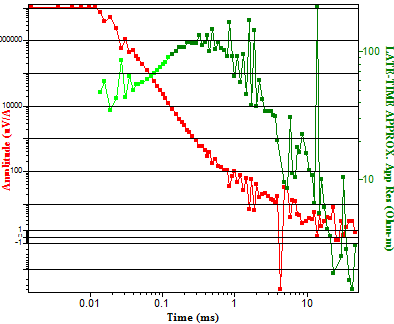 | | | |

| **Station** | **E7** | **Coordinate** |  |
| --- | --- | --- | --- |
|  |  |  |  |
| **Sounding Curve** | | | |
| **Average Decay**  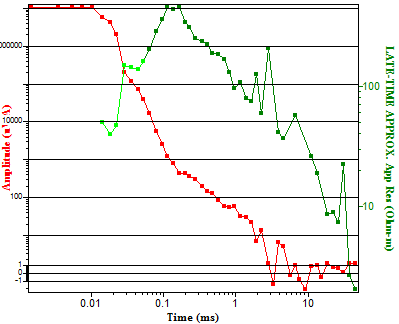 | | | |
| **First Decay**  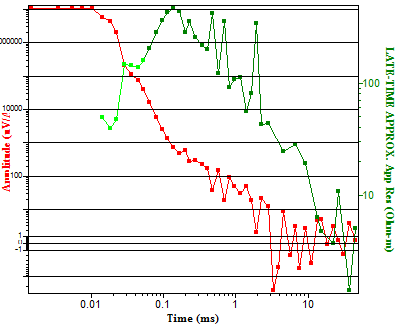 | | | |

| **Station** | **E8** | **Coordinate** |  |
| --- | --- | --- | --- |
|  |  |  |  |
| **Sounding Curve** | | | |
| **Average Decay**  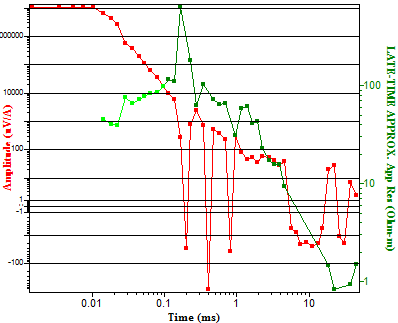 | | | |
| **First Decay**  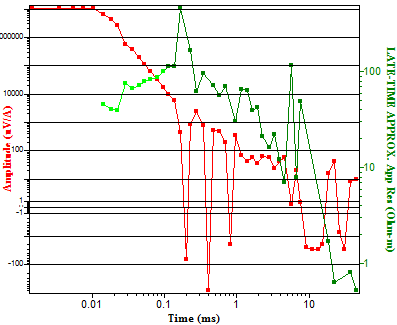 | | | |

| **Station** | **E9** | **Coordinate** |  |
| --- | --- | --- | --- |
|  |  |  |  |
| **Sounding Curve** | | | |
| **Average Decay**  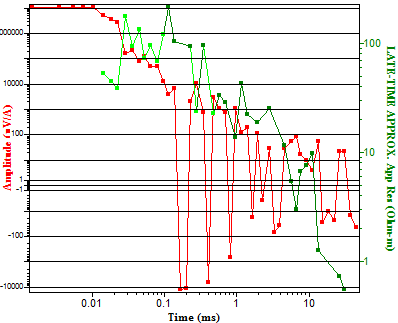 | | | |
| **First Decay**  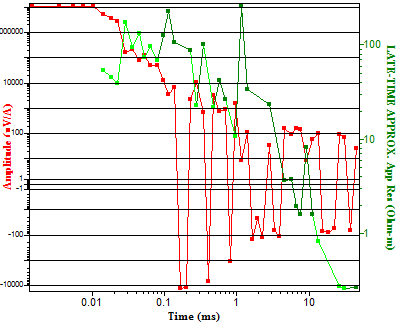 | | | |

| **Station** | **E10** | **Coordinate** |  |
| --- | --- | --- | --- |
|  |  |  |  |
| **Sounding Curve** | | | |
| **Average Decay**  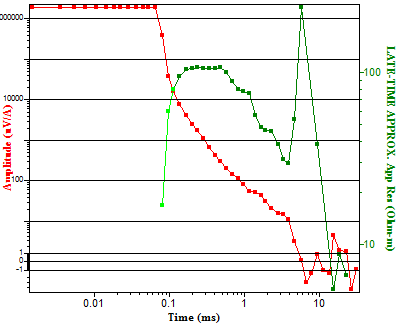 | | | |
| **First Decay**  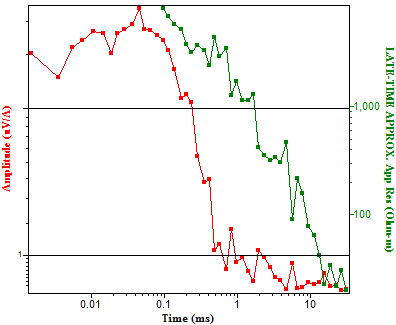 | | | |

| **Station** | **E11** | **Coordinate** |  |
| --- | --- | --- | --- |
|  |  |  |  |
| **Sounding Curve** | | | |
| **Average Decay**  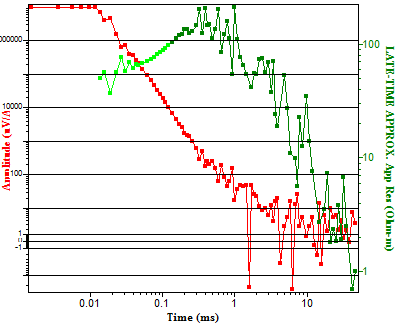 | | | |
| **First Decay**  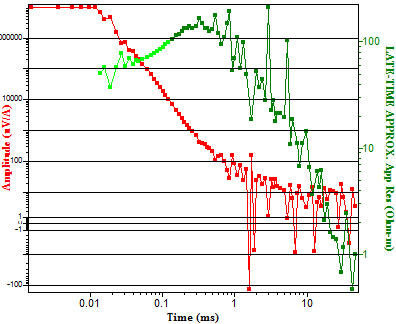 | | | |

| **Station** | **E12** | **Coordinate** |  |
| --- | --- | --- | --- |
|  |  |  |  |
| **Sounding Curve** | | | |
| **First Decay**  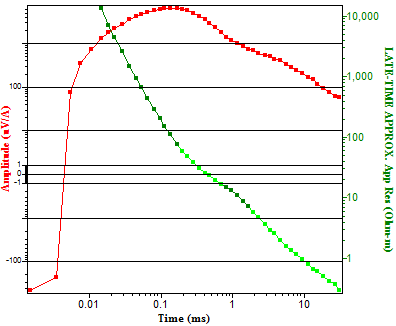 | | | |
|  | | | |

| **Station** | **E14** | **Coordinate** |  |
| --- | --- | --- | --- |
|  |  |  |  |
| **Sounding Curve** | | | |
| **First Decay**  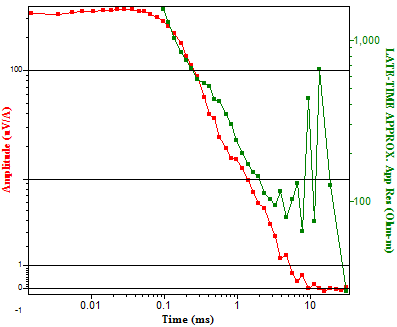 | | | |
|  | | | |

| **Station** | **E15** | **Coordinate** |  |
| --- | --- | --- | --- |
|  |  |  |  |
| **Sounding Curve** | | | |
| **First Decay**  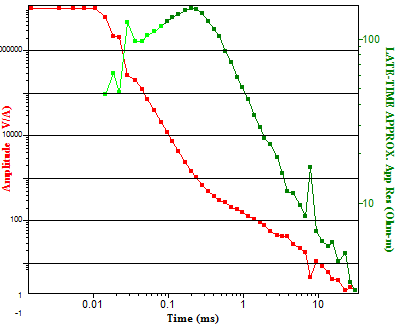 | | | |
|  | | | |

| **Station** | **E16** | **Coordinate** |  |
| --- | --- | --- | --- |
|  |  |  |  |
| **Sounding Curve** | | | |
| **Average Decay**  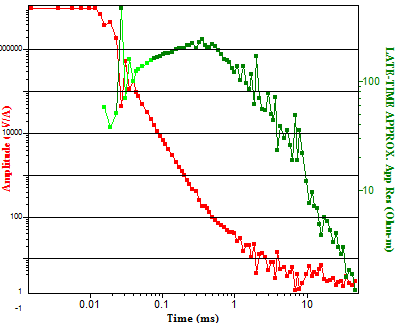 | | | |
| **First Decay**  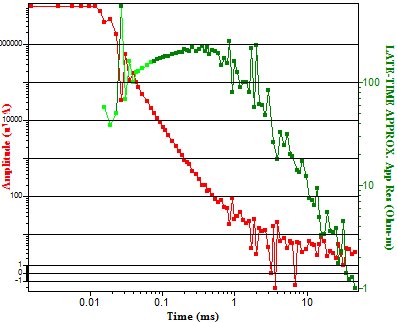 | | | |

| **Station** | **E17** | **Coordinate** |  |
| --- | --- | --- | --- |
|  |  |  |  |
| **Sounding Curve** | | | |
| **First Decay**  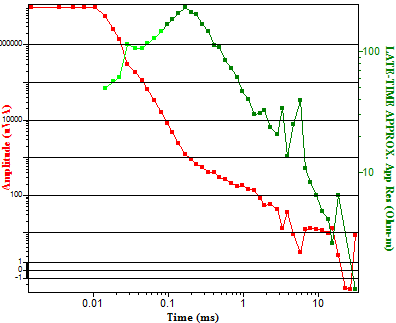 | | | |
|  | | | |

| **Station** | **E18** | **Coordinate** |  |
| --- | --- | --- | --- |
|  |  |  |  |
| **Sounding Curve** | | | |
| **Average Decay**  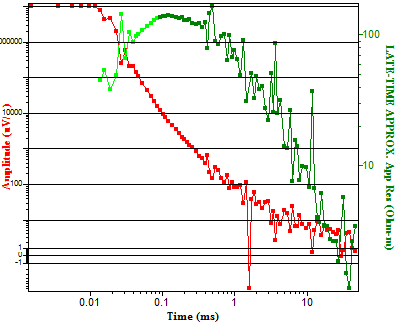 | | | |
| **First Decay**  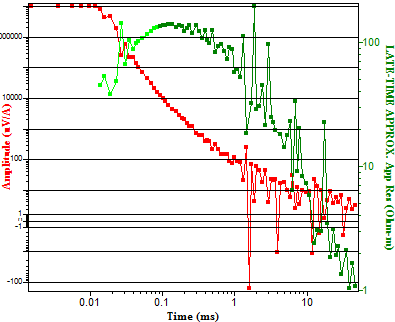 | | | |

| **Station** | **E19** | **Coordinate** |  |
| --- | --- | --- | --- |
|  |  |  |  |
| **Sounding Curve** | | | |
| **Average Decay**  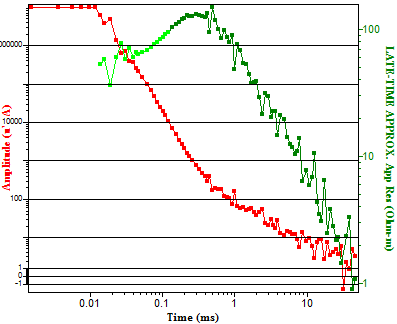 | | | |
| **First Decay**  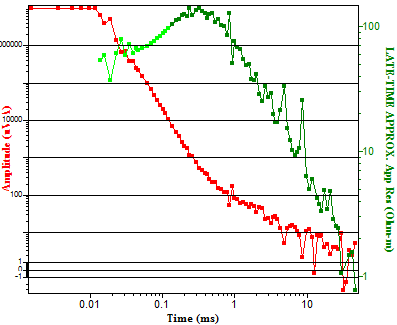 | | | |

| **Station** | **E20** | **Coordinate** |  |
| --- | --- | --- | --- |
|  |  |  |  |
| **Sounding Curve** | | | |
| **Average Decay**  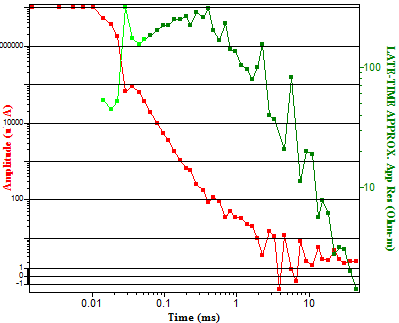 | | | |
| **First Decay**  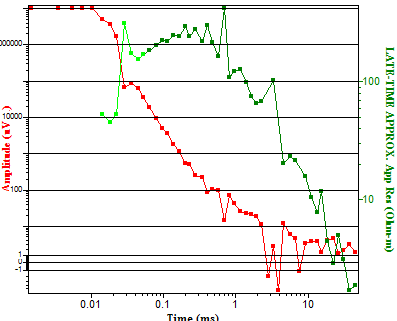 | | | |

| **Station** | **E21** | **Coordinate** |  |
| --- | --- | --- | --- |
|  |  |  |  |
| **Sounding Curve** | | | |
| **First Decay**  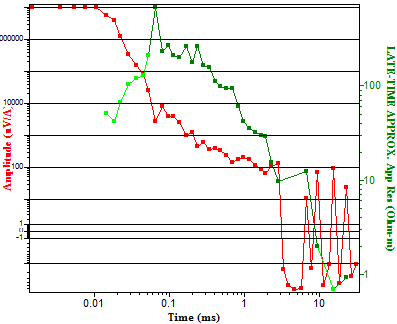 | | | |
|  | | | |
