# Supplementary material for: Data for the subsurface characterization of Pahang River Basin with the application of Transient Electromagnetic geophysical surveys
Source: Data Brief. 2020 Apr 23;30:105491. doi: 10.1016/j.dib.2020.105491 (PMC7191212; doi:10.1016/j.dib.2020.105491)
Supplement: Supplementary file 24 [file mmc24.docx]

| **Station** | **G1** | **Coordinate** |  |
| --- | --- | --- | --- |
|  |  |  |  |
| **Sounding Curve** | | | |
| **Average Decay**  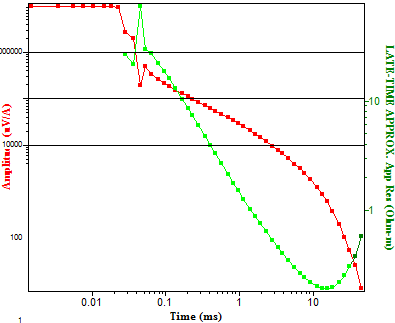 | | | |
| **First Decay**  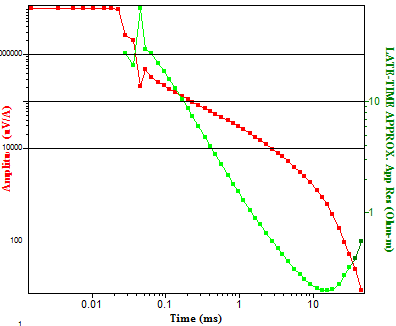 | | | |

| **Station** | **G2** | **Coordinate** |  |
| --- | --- | --- | --- |
|  |  |  |  |
| **Sounding Curve** | | | |
| **Average Decay**  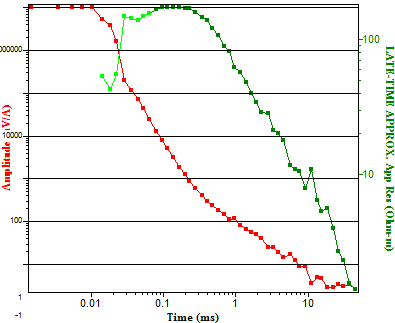 | | | |
| **First Decay**  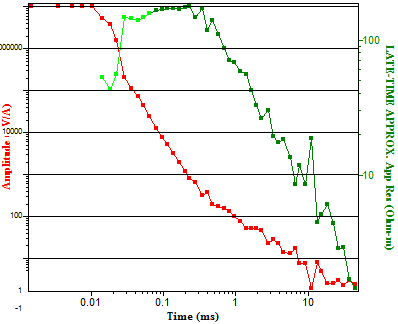 | | | |

| **Station** | **G3** | **Coordinate** |  |
| --- | --- | --- | --- |
|  |  |  |  |
| **Sounding Curve** | | | |
| **Average Decay**  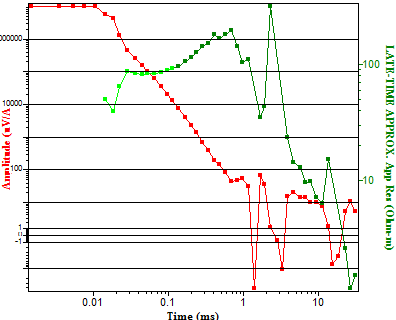 | | | |
| **First Decay**  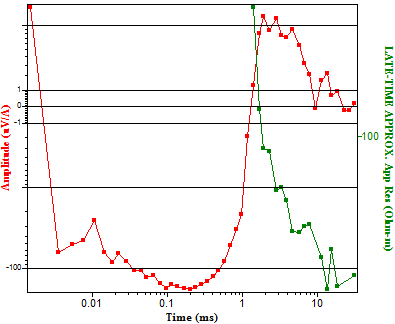 | | | |

| **Station** | **G4** | **Coordinate** |  |
| --- | --- | --- | --- |
|  |  |  |  |
| **Sounding Curve** | | | |
| **First Decay**  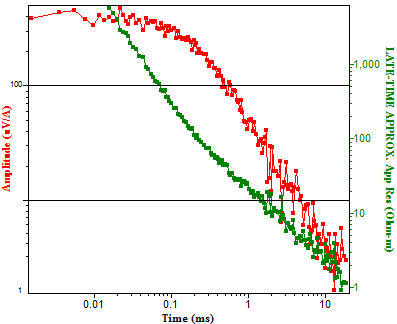 | | | |
|  | | | |

| **Station** | **G5** | **Coordinate** |  |
| --- | --- | --- | --- |
|  |  |  |  |
| **Sounding Curve** | | | |
| **Average Decay**  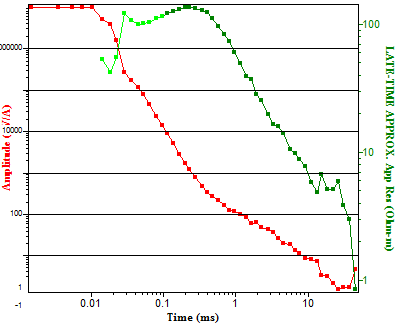 | | | |
| **First Decay**  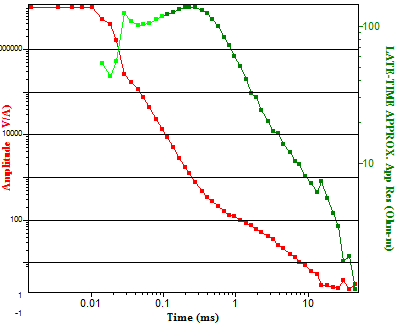 | | | |

| **Station** | **G6** | **Coordinate** |  |
| --- | --- | --- | --- |
|  |  |  |  |
| **Sounding Curve** | | | |
| **Average Decay**  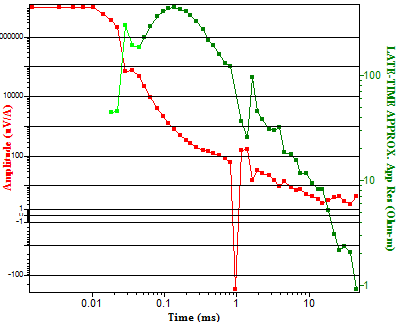 | | | |
| **First Decay**  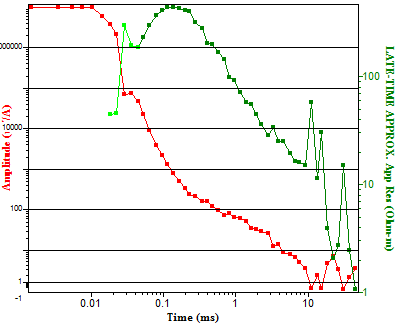 | | | |

| **Station** | **G7** | **Coordinate** |  |
| --- | --- | --- | --- |
|  |  |  |  |
| **Sounding Curve** | | | |
| **Average Decay**  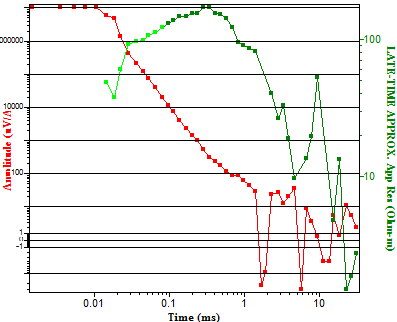 | | | |
| **First Decay**  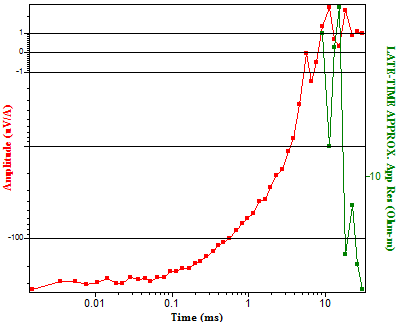 | | | |

| **Station** | **G8** | **Coordinate** |  |
| --- | --- | --- | --- |
|  |  |  |  |
| **Sounding Curve** | | | |
| **First Decay**  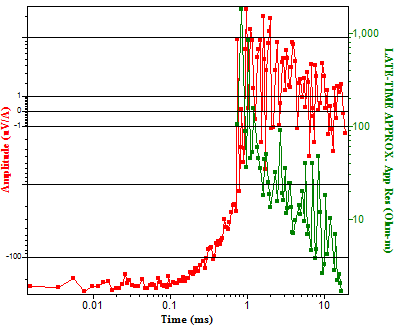 | | | |
|  | | | |

| **Station** | **G9** | **Coordinate** |  |
| --- | --- | --- | --- |
|  |  |  |  |
| **Sounding Curve** | | | |
| **Average Decay**  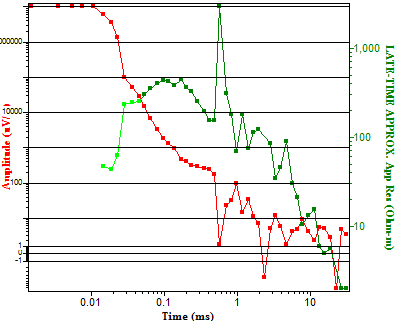 | | | |
| **First Decay**  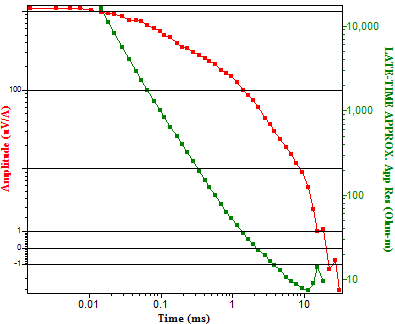 | | | |

| **Station** | **G10** | **Coordinate** |  |
| --- | --- | --- | --- |
|  |  |  |  |
| **Sounding Curve** | | | |
| **First Decay**  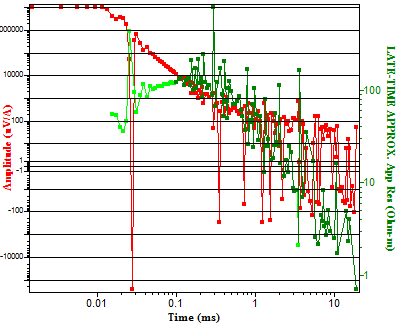 | | | |
|  | | | |

| **Station** | **G12** | **Coordinate** |  |
| --- | --- | --- | --- |
|  |  |  |  |
| **Sounding Curve** | | | |
| **First Decay**  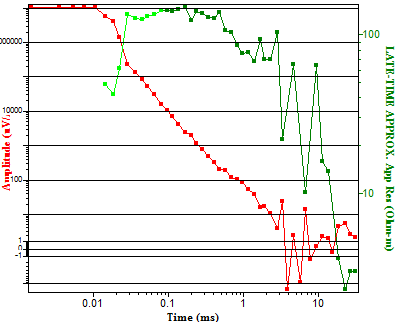 | | | |
|  | | | |

| **Station** | **G13** | **Coordinate** |  |
| --- | --- | --- | --- |
|  |  |  |  |
| **Sounding Curve** | | | |
| **Average Decay**  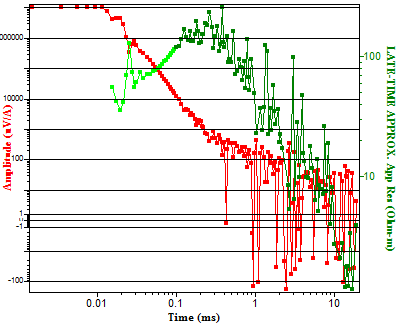 | | | |
| **First Decay**  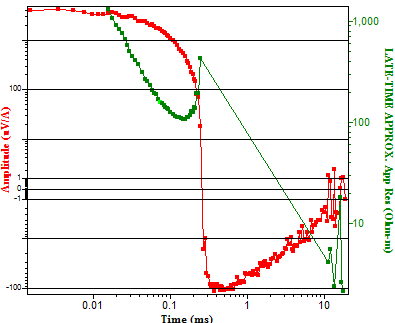 | | | |

| **Station** | **G14** | **Coordinate** |  |
| --- | --- | --- | --- |
|  |  |  |  |
| **Sounding Curve** | | | |
| **Average Decay**  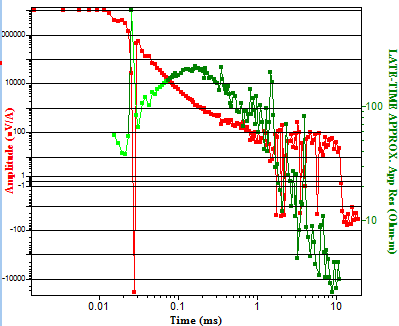 | | | |
| **First Decay**  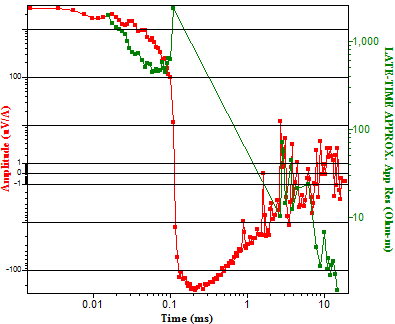 | | | |

| **Station** | **G15** | **Coordinate** |  |
| --- | --- | --- | --- |
|  |  |  |  |
| **Sounding Curve** | | | |
| **First Decay**  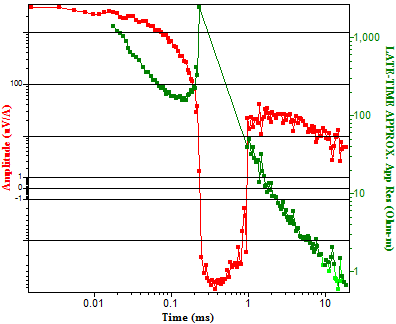 | | | |
|  | | | |

| **Station** | **G16** | **Coordinate** |  |
| --- | --- | --- | --- |
|  |  |  |  |
| **Sounding Curve** | | | |
| **Average Decay**  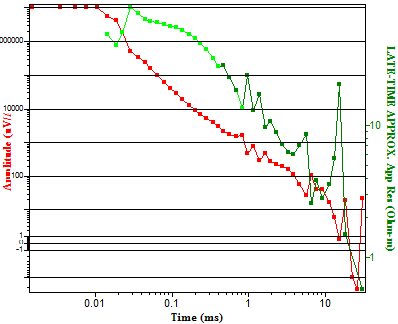 | | | |
| **First Decay**  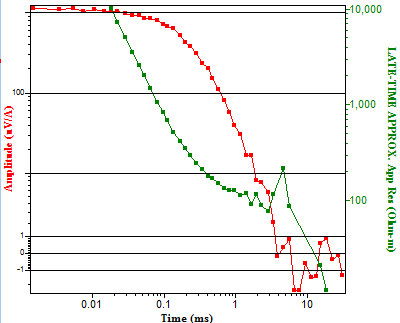 | | | |

| **Station** | **G17** | **Coordinate** |  |
| --- | --- | --- | --- |
|  |  |  |  |
| **Sounding Curve** | | | |
| **Average Decay**  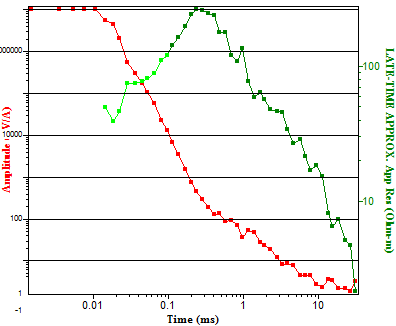 | | | |
| **First Decay**  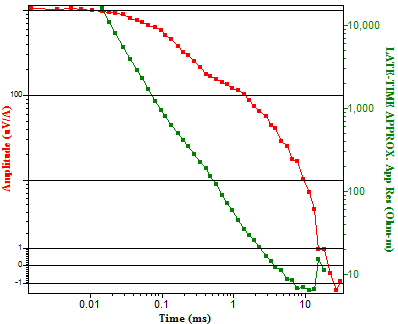 | | | |

| **Station** | **G18** | **Coordinate** |  |
| --- | --- | --- | --- |
|  |  |  |  |
| **Sounding Curve** | | | |
| **Average Decay**  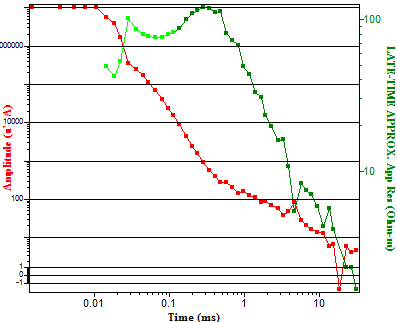 | | | |
| **First Decay**  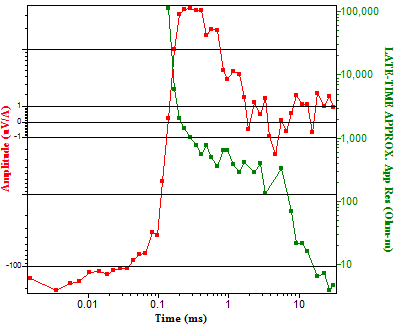 | | | |

| **Station** | **G21** | **Coordinate** |  |
| --- | --- | --- | --- |
|  |  |  |  |
| **Sounding Curve** | | | |
| **Average Decay**  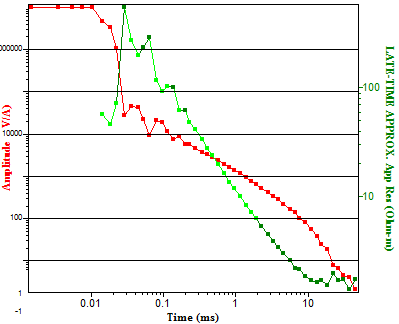 | | | |
| **First Decay**  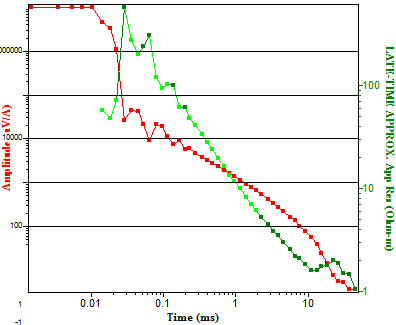 | | | |

| **Station** | **G22** | **Coordinate** |  |
| --- | --- | --- | --- |
|  |  |  |  |
| **Sounding Curve** | | | |
| **Average Decay**  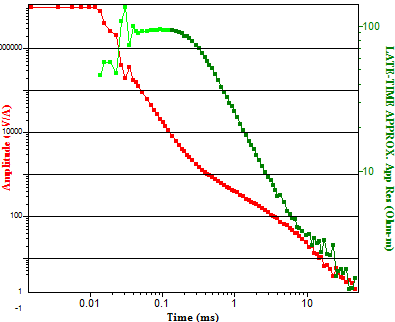 | | | |
| **First Decay**  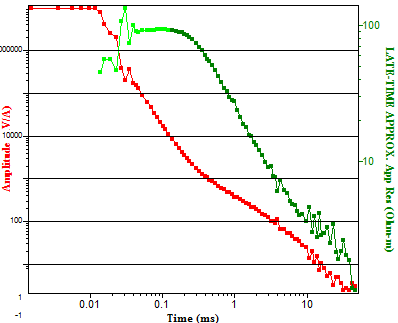 | | | |

| **Station** | **G23** | **Coordinate** |  |
| --- | --- | --- | --- |
|  |  |  |  |
| **Sounding Curve** | | | |
| **Average Decay**  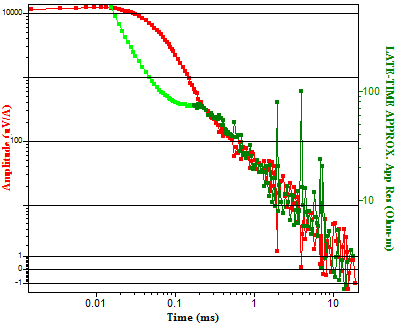 | | | |
| **First Decay** | | | |

| **Station** | **G24** | **Coordinate** |  |
| --- | --- | --- | --- |
|  |  |  |  |
| **Sounding Curve** | | | |
| **Average Decay**  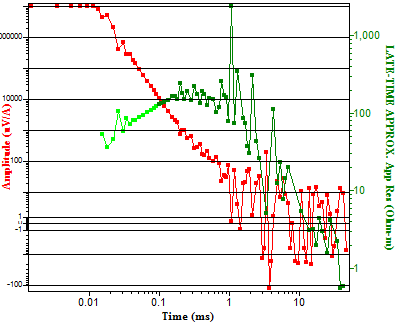 | | | |
| **First Decay**  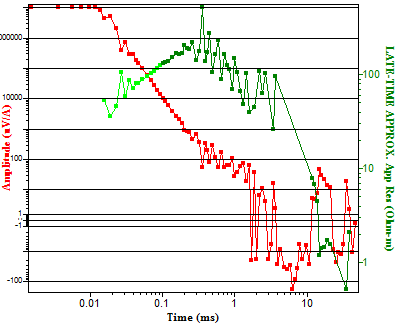 | | | |
