# Supplementary figures and images for: Data for the subsurface characterization of Pahang River Basin with the application of Transient Electromagnetic geophysical surveys
Source: Data Brief. 2020 Apr 23;30:105491. doi: 10.1016/j.dib.2020.105491 (PMC7191212; doi:10.1016/j.dib.2020.105491)

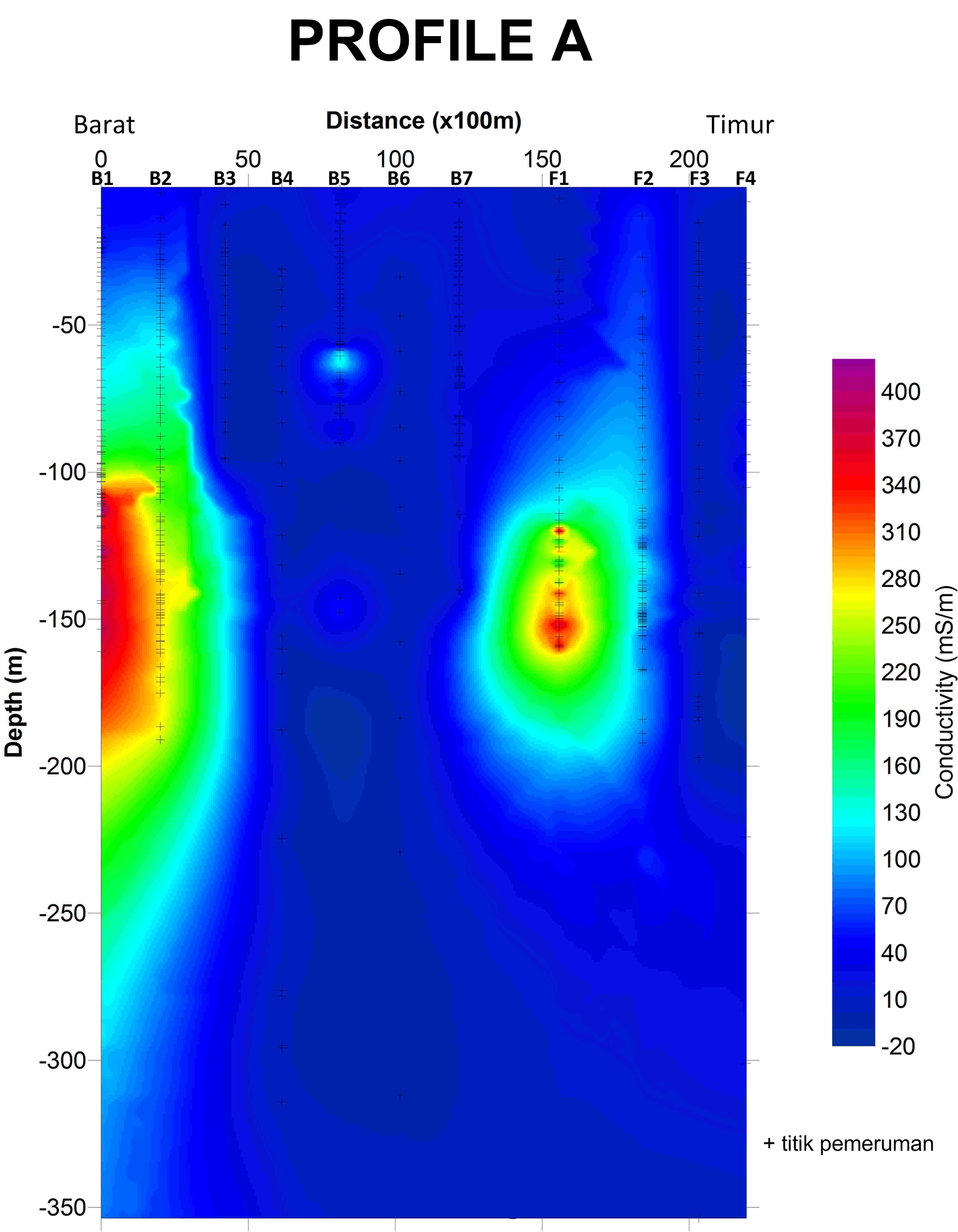

Supplement: Supplementary file 42 [file mmc42.jpg]

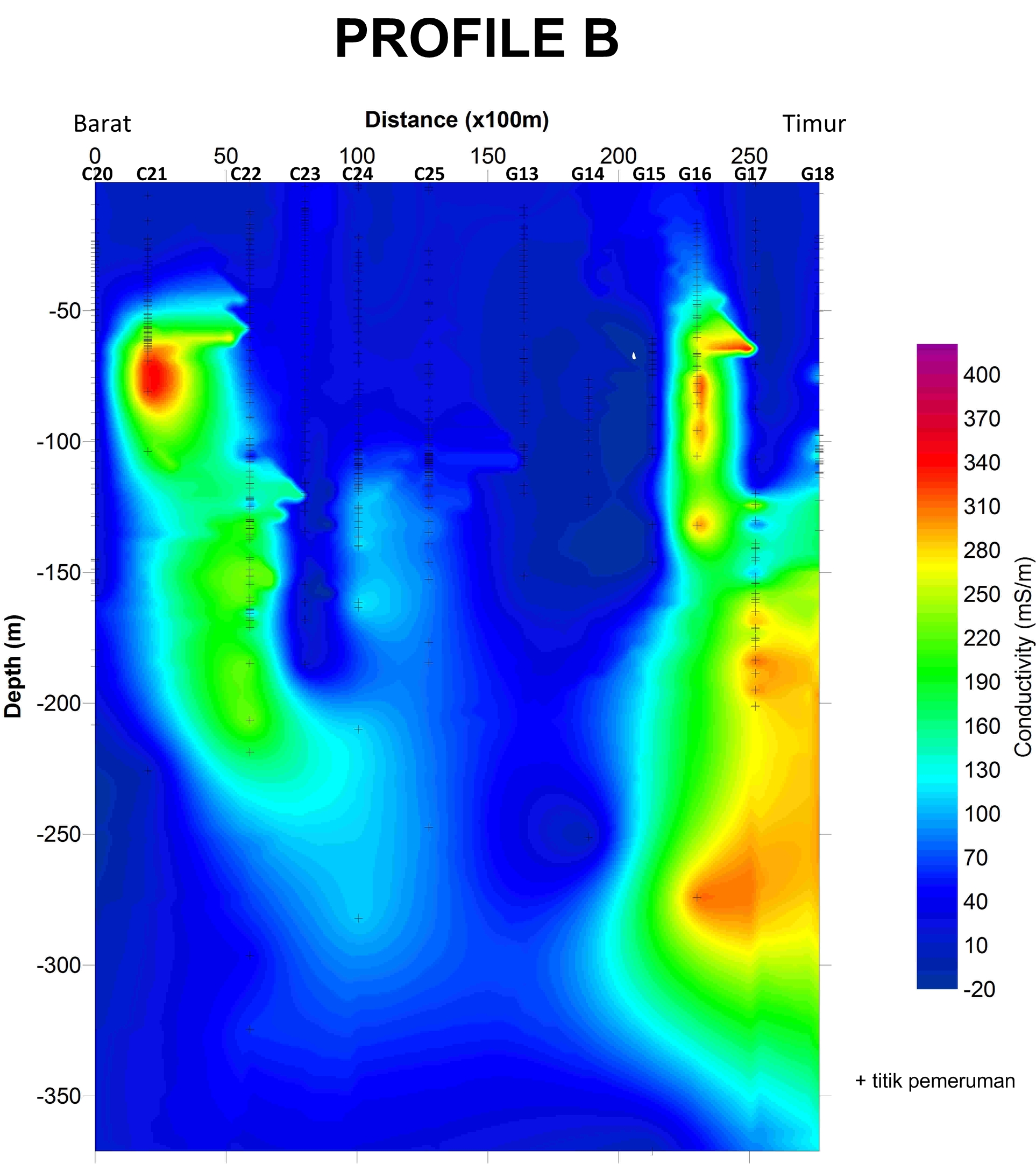

Supplement: Supplementary file 43 [file mmc43.jpg]

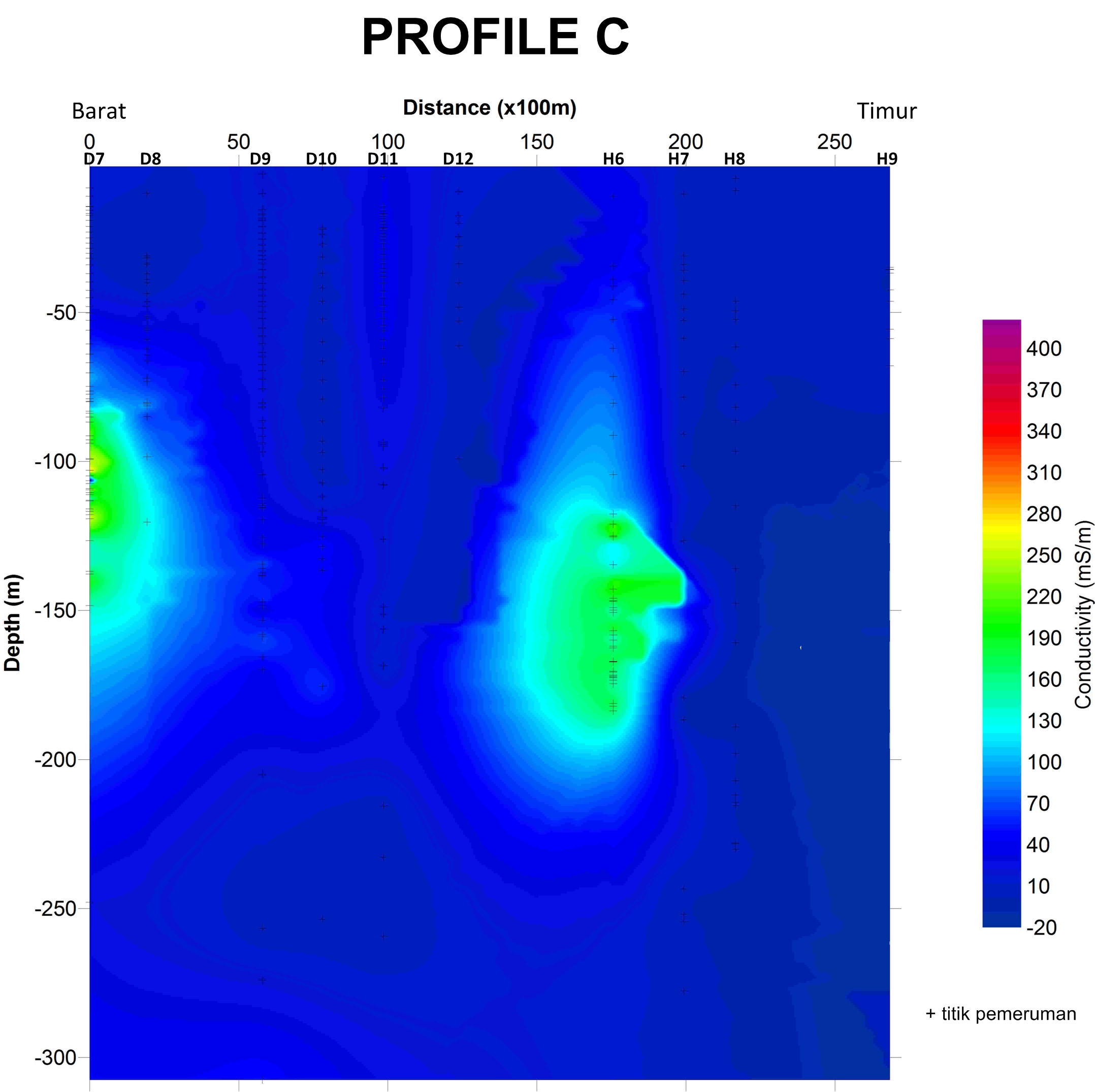

Supplement: Supplementary file 44 [file mmc44.jpg]

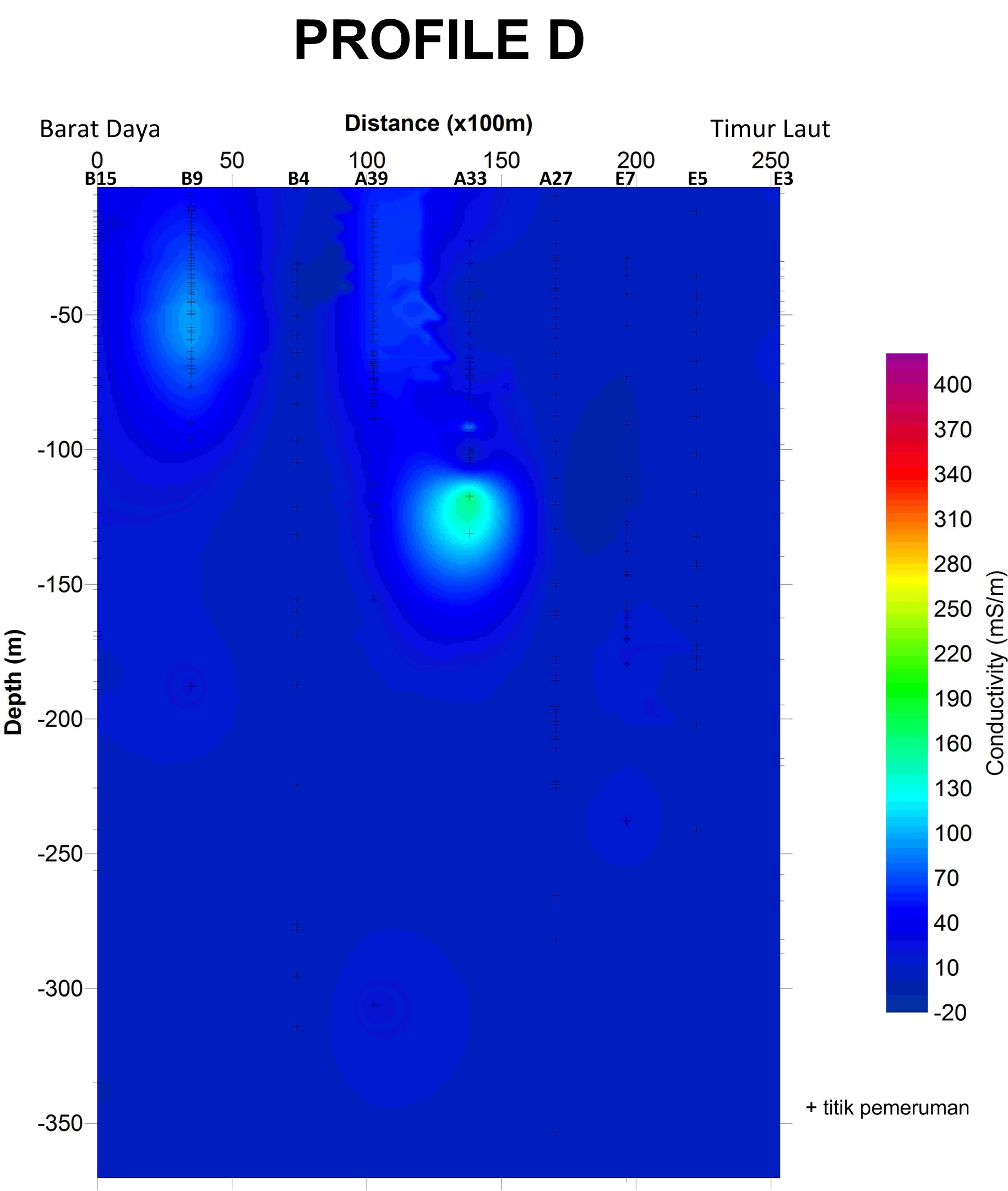

Supplement: Supplementary file 45 [file mmc45.jpg]

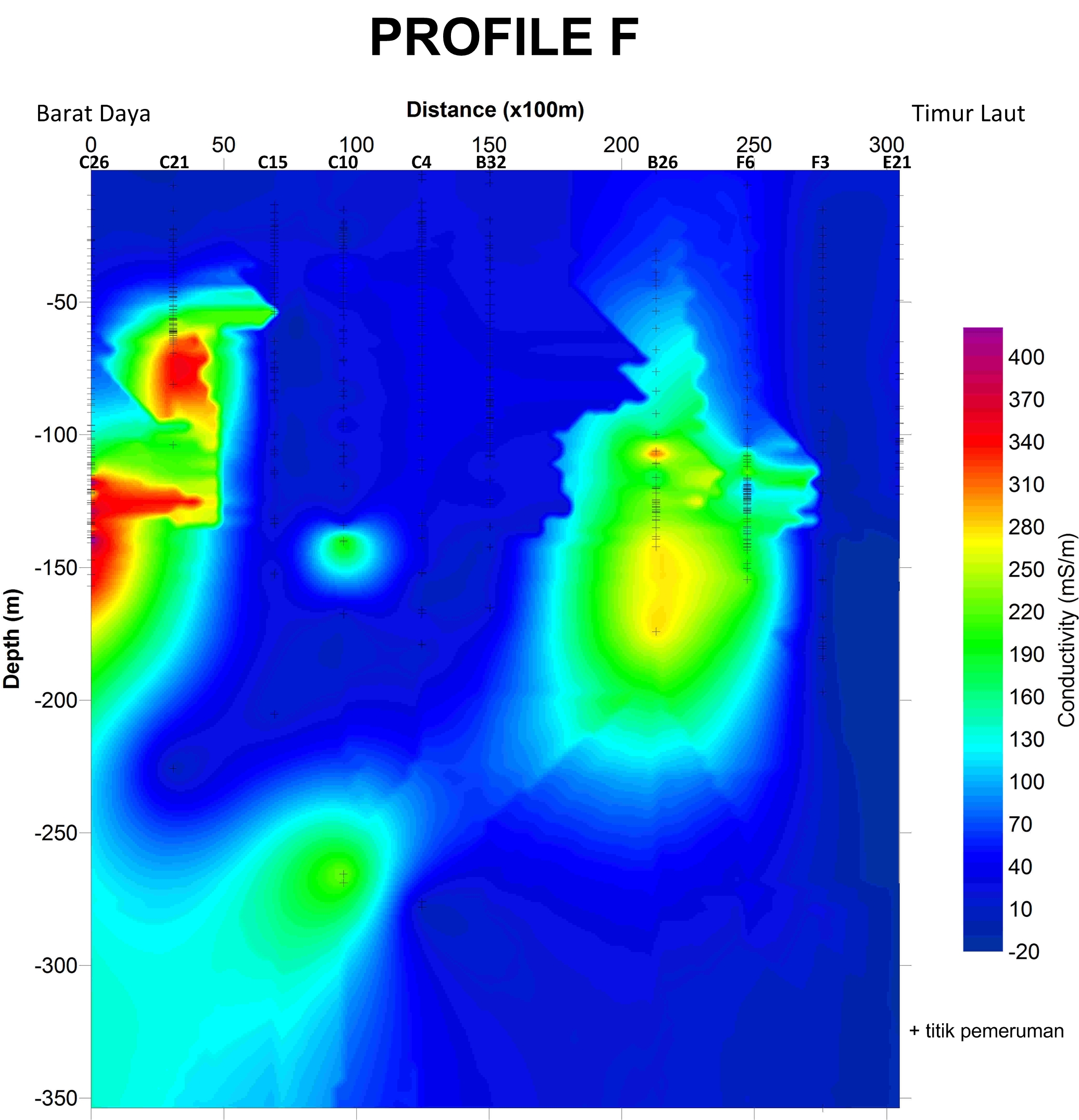

Supplement: Supplementary file 46 [file mmc46.jpg]

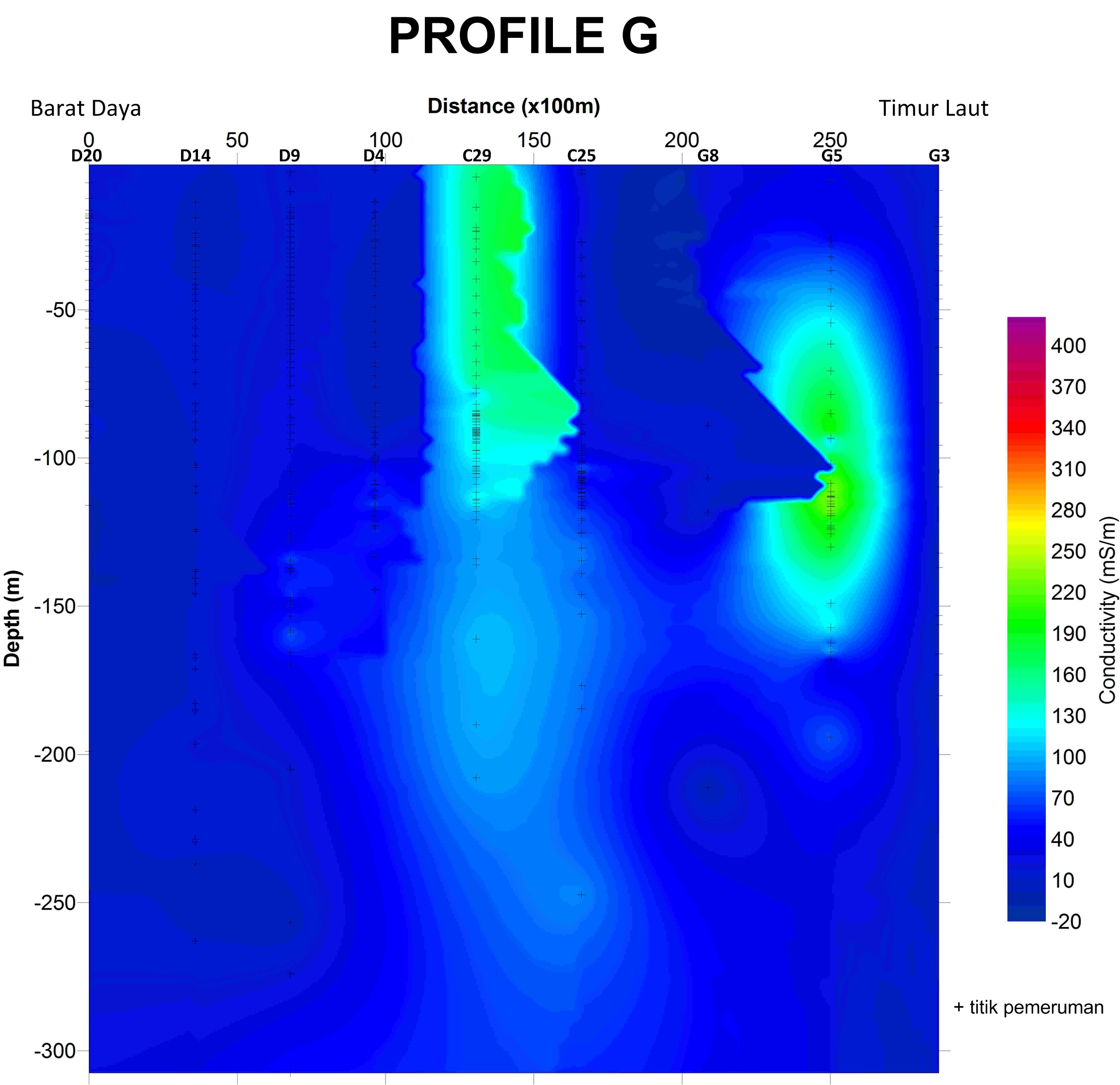

Supplement: Supplementary file 47 [file mmc47.jpg]

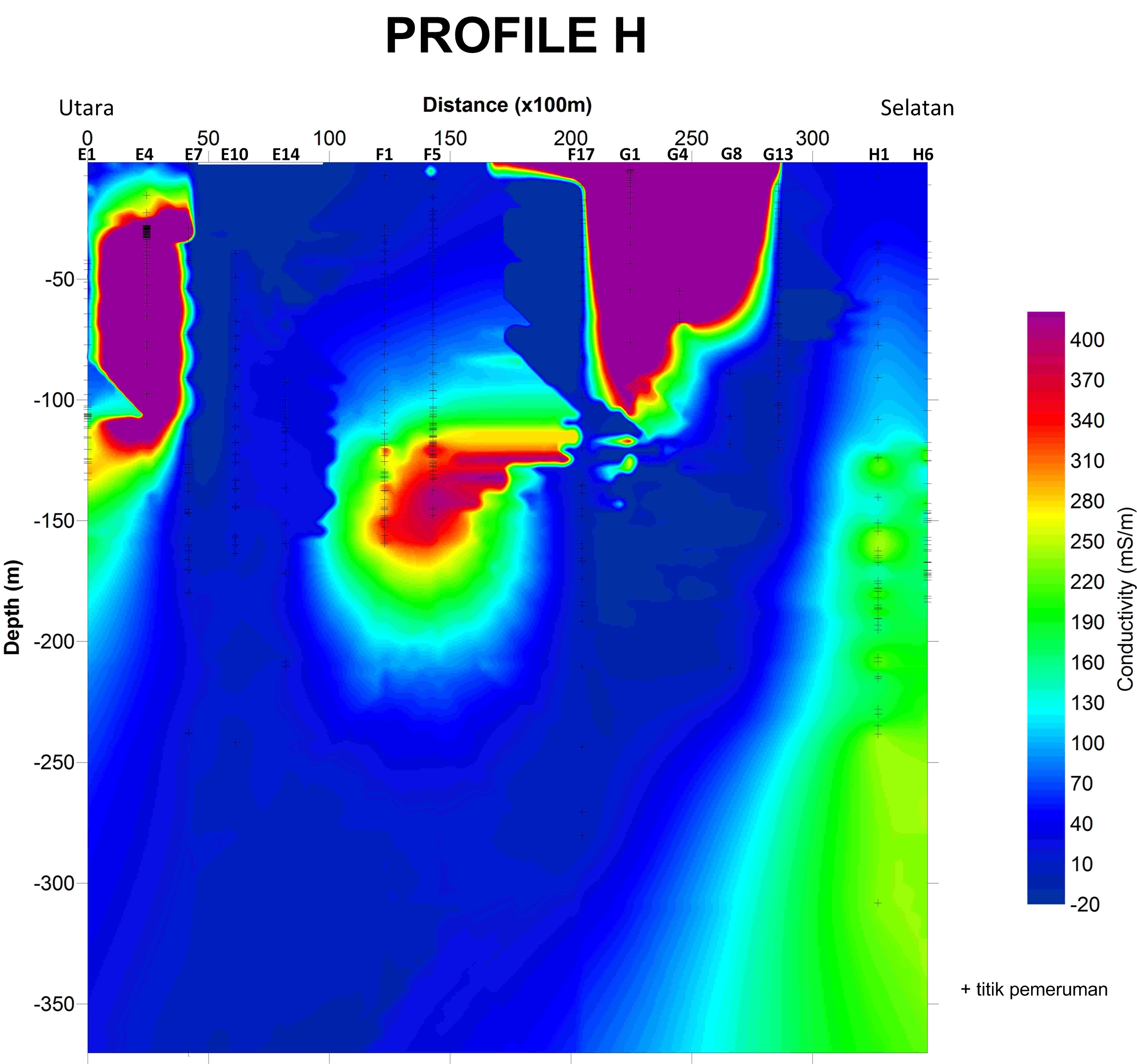

Supplement: Supplementary file 48 [file mmc48.jpg]

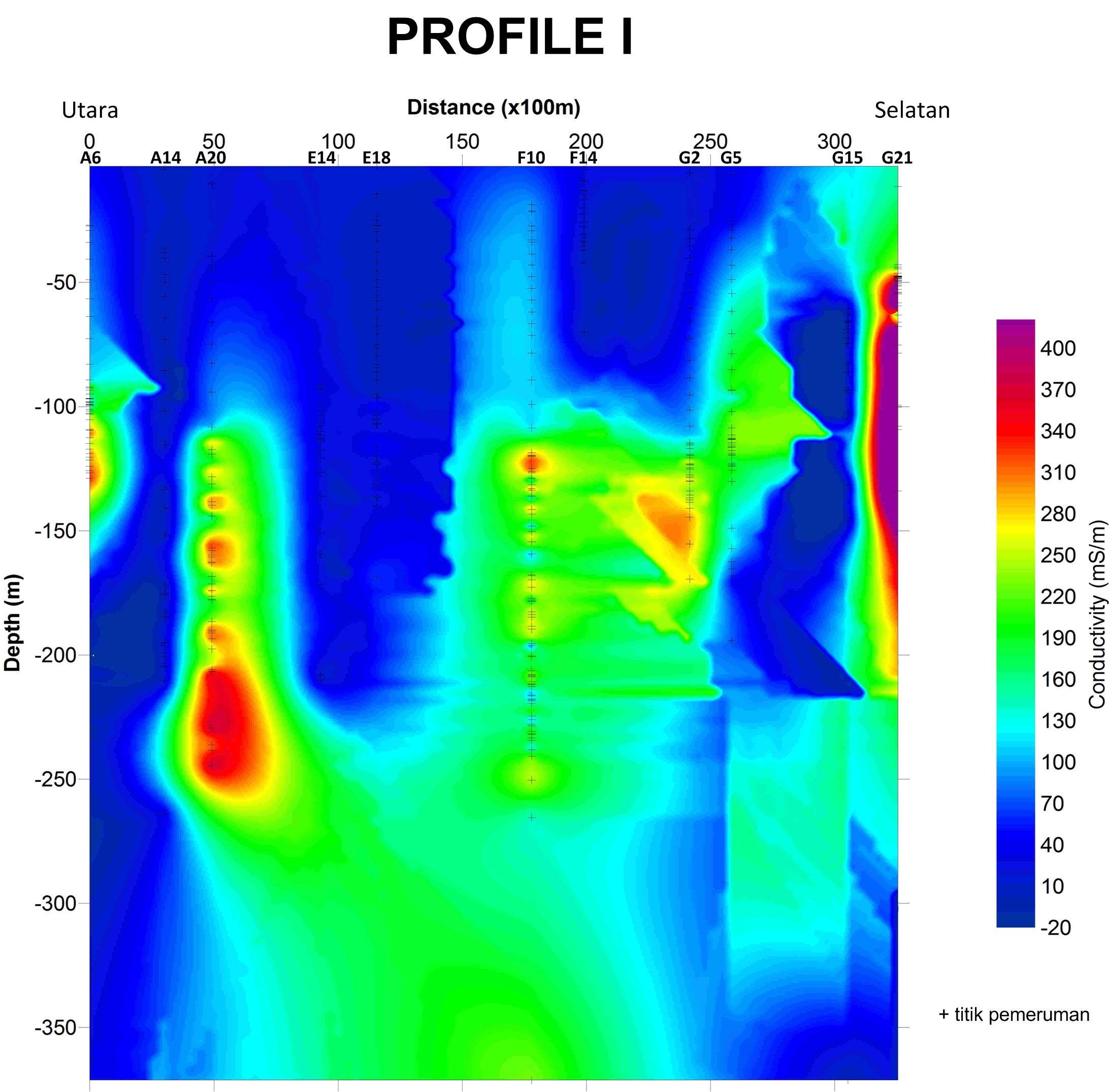

Supplement: Supplementary file 49 [file mmc49.jpg]
